# Characterisation of equine satellite cell transcriptomic profile response to *β*-hydroxy-*β*-methylbutyrate (HMB)

**DOI:** 10.1017/S000711451600324X

**Published:** 2016-10-03

**Authors:** Katarzyna A. Szcześniak, Anna Ciecierska, Piotr Ostaszewski, Tomasz Sadkowski

**Affiliations:** Department of Physiological Sciences, Faculty of Veterinary Medicine, Warsaw University of Life Science – SGGW, Nowoursynowska 159, 02-776 Warsaw, Poland

**Keywords:** *β*-Hydroxy-*β*-methylbutyrate, Satellite cells, Transcriptomic profile, Muscles, Horses

## Abstract

*β*-Hydroxy-*β*-methylbutyrate (HMB) is a popular ergogenic aid used by human athletes and as a supplement to sport horses, because of its ability to aid muscle recovery, improve performance and body composition. Recent findings suggest that HMB may stimulate satellite cells and affect expressions of genes regulating skeletal muscle cell growth. Despite the scientific data showing benefits of HMB supplementation in horses, no previous study has explained the mechanism of action of HMB in this species. The aim of this study was to reveal the molecular background of HMB action on equine skeletal muscle by investigating the transcriptomic profile changes induced by HMB in equine satellite cells *in vitro*. Upon isolation from the *semitendinosus* muscle, equine satellite cells were cultured until the 2nd day of differentiation. Differentiating cells were incubated with HMB for 24 h. Total cellular RNA was isolated, amplified, labelled and hybridised to microarray slides. Microarray data validation was performed with real-time quantitative PCR. HMB induced differential expressions of 361 genes. Functional analysis revealed that the main biological processes influenced by HMB in equine satellite cells were related to muscle organ development, protein metabolism, energy homoeostasis and lipid metabolism. In conclusion, this study demonstrated for the first time that HMB has the potential to influence equine satellite cells by controlling global gene expression. Genes and biological processes targeted by HMB in equine satellite cells may support HMB utility in improving growth and regeneration of equine skeletal muscle; however, the overall role of HMB in horses remains equivocal and requires further proteomic, biochemical and pharmacokinetic studies.

The domestic horse, *Equus Caballus*, is an evolutionary successor of grazing herbivores, whose survival was closely related to the speed and endurance necessary to escape predators and search for food. Since its domestication, man has used selective breeding to enhance performance capabilities of equids, so that they can fulfil their important role in human civilisation^(^ ^1^ ^)^. This has made the horse a valuable animal model for studying exercise physiology.

In modern days, the horse has become an extraordinary ‘athlete’, exercised for a broad range of sporting activities (racing, endurance rides, show jumping, dressage, 3-d eventing, heavy draught work, polo, reining, cutting and competitive driving, as well as pleasure riding)^(^ ^1^ ^)^, which may be associated with serious muscle overloading and an increased risk of injuries. This concerns especially the top-level competitors that are exposed to maximal training loads to achieve even a tiny increase in performance; however, even this small edge over competitors may result in winning the competition^(^ ^2^ ^)^.

This explains the growing demand for alternative treatments that may help improve equine muscle performance and avoid injury. One of these is supplementation with *β*-hydroxy-*β*-methylbutyrate (HMB), a metabolite of the essential branched-chain amino acid leucine^(^ ^3^ ^)^. The benefits of HMB supplementation on muscle metabolism have been demonstrated in various species, under physiological as well as pathological conditions^(^ ^3^ ^,^ ^4^ ^)^. Previous studies have indicated that HMB may affect muscle metabolism and growth by at least six different mechanisms of action, including attenuation of protein degradation^(^ ^5^ ^)^, increased protein synthesis^(^ ^6^ ^)^, protection of sarcolemma^(^ ^7^ ^)^, inhibition of apoptosis^(^ ^8^ ^)^, enhancement of somatotrophic axis function^(^ ^9^ ^)^ and myogenic cell activation^(^ ^10^ ^)^. Recent evidence has indicated additional benefits of HMB supplementation related to energy metabolism, including improved aerobic performance^(^ ^11^ ^)^ as well as increased fat loss with exercise^(^ ^12^ ^)^; however, the underlying mechanisms are poorly understood.

Despite the large amount of literature linked to HMB, only two reports have supported anecdotal data showing HMB’s benefits in thoroughbred racing horses. In one of them, exercising thoroughbred race horses receiving daily 15 g Ca salt of HMB during a 16-week training season showed a significant decrease in post-exercise blood creatinine phosphokinase and lactate levels over both training and racing seasons^(^ ^13^ ^)^. Miller *et al*.^(^ ^14^ ^)^ observed similar results when supplementing racing horses with 10 g of HMB daily, with a significantly improved win rate after the 1st month of racing. Taken together, the present experiment meets the demand for more detailed studies concerning HMB’s effectiveness in horses.

In adult skeletal muscle, regeneration and hypertrophy depend on the activation of mononucleated, muscle precursor cells called satellite cells (SC)^(^ ^15^ ^)^, embedded between the sarcolemma and the basement membrane of muscle fibres. Previous *in vitro* and *in vivo* studies indicate that HMB may activate SC^(^ ^8^ ^,^ ^10^ ^,^ ^16^ ^,^ ^17^ ^)^, but the mechanism underlying this action remains unclear. Some evidence suggests that HMB regulates the expression of myogenesis-related genes^(^ ^8^ ^)^; however, until now, no one has demonstrated any effect of HMB on global gene expression.

The horse is a valuable animal model for studying exercise physiology. Gene expression determines most of the phenotype; therefore, the present study focused on revealing the molecular background of HMB action in equine skeletal muscle by investigating the impact of HMB on global gene expression in differentiating equine satellite cells (ESC) *in vitro*. To our knowledge, this is the first study where HMB’s trancriptomic profile was described. This *in vitro* model can help identify and better understand the potential therapeutic options to promote muscle regeneration and energy metabolism in horses and other mammals.

## Methods

### Cell culture

#### Media and reagents

The following materials were used during cell culture: the Ca salt (monohydrate) of HMB (Ca-HMB) was purchased from Metabolic Technologies; Dulbecco’s Modified Eagle Medium (DMEM) (1×) with glutamax, fetal bovine serum (FBS), horse serum (HS) and antibiotics (AB) **–** penicillin–streptomycin and fungizone – were purchased from Gibco, Life Technologies; penicillium crystalicum (AB) was purchased from Polfa Tarchomin; PBS, protease from *Streptomyces griseus* and DMSO were purchased from Sigma Aldrich. Tissue culture flasks Primaria (25, 75 cm^2^) and Collagen I Cellware six-well plates were purchased from Becton Dickinson. Ca-HMB was transformed to the acid form by acidification with 1 N-HCl. HMB was then extracted four times with diethyl ether. The pooled organic layer was dried under vacuum for 24 h at 38 °C. The resulting free acid was 99 % HMB as assessed by HPLC.

#### Muscle sampling and satellite cells isolation

*Semitendinosus* muscle samples were collected *ex vivo* from six horses (6-month-old, healthy colts). Muscle sampling and ESC isolation are described in detail by Szcześniak *et al*.^(^ ^18^ ^)^. In brief, *semitendinosus* muscle samples were dissected free of surrounding tissues, sliced, washed in PBS with decreasing antibiotics concentration, suspended in FBS with 10 % DMSO, cooled to −80°C and stored in liquid N_2_. Before isolation, the samples were thawed, centrifuged and washed three times with PBS along with antibiotics. Samples were incubated with DMEM/AB/protease from *S. griseus* and sieved in order to separate tissue debris. The filtrates were centrifuged three times, re-suspended in proliferation medium (10 %FBS/10 %HS/DMEM/AB) and transferred to polypropylene Petri culture disks. One-and-a-half hours of preplating was performed to minimise possible fibroblast contamination. Subsequently, the supernatant containing ESC was transferred to Primaria culture flasks.

#### Cell culture and experimental design

**Fig. 1:**
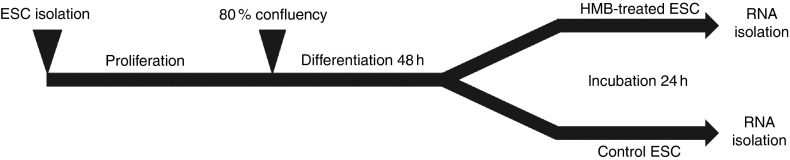
Experiment design. Equine satellite cells (ESC) were cultured until they reached 80 % confluence; next, the proliferation medium was replaced with a differentiation medium. After the 2nd day of differentiation, cells were incubated for 24 h with *β*-hydroxy-*β*-methylbutyrate (HMB). Following the HMB treatment, differentiating cells were scraped and stored at −80°C until further analysis.

The experimental design is presented in Fig. 1. Upon isolation, samples of ESC (*n* 6) were incubated for 10 d in Primaria culture flasks. The proliferation medium was changed every 2 d. On the 10th day, cells were trypsinised, and 30 000 cells (counted by Scepter Cell Counter; Merck Millipore) from each flask were transferred to the respective wells of two six-well plates. One plate was dedicated to HMB treatment and one served as the control. After obtaining 80 % of confluence, the proliferation medium was replaced with a differentiation medium (2 % HS/DMEM/AB). Immediately after 48 h of differentiation, the medium from one plate was replaced by a differentiation medium containing 50 µm of HMB, whereas in the second plate the standard differentiation medium was used as a control. After 24 h, the medium from each plate was discarded, plates were washed with PBS and stored at −80°C until further analysis. The concentration of HMB was based on the available literature values and cell viability colourimetric assay test with 3-(4,5-dimethylthiazol-2-yl)-2-5-diphenyltetrazolium bromide (data not shown).

### Microarray analysis and real-time quantitative PCR validation

#### RNA isolation, validation, labelling hybridisation and microarray analysis

Total RNA from HMB and control cells was isolated according to the protocol supplied with the miRNeasy Mini Kit (Qiagen). RNA quantity was measured spectrophotometrically using NanoDrop (NanoDrop Technologies). The analysis of final RNA quality and integrity was performed with BioAnalyzer 2100 (Agilent Technologies). To ensure optimal microarray data quality, only samples with the highest RNA integrity number (RIN)≥9·2 were included in the analysis.

Analysis of gene expression profiles was performed using *Horse Gene Expression Microarray*, 4×44K (Agilent Technologies). Low Input Quick Amp Labeling Kit (Agilent Technologies) was used to amplify and label total RNA (100 ng) to generate complementary RNA (cRNA). On each two-colour microarray, 825 ng of cRNA from HMB-exposed cells (labelled by Cy5, *n* 4) and 825 ng of cRNA from control cells (labelled by Cy3, *n* 4) were hybridised to the arrays (Gene Expression Hybridization Kit; Agilent Technologies) according to the manufacturer’s protocol.

RNA Spike-In Kit (Agilent Technologies) was used as an internal control to efficiently monitor microarray workflow for linearity, sensitivity and accuracy. Acquisition and analysis of hybridisation intensities were performed using the DNA microarray scanner (Agilent Technologies) and Feature Extraction software 10.7.3.1 according to the standard manufacturer’s procedures. Linear Lowess was applied for data normalisation and Cy5/Cy3 dye bias compensation.

### Statistical analysis

Statistical analysis was performed using Gene Spring 13 software (Agilent Technologies) with the default setting for two-colour microarrays. The estimated significance level (*P* value) was corrected for multiple hypotheses testing using the Benjamini and Hochberg false discovery rate (FDR) adjustment. mRNA with FDR≤0·05 were selected as significantly differentially expressed genes (DEG).

The microarray experiment was performed according to Minimum information about a microarray experiment (MIAME) guidelines^(^ ^19^ ^)^. The data discussed in this publication have been deposited in National Center for Biotechnology Information’s (NCBI’s) Gene Expression Omnibus (GEO)^(^ ^20^ ^)^ and are accessible through GEO Series accession number GSE74495 (http://www.ncbi.nlm.nih.gov/geo/query/acc.cgi?acc=GSE74495).

#### Complementary DNA synthesis and real-time quantitative PCR

To independently assess expression changes for a selected group of genes obtained from the microarray data, the real-time quantitative PCR (RT-qPCR) method was applied. The sequences of verified genes, complementary to those on microarrays, were obtained from Ensembl database. Primers were designed using Primer-Blast software (NCBI database) and then checked for secondary structures using the Oligo Calculator (http://www.basic.northwestern.edu/biotools/oligocalc.html). The secondary structures of the amplicon were examined using m-fold Web Server (http://mfold.rna.albany.edu/?q=mfold). The sequences of primers are listed in Table 1. The primers were purchased from Oligo IBB (Polish Academy of Science). Each primer pair was quality tested to ensure that a single product was amplified (dissociation curve analysis) and that there was no primer–dimer coupling.

**Table 1:** Sequences of primers used for real-time quantitative PCR

No.	Gene symbol	Forward primer	Reverse primer	Annealing temperature (ºC)	Product lenght
1	*Cfl2*	CCCGCAGAGTTGACACAATA	TGTGGCATCGTACAAAGCAT	60	282
2	*Myf5*	GGAGACGCCTGAAGAAAGTC	CCGGCAGGCTGTAGTAATTC	60	171
3	*Rbfox*	GAACCAGGAGGGATCTTCCA	TTGCCATACACAGGCTCTTG	60	213
4	*S1pp1*	CCCAAGTCAGTCCAACGAAA	GGCACAGCTGGTGTAAAAAC	60	143
5	*Tgfb2*	AGTACTACGCCAAGGAGGTT	TAGGCGGGATGGCATTTTCC	60	72
6	*Trim63*	AAGGAGGCAGCCAGGTAGAG	CACGGACACTGAGCCACTTC	62	220
7	*Gapdh*	GTTTGTGATGGGCGTGAACC	GTCTTCTGGGTGGCAGTGAT	60	198

*Cfl2*, coffilin 2; *Myf5*, myogenic factor 5; *Rbfox*, RNA binding protein, fox-1 homolog *C. elegans*; *S1pp1*, secreted phosphoprotein 1; *Tgfb2*, transforming growth factor, *β*2; *Trim63*, muscle-specific RING finger protein 1; *Gapdh*, glyceraldehyde 3-phosphate dehydrogenase.

A quantity of 1 µg of total RNA from HMB-treated and control cells (*n* 6) was reverse transcribed using a Transcription First Strand cDNA Synthesis Kit (Agilent Technologies). All analyses were performed on individual samples of total RNA using a SensiFAST SYBR lo-ROX Kit (Blirt, Bioline) following the manufacturer’s protocol. Assays for each gene were conducted in duplicate in a Stratagene Mx3005p thermal cycler (Agilent Technologies) according to the following protocol: pre-incubation for 2 min at 95°C and amplification (forty cycles), with denaturation at 95°C for 5 s and annealing at the temperatures specified in Table 1 for 15 s. The dissociation curve setting was as follows: denaturation at 95°C for 0 s, annealing (at the temperatures specified in Table 1), continuous melting up to 95°C for 0 s (slope=0·1°C/s) and cooling at 40°C for 30 s. Glyceraldehyde 3-phosphate dehydrogenase (*Gapdh*) was used as a reference gene. The relative expression of the target gene was calculated according to the following formula:
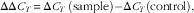
where Δ*C* 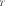 is the difference in *C*_*T*_ between the targeted gene and the reference control. Results were calculated as 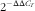 using GenEx 6.0 (MultiD Analyses)^(^ ^21^ ^)^. The amplification efficiency (*E*=10^(−1/slope)^–1) was determined using a comparative quantitation standard curve and was >0·9 for each target gene and the reference gene. Standard curves were generated using a four-point 1:10 dilution series starting with cDNA representing 10 ng of input total RNA. RT-qPCR analysis was conducted according to a standardised approach^(^ ^22^ ^)^.

### Functional analysis

The list of DEG was examined by the Functional Analysis tool in the Database for Annotation, Visualization and Integrated Discovery (DAVID version 6.7) to assign them to gene ontology (GO) terms and KEGG pathways (Kyoto Encyclopedia of Genes and Genomes)^(^ ^23^ ^)^. Human background was used for this analysis, because far more human genes are annotated and more information in databases is available for humans than for horses. Enrichment of DEG was calculated by EASE score (modified Fisher exact test). For further analysis and visualisation of data, the Pathway Studio Web Mammalian was used. This database of functional relationships between mammalian proteins is compiled using Med Scan technology from over twenty-four million PubMed abstracts and over 3·5 million Elsevier full-text papers. All identified relations were filtered by reference count (≥2) to ensure maximal confidence levels, which means that the number of publications confirming each relationship was ≥2.

## Results

### Microarray analysis

Analysis of gene expression between HMB-treated and control cells revealed statistically significant (FDR≤0·05) differences in the case of 627 records. Within them were 361 unduplicated, identified transcript ID including 159 up- and 202 down-regulated DEG, in the HMB *v*. the control group. All array data are plotted and shown in the online Supplementary Material S1. Table 2 presents genes selected for discussion, presumably involved in HMB action on ESC.

**Table 2:** List of selected differentially expressed genes in *β*-hydroxy-*β*-methylbutyrate-treated *v*. control equine satellite cells (false discovery rate≤0·05, *n* 4)

No.	Gene symbol	Fold change	Description	False discovery rate (corrected *p*-value)
1	*Nos2*	−2·43	Inducible nitric oxide synthase (NM_001081769)	4·34E–2
2	*Myf5*	−2·09	Myogenic factor 5 (ENSECAT00000021416)	4·63E–2
3	*Dmd*	−2·06	Dystrophin (ENSECAT00000023688)	3·18E–2
4	*Trim63*	−2·02	Tripartite motif containing 63, E3 ubiquitin protein ligase (ENSECAT00000026380)	4·96E–2
5	*Itgb1bp2*	−1·94	Integrin *β* 1 binding protein (melusin) 2 (ENSECAT00000016364)	4·52E–2
6	*Saa1*	−1·88	Serum amyloid A1 (ENSECAT00000013971)	4·96E–2
7	*Tagln3*	−1·80	Transgelin 3 (ENSECAT00000010210)	4·73E–2
8	*Tgfb2*	−1·75	Transforming growth factor, *β*2 (XM_003364564·2)	3 31E–2
9	*Murc*	−1·69	Muscle-related coiled-coil protein (ENSECAT00000006670)	4·76E–2
10	*Svil*	−1·66	Supervillin (XM_014737013·1)	4·88E–2
11	*Lama2*	−1·60	Laminin, *α*5 (XM_014735356·1)	3·18E–2
12	*Mef2c*	−1·56	Myocyte enhancer factor 2 C (XM_014857076·1)	3·56E–2
13	*Lama2*	−1·42	Laminin, *α*2 (ENSECAT00000025657)	3·96E–2
14	*Prkab2*	−1·42	Protein kinase, AMP-activated, *β*2 non-catalytic subunit (XM_008509324·1)	4·65E–2
15	*Mef2a*	−1·32	Myocyte enhancer factor 2A (XM_011521571·1)	4·76E–2
16	*Ppargc1b*	−1·22	PPAR-*γ* coactivator (ENSECAT00000021080)	4·75E–2
17	*Cul3*	−1·17	Cullin 3 (ENSECAT00000012128)	4·67E–2
18	*Esrra*	−1·13	Oestrogen-related receptor *α* (ENSECAT00000016651)	4·31E–2
19	*Zfp91*	−1·10	Zinc finger protein 91 homolog (XM_005598160)	3·95E–2
20	*Abca1*	1·79	ATP-binding cassette, sub-family A, member 1 (XM_001493790)	3·87E–2
21	*Mapk14*	1·75	Mitogen-activated protein kinase 14 (XM_005604060)	4·89E–2
22	*F2rl2*	1·65	Coagulation factor II (thrombin) receptor-like 2 (ENSECAT00000010830)	4·49E–2
23	*Fads1*	1·33	Fatty acid desaturase 1 (XM_008510001)	4·96E–2
24	*Abhd5*	1·24	Anhydrolase domain containing 5 (ENSECAT00000023610)	3·96E–2

### Real-time quantitative PCR

**Fig. 2:**
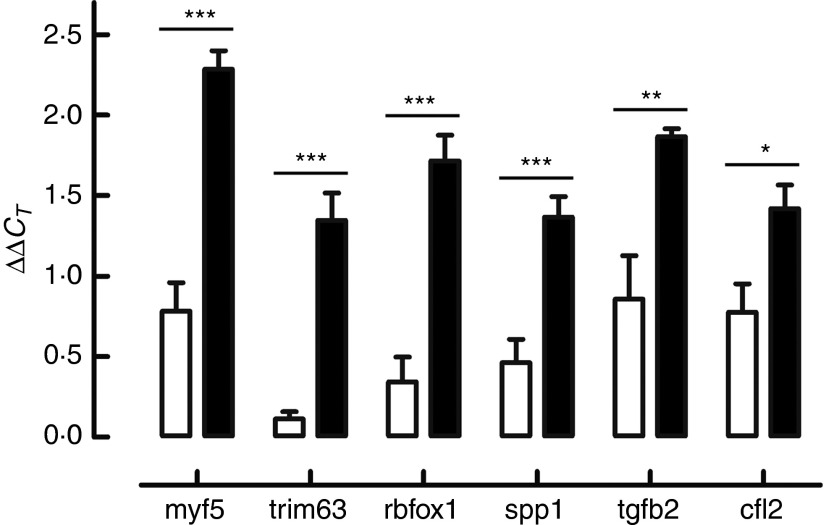
Genes selected for real-time quantitative PCR (RT-qPCR) validation of microarray results: *Cfl2* (coffilin 2, muscle), *Myf5* (myogenic factor 5), *Rbfox* (RNA binding protein, fox-1 homolog *C. elegans*), *S1pp1* (secreted phosphoprotein 1), *Tgfb2* (transforming growth factor, *β*2) and *Trim63* (muscle-specific RING finger protein 1). Expression changes from RT-qPCR data overlapped microarray results. * *P*≤0·05, ** *P*≤0·01, *** *P*≤0·001 are significant (*n* 6). 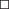, *β*-hydroxy-*β*-methylbutyrate (HMB); 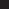, Ctrl.

According to the ontological classification and the literature, six genes – *Cfl2* (coffilin 2, muscle), *Myf5* (myogenic factor 5), *Rbfox* (RNA binding protein, fox-1 homolog *C. elegans*), *S1pp1* (secreted phosphoprotein 1), *Tgfb2* (transforming growth factor, *β*2) and *Trim63* (muscle-specific RING finger protein 1) involved in the skeletal muscle development – were selected for RT-qPCR validation. Expression changes from RT-qPCR data overlapped microarray results and are presented in Fig. 2.

### Functional analysis

DAVID functional analysis assigned DEG to seventy-five biological processes (BP), eleven cellular components and ten molecular functions as well as four KEGG pathways (EASE score *P*<0·05). All GO considered significant are shown in the online Supplementary Material S2. KEGG pathways and the most significantly enriched (EASE score <0·01) GO retrieved from DAVID are presented in Table 3, providing a comprehensive overview of important processes, most likely induced by HMB in differentiating ESC.

**Table 3:** Functional analysis of differentially expressed genes*

GO					
Categories	Term	Count	%	*P*	Genes
Biological process	GO:0007517 – muscle organ development	14	4·12	2·31E–4	*Mef2c*, *Mef2a*, *Myf5*, *Tagln3*, *Tgfb2*, *Lama2*, *Zfp91*, *Murc*, *Lama5*, *Mapk14*, *Svil*, *Dmd*, *Itgb1bp2*, *F2r*
Cellular component	GO:0005829 – cytosol	46	13·53	3 84E–4	*Bcat1*, *Alad*, *Ggct*, *Tnfrsf25*, *Abhd5*, *Kcnip3*, *Rps3*, *Cep70*, *Zfp91*, *Rps26*, *Bag1*, *Slmap*, *Hnrnpd*, *Gucy1a3*, *Eif3i*, *Nos2*, *Rpia*, *Psmd6*, *Plcb1*, *Gchfr*…
Biological process	GO:0009987 – cellular process	227	66·76	5·32E–4	*Mef2c*, *Mef2a*, *Alad*, *Tars2*, *Fst*, *Gfer*, *Lpar2*, *Edil3*, *Rest*, *Tpd52*, *Prkg1*, *S1pr2*, *Cul3*, *Zfp91*, *Hmcn1*, *Kifap3*, *Sfrs9*, *Scd5*, *Nsmaf*, *Rpp21*…
Biological process	GO:0048518 – positive regulation of biological process	58	17·06	1·94E–3	*Mef2c*, *Fosl2*, *Fst*, *Tlr1*, *Lpar2*, *Pmaip1*, *Edil3*, *Gli1*, *Tgfb2*, *Rps3*, *Cul3*, *S1pr2*, *Zfp91*, *Mll5*, *Ang*, *Saa1*, *Kifap3*, *Gucy1a3*, *Nos2*, *Psmd6*…
Biological process	GO:0050793 – regulation of developmental process	25	7·35	2·80E–3	*Gna12*, *Fst*, *Abca1*, *Rest*, *Gli1*, *Tgfb2*, *Zfp91*, *Cdc42ep3*, *Nkx2-2*, *Spp1*, *B4galt1*, *Esrra*, *Foxj1*, *Fads1*, *Smad5*, *Mgp*, *Ski*, *Smad1*, *Sod2*, *Lama2…*
Biological process	GO:0044267 – cellular protein metabolic process	64	18·82	3·37E–3	*Gnptg*, *Cdk19*, *Ilkap*, *Tars2*, *Kiaa0368*, *Lpar2*, *Prkg1*, *Ttll1*, *Tgfb2*, *Rps3*, *S1pr2*, *Cul3*, *Mll5*, *Hmcn1*, *Pak3*, *Map1lc3b*, *Aak1*, *Slmap*, *St3gal6*, *Stk39*…
Biological process	GO:0030278 – regulation of ossification	7	2·06	3·90E–3	*Esrra*, *Smad5*, *Mgp*, *Gdf10*, *Ski*, *Smad1*, *Tgfb2*
Biological process	GO:0051239 – regulation of multicellular organismal process	31	9·12	4·27E–3	*Tlr1*, *Fst*, *Rest*, *Tpm3*, *Kcnmb2*, *Tgfb2*, *Gli1*, *Zfp91*, *chd7*, *Saa1*, *Arg2*, *Gucy1a3*, *Nos2*, *Kcnq1*, *Nkx2-2*, *Spp1*, *B4galt1*, *Esrra*, *Foxj1*, *Smad5*…
Biological process	GO:0030155 – regulation of cell adhesion	9	2·65	5·05E–3	*Lama2*, *Cytip, Saa1, Lama5, Kifap3, Myf5, Edil3, Spp1, Tgfb2*
Biological process	GO:0048522 – positive regulation of cellular process	51	15·00	7·59E–3	*Mef2c*, *Fosl2*, *Tlr1*, *Lpar2*, *Pmaip1*, *Edil3*, *Tgfb2*, *Gli1*, *Rps3*, *Cul3*, *S1pr2*, *Zfp91*, *Mll5*, *Saa1*, *Ang*, *Kifap3*, *Gucy1a3*, *Psmd6*, *Samd4a*, *Ip6k2*
Biological process	GO:0051345 – positive regulation of hydrolase activity	10	2·94	7·94E–3	*Uaca, Ang, Gnb1, Foxj1, Abhd5, Arhgap27, Lpar2, Pmaip1, Rps3, F2r*
Biological process	GO:0009891 – positive regulation of biosynthetic process	24	7·06	8·19E–3	*Mef2c*, *Esrra*, *Tp53bp1*, *Myf5*, *Smad5*, *Tlr1*, *Abca1*, *Smad1*, *Ppargc1b*, *Sod2*, *Gli1*, *Tgfb2*, *Murc*, *Mll5*, *Mapk14*, *Gucy1a3*, *Prkaa1*, *Hoxb9*, *Rnf10*, *Smarca2*, *Nfatc3*, *Nkx2-2*, *Samd4a*, *F2r*…
Cellular component	GO:0022627 – cytosolic small ribosomal subunit	5	1·47	8·49E–3	*Rps26*, *Rps18*, *Rps14*, *Rps12*, *Rps3*
Biological process	GO:0060341 – regulation of cellular localisation	12	3·53	8·78E–3	*B4galt1*, *Zfp91*, *Uaca*, *Chd7*, *Saa1*, *Ang*, *Pkig*, *Fst*, *Nos2*, *Kcnq1*, *Calm1*, *Tgfb2*
Cellular component	GO:0015935 – small ribosomal subunit	6	1·76	8·88E–3	*Rps26*, *Rps18*, *Rps14*, *Mrps24*, *Rps12*, *Rps3*
Molecular functions	GO:0030145 – manganese ion binding	9	2·65	9·69E–3	*B4galt1*, *Ilkap*, *B4galt3*, *Arg2*, *Smg1*, *Ppp1cc*, *B4galt7*, *Galnt12*, *Sod2*
KEGG pathways					
Terms		Count	%	*P*	Genes
hsa01040: biosynthesis of unsaturated fatty acids		4	1·18	1·0E–2	*Acot7*, *Fads1*, *Hacd1*, *Scd5*
hsa00601: glycosphingolipid biosynthesis		4	1·18	2·0E–2	*B4galt1*, *B4galt3*, *B3gnt5*, *St3gal6*
hsa04270: vascular smooth muscle contraction		7	2·06	4·0E–2	*Gna12*, *Gucy1a3*, *Prkg1*, *Ppp1cc*, *Plcb1*, *Calm1*, *Kcnmb2*
hsa05410: hypertrophic cardiomyopathy		6	1·76	4·0E–2	*Lama2*, *Dmd*, *Prkab2*, *Prkaa1*, *Tgfb2*, *Tpm3*

GO, gene ontology; KEGG, Kyoto Encyclopedia of Genes and Genomes; *Aak1*, AP2 associated kinase 1; *Abca1*, ATP-binding cassette, sub-family A, member 1; *Abhd5*, abhydrolase domain containing 5; *Acot7*, acyl-CoA thioesterase 7; *Alad*, aminolevulinate dehydratase; *Ang*, angiogenin, ribonuclease, RNase A family, 5; *Arg2*, arg2; *Arhgap27*, rho GTPase activating protein 27; *B3gnt5*, β-1,3-N-acetylglucosaminyltransferase 5; *B4galt1*, β-1,4-galactosyltransferase 1; *B4galt3*, β-1,4-galactosyltransferase 3; *B4galt7*, β-1,4-galactosyltransferase 7; *Bag1*, BCL2 associated athanogene 1; *Bcat1*, branched chain amino acid transaminase 1; *Calm1*, calmodulin 1 (phosphorylase kinase, delta); *Cdc42ep3*, CDC42 effector protein 3; *Cdk19*, cyclin-dependent kinase 19; *Cep70*, centrosomal protein 70; *Chd7*, chromodomain helicase DNA binding protein 7; *Cul3*, cullin 3; *Cytip*, cytohesin 1 interacting protein; *Dmd*, dystrophin; *Edil3*, EGF Like repeats and discoidin domains 3; *Eif3i*, eukaryotic translation initiation factor 3 subunit I; *Esrra*, estrogen related receptor α; *F2r*, coagulation factor II thrombin receptor; *Fads1*, fatty acid desaturase 1; *Fosl2*, FOS like antigen 2; *Foxj1*, forkhead box J1; *Fst*, follistatin; *Galnt12*, polypeptide N-acetylgalactosaminyltransferase 12; *Gchfr*, GTP cyclohydrolase I feedback regulator; *Gdf10*, growth differentiation factor 10; *Gfer*, growth factor, augmenter of liver regeneration; *Ggct*, γ-glutamylcyclotransferase; *Gli1*, GLI family zinc finger 1; *Gna12*, G protein subunit α 12; *Gnb1*, G protein subunit β 1; *Gnptg*, N-acetylglucosamine-1-phosphate transferase γ subunit; *Gucy1a3*, guanylate cyclase 1, soluble, α 3; *Hmcn1*, hemicentin 1; *Hnrnpd*, heterogeneous nuclear ribonucleoprotein D; *Hoxb9*, homeobox B9; *Ilkap*, ILK associated serine/threonine phosphatase; *Ip6k2*, inositol hexakisphosphate kinase 2; *Itgb1bp2*, integrin subunit β 1 binding protein 2; *Kcnip3*, potassium voltage-gated channel interacting protein 3; *Kcnmb2*, potassium calcium-activated channel subfamily M regulatory β subunit 2; *Kcnq1*, potassium voltage-gated channel subfamily Q member 1; *Kiaa0368*, ECM29 homolog, proteasome accessory protein; *Kifap3*, kinesin associated protein 3; *Lama2;* laminin subunit α 2; *Lama5*, laminin subunit α 5; *Lpar2*, lysophosphatidic acid receptor 2; *Map1lc3b*, microtubule associated protein 1 light chain 3 β; *Mapk14*, mitogen-activated protein kinase 14; *Mef2a*, myocyte enhancer factor 2A; *Mef2c*, myocyte enhancer factor 2C; *Mgp*, matrix Gla protein; *Mll5*, lysine methyltransferase 2E; *Mrps24*, mitochondrial ribosomal protein S24; *Murc*, muscle related coiled-coil protein; *Myf5*, myogenic factor 5; *Nfatc3*, nuclear factor of activated T-cells 3; *Nkx2-2*, NK2 homeobox 2; *Nos2*, nitric oxide synthase 2; *Nsmaf*, neutral sphingomyelinase activation associated factor; *Pak3*, P21 protein (Cdc42/Rac)-activated kinase 3; *Pkig*, protein kinase (CAMP-dependent, catalytic) inhibitor γ; *Plcb1*, phospholipase C β 1; *Pmaip1*, phorbol-12-myristate-13-acetate-induced protein 1; *Ppargc1b*, PPARG coactivator 1 β; *Ppp1cc*, protein phosphatase 1 catalytic subunit γ; *Prkaa1*, protein kinase AMP-activated catalytic subunit α 1; *Prkab2*, protein kinase AMP-activated non-catalytic subunit β 2; *Prkg1*, protein kinase, CGMP-dependent, type I; *Psmd6*, proteasome 26S Subunit, Non-ATPase 6; *Ptpla*, 3-hydroxyacyl-CoA dehydratase 1; *Rest*, RE1 silencing transcription factor; *Rnf10*, ring finger protein 10; *Rpia*, ribose 5-phosphate isomerase A; *Rpp21*, ribonuclease P/MRP subunit P21; *Rps12*, ribosomal protein S12; *Rps14*, ribosomal protein S14; *Rps18*, ribosomal protein S18; *Rps26*, ribosomal protein S26; *Rps3*, ribosomal protein S3; *S1pr2,* sphingosine-1-phosphate receptor 2; *Saa1*, serum amyloid A1; *Samd4a*, sterile α motif domain containing 4A; *Scd5*, stearoyl-CoA desaturase 5; *Sfrs9*, serine/arginine-rich splicing factor 9; *Ski*, SKI proto-oncogene; *Slmap*, sarcolemma associated protein; *Smad1*, SMAD family member 1; *Smad5*, SMAD family member 5; *Smarca2*, SWI/SNF related, matrix associated, actin dependent regulator of chromatin, subfamily a, member 2; *Smg1*, SMG1 phosphatidylinositol 3-kinase-related kinase; *Sod2*, superoxide dismutase 2, mitochondrial; *Spp1*, secreted phosphoprotein 1; *St3gal6*, ST3 β-galactoside α-2,3-sialyltransferase 6; *Stk39*, serine/threonine kinase 39; *Svil*, supervillin; *Tagln3*, transgelin 3; *Tars2*, threonyl-TRNA synthetase 2, mitochondrial (putative); *Tgfb2*, transforming growth factor β 2; *Tlr1*, toll like.

* Most significantly enriched ontologies (*P*<0·01) and KEGG pathways are presented.

**Fig. 3:**
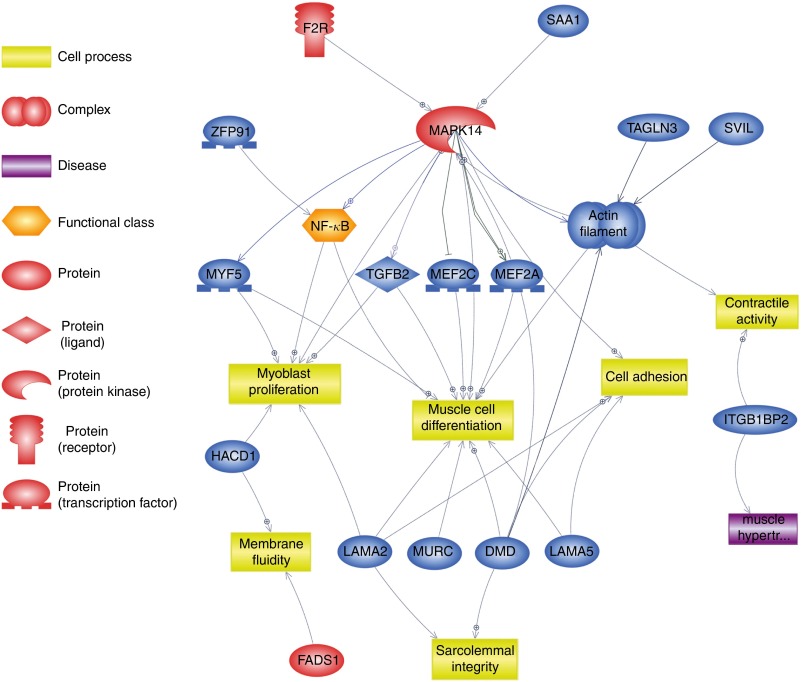
Pathway depicting *β*-hydroxy-*β*-methylbutyrate (HMB)-modulated genes identified in the present analysis, which could directly or indirectly affect skeletal muscle cell functions. This pathway was created using Pathway Studio Web Mammalian. Genes are marked with red and blue colour for up- and down-regulation, respectively. F2R, coagulation factor II; SAA1, serum amyloid A1; TAGLN3, transgelin 3; SVIL, supervilin; MEF2a and MEF2c, myocyte enhancer factor 2a and 2c; TGFB2, transforming growth factor, *β*2; MAPK14, mitogen-activated protein kinase 14; ZFP91, zinc finger protein 91 homolog; MYF5, myogenic factor 5; HACD1, 3-hydroxyacyl-CoA dehydratase 1 (alias PTPLA); LAMA, laminins; MURC, muscle-related coiled-coil protein; DMD, dystrophin; ITGB1BP2, integrin *β*1 binding protein (melusin) 2; 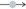, direct regulation; 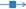, expression; 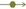, promoter modification; 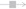, regulation.

**Fig. 4:**
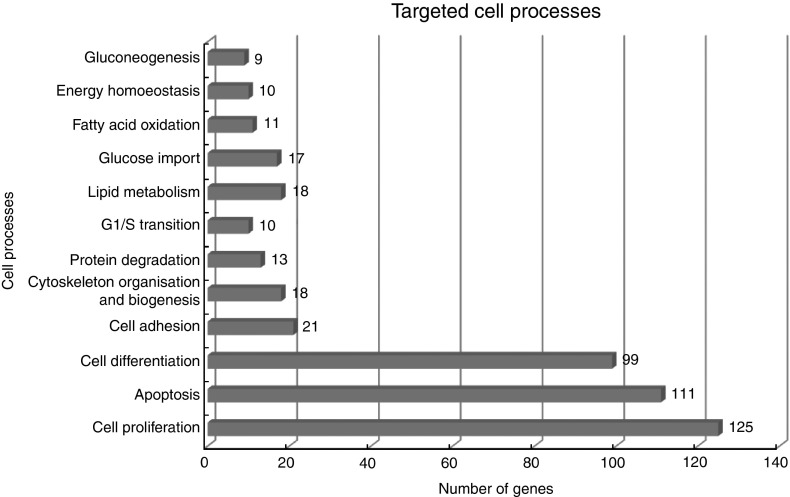
Major cell processes regulated by differentially expressed genes (DEG) between *β*-hydroxy-*β*-methylbutyrate and control cells. Analysis was performed using Pathway Studio Web Mammalian. Only relations with confidence levels ≥2 were included in the analysis. Details of all identified relationships between DEG and targeted cell processes are contained in the online Supplementary Material S3.

**Fig. 5:**
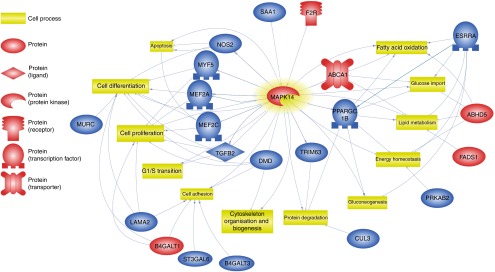
Relevance network over-viewing discussed relationships between *β*-hydroxy-*β*-methylbutyrate (HMB)-modulated genes and cell processes (Pathway Studio Web Mammalian). Genes are marked with red and blue colour for up- and down-regulation, respectively. F2R, coagulation factor II; SAA1, serum amyloid A1; NOS2, nitric oxide synthetase, inducible, 2; MEF2a and MEF2c, myocyte enhancer factor 2a and 2c; TGFB2, transforming growth factor, *β*2; DMD, dystrophin; Trim63, muscle-specific RING finger protein 1; ESRRA, oestrogen-related receptor α; ABHD5, abhydrolase domain-containing protein 5; PRKAB2, protein kinase, AMP-activated, β2 non-catalytic subunit; CUL3, cullin 3; LAMA2, laminins; MURC, muscle-related coiled-coil protein; MYF5, myogenic factor 5; ABCA1, ATP-binding cassette, sub-family A, member 1; PPARGC1B, peroxisome proliferator-activated receptor *γ*, coactivator 1 *β*; B4GALT1, *β*-1,4-galactosyltransferase 1; ST3GAL6, ST3 *β*-galactoside *α*-2,3-sialyltransferase 6; B4GALT3, *β*-1,4-galactosyltransferase 3; 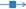, expression; 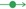, promoter binding; 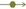, promoter modification; 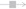, regulation.

Using Pathway Studio Web Mammalin Build Pathway Wizard Find Direct Links, we depicted all genes discussed in the present study that can directly or indirectly affect skeletal muscle cell functions (Fig. 3). Moreover, Pathway Studio Web Mammalian Build Pathway Wizard Find Common Targets algorithm allowed us to identify cell processes regulated by at least two of the DEG according to literature data. This resulted in fifty-six identified targets; among these, the twelve regulated by the highest number of genes were considered to be the most important for the HMB effect on ESC. A chart presenting these processes is presented in Fig. 4. From all targeted cell processes, we selected the most important relationships and are presented in Fig. 5. The online Supplementary Material S3 contains details of all identified relationships between DEG and cell processes.

## Discussion

The objective of the present study was to identify the molecular background of HMB action on equine skeletal muscle. In order to cover all the salient points of functional analysis, only relations significant in DAVID and possessing the highest reference number in Pathway Studio analysis were considered to be important. To date, no official genome nomenclature has been established for the horse. According to the guidelines published by The International Society for Animal Genetics, for all genes with human orthologues, official human gene symbols (Human Genome Organisation (HUGO) Gene Nomenclature Committee) are applied.

We decided to use a primary SC model because of its stem cell potential. SC are able to differentiate into multiple mesenchymal lineages^(^ ^24^ ^)^ and to self-renew^(^ ^25^ ^)^, because of which they maintain extraordinary regenerative properties of skeletal muscles. However, the capacity of SC to proliferate and differentiate may vary depending on the origin of the muscle^(^ ^26^ ^)^, cell surface markers expression^(^ ^27^ ^)^, myogenic regulatory factors (MRF) expression^(^ ^28^ ^)^ and muscle fibre type^(^ ^29^ ^)^. In our study, all samples of ESC were isolated from *semitendinosus* muscle, which in horses is composed mainly of type II fast-twitch fibre muscle^(^ ^30^ ^)^. SC originating from this type of muscle may have less adipogenic properties compared with SC from type I fibres^(^ ^29^ ^)^. Heterogeneity of the SC could limit *in vivo* significance of the data obtained in the present study.

In general, the present analysis underlined the role of HMB as a global regulator, which is shown by the strong over-representation of genes linked to the BP: ‘regulation of developmental process’ and ‘positive regulation of BP’. Moreover, functional analysis revealed significant enrichment in ontology terms associated with cellular responses (Table 3). The three main cellular processes include cell proliferation, apoptosis and differentiation, which suggest that HMB is an important cell growth regulator (Fig. 4 and 5).

In adult skeletal muscle, extracellular matrix proteins anchor SC between the basal lamina and the apical sarcolemma, which create a specialised micro-environment called a stem cell niche. It is able to produce factors controlling stem cell behaviour^(^ ^31^ ^)^. Impaired adhesion of SC to their niche can stimulate proliferation^(^ ^32^ ^)^. Thereby, enrichment of the terms ‘regulation of cell adhesion’ and ‘cellular localisation’ may suggest HMB’s ability to indirectly control ESC proliferation by affecting their localisation in the niche.

### Muscle development

The term ‘muscle organ development’ is the most significantly enriched annotation among genes regulated in ESC exposed to HMB (Table 3). This indicates that at least at the mRNA level HMB may affect muscle development (summarised on Fig. 3). A total of fourteen DEG were annotated to this term; however, among them, *Mapk14* (mitogen-activated protein kinase 14) possessed the highest potential to regulate other genes and cell processes (Fig. 3 and 5). *Mapk14* is activated by extracellular stimuli such as pro-inflammatory cytokines or physical stress, leading to direct activation of multiple cellular processes such as proliferation, differentiation, apoptosis and transcription regulation^(^ ^33^ ^)^. In SC, phosphorylation of MAPK14 may induce initiation^(^ ^34^ ^,^ ^35^ ^)^ or withdrawal^(^ ^36^ ^)^ from the cell cycle. The second can lead either to terminal differentiation or to programmed cell death^(^ ^37^ ^)^ depending on the nature of the stimulant and cell type. *In vitro* studies suggest that the two isoforms of *Mapk14*, p38*α* and p38*β*, appear to have different effects on cardiomyocyte hypertrophy: p38*β* seems to be more potent in inducing hypertrophy, whereas p38*α* appears to be more important in apoptosis^(^ ^38^ ^)^. The contribution of *Mapk14* in cellular responses to HMB has already been reported by Kornasio *et al*.^(^ ^8^ ^)^, who suggested that the MAPK/ERK pathways mediate HMB’s effects on myoblast proliferation. HMB-related increase in phosphorylation of MAPK14 was also observed in dexamethasone-induced muscle atrophy in rats^(^ ^39^ ^)^.

Except for its ability to influence multiple cell processes, *Mapk14* was reported to regulate many other genes from the analysis. One of them is *Nos2* (nitric oxid synthase 2, inducible), interesting because of its lowest expression among all genes. *Nos2* gene expression may be activated by *Mapk14*; however, it is assigned to shock signalling in inflammatory cells^(^ ^40^ ^)^ and its biological meaning in ESC remains unclear. Down-regulation of this gene by HMB has already been presented by Mitsutaka *et al*.^(^ ^41^ ^)^ in lipopolysaccharide-treated murine macrophages. This considered together may suggest an anti-inflammatory component of HMB action. *Mapk14-*dependent phosphorylation of transcription factors *Mef2a* and *Mef2c* (myocyte enhancer factor 2a and 2c) has been implicated in stress activation of immune, skeletal and cardiac muscle cells^(^ ^42^ ^,^ ^43^ ^)^. Among genes identified in our study, *Mapk14* posseses two upstream promoters, *Saa1* (serum amyloid A1) and *F2r* (coagulation factor II, thrombin receptor-like 2); however, so far, only the second gene has been implicated in striated muscle tissue development^(^ ^44^ ^,^ ^45^ ^)^, which means that *F2r* may link HMB and *Mapk14* (Fig. 3 and 5).

Another gene of particular importance to the ‘muscle organ development’ term is *Myf5*, belonging to the MRF family of transcription regulators^(^ ^46^ ^)^. The high expression of *Myf5* in adult skeletal muscle features committed SC and decreases when differentiation to myotubes occurs^(^ ^46^ ^,^ ^47^ ^)^. Accordingly, decreased expression levels of *Myf5* in ESC at the beginning of differentiation may indicate that HMB enhanced withdrawal of equine myoblasts from the cell cycle, compared with control cells. This finding is accompanied by previous reports presenting an HMB-dependent increase in mRNA and protein levels of muscle differentiation markers such as MyoD and myogenin^(^ ^8^ ^,^ ^16^ ^)^. However, at the time of our analysis, none of the differentiation markers reached significance criteria in ESC, which may emphasise the need for time-course studies in the future. Another down-regulated gene in HMB-treated cells was *Tgf-β2*. Activity of *Tgf-β2* has been recently linked with increased proliferation and delayed differentiation in C2C12^(^ ^48^ ^)^; thus, its down-regulation may confirm HMB-mediated enhancement of differentiation in ESC.

Other ‘muscle organ development’ annotated genes such as *Dmd* (dystrophin), *Lama2* and *Lama5* (laminins) encode protein complexes located in muscle sarcolemma and the basal lamina, respectively, protecting sarcolemma from mechanical damage during muscle contraction^(^ ^49^ ^,^ ^50^ ^)^ and, as described above, contribute to SC anchor in their niche^(^ ^31^ ^)^. This could be linked to HMB’s ability to decrease post-exercise muscle cell damage *in vivo* ^(^ ^13^ ^,^ ^14^ ^)^; however, in cultured ESC, its expression was decreased. The remaining genes annotated to the ‘muscle organ development’ term by DAVID include the following: *Zfp91* (zinc finger protein 91 homolog), acting as an activator of the non-canonical NF-*κ*B pathway^(^ ^33^ ^)^; *Itgb1bp2* (integrin *β*-1-binding protein 2, melusin 2)^(^ ^33^ ^)^; *Svil* (supervilin), involved in myosin II assembly, cell migration and focal adhesions^(^ ^33^ ^)^; *Murc* (muscle-related coiled-coil protein) controlling myofibrillar organisation^(^ ^33^ ^)^; and *Tagln3* (actin cross-linking/gelling protein) involved in contractile properties and early cell differentiation^(^ ^33^ ^)^.

### Muscle protein metabolism

One of the first described mechanisms of HMB action was the effect on muscle protein metabolism. Preliminary studies suggest that HMB protects the skeletal muscle by inhibiting protein degradation^(^ ^5^ ^)^ and by stimulating protein synthesis^(^ ^6^ ^)^; however, this issue is subjected to constant research^(^ ^17^ ^)^. Functional analyses have demonstrated significant DEG enrichment of terms associated with cellular protein maintenance (Table 3, Fig. 4). The three most important genes of this group are *Cul3* (cullin 3), *Trim63* and *Mapk14* (Fig. 5). *Cul3* is a scaffold protein of E3 ubiquitin-protein ligase complexes, which mediate the ubiquitination and subsequent proteasomal degradation of target proteins. Cul3 also interacts with Kelch family proteins, and disturbances in functioning of this complex are implicated in muscle myopathies^(^ ^51^ ^)^. E3 Ubiquitin ligase produced by *Trim63* regulates the proteasomal degradation of muscle proteins and inhibits *de novo* skeletal muscle protein synthesis under amino acid starvation, consequently leading to muscle atrophy^(^ ^52^ ^)^. As observed in the present study, down-expression of *Trim63* mediated by HMB confirms the results obtained by Aversa *et al*.^(^ ^39^ ^)^ in a dexamethasone-induced muscle atrophy model; however, in two most recent studies, the authors failed to demonstrate a similar effect on *Trim63* expression upon fasting in human and pig muscles^(^ ^17^ ^,^ ^53^ ^)^. This indicates that the effect of HMB on this gene expression could be species and/or condition related. Multiple studies suggest that *Mapk14* signalling may be involved in HMB-mediated stimulation of protein synthesis in catabolic conditions^(^ ^8^ ^,^ ^39^ ^,^ ^54^ ^)^, which may be confirmed by the up-regulation of this gene in HMB-treated ESC.

### Lipid metabolism and energy homoeostasis

Recent studies have revealed that HMB supplementation may alter metabolism, as evidenced by improved aerobic performance and increased fat loss during exercise^(^ ^11^ ^,^ ^12^ ^)^. This is confirmed in our study, which showed influence of DEG on cell processes such as ‘energy homoeostasis’, ‘lipid metabolism’, ‘glucose import’, ‘fatty acid oxidation’ and ‘gluconeogenesis’ (Fig. 4 and 5). An extensive amount of research describing the positive role of *Mapk14* on glucose uptake^(^ ^55^ ^)^ and gluconeogenesis^(^ ^56^ ^)^ has been published. Thereby, we postulate that apart from the established role of *Mapk14* in HMB-dependent influence on protein metabolism and cell growth it can mediate HMB influence on energy homoeostasis as well. The rate of post-exercise muscle glycogen synthesis is 2–3-fold slower in horses compared with other mammals^(^ ^1^ ^)^; therefore, the positive impact of HMB on glucose uptake could enhance this process in equine skeletal muscles. This is an interesting aspect of our study, which deserves more attention in future investigations. Another salient point of HMB influence on metabolism may be the transcription factor *Esrra* (oestrogen-related receptor *α*), controlling vast gene networks involved in all aspects of energy homoeostasis, including lipid and glucose metabolism as well as mitochondrial biogenesis and function^(^ ^57^ ^)^. Common targets algorithm showed its strong association with ‘fatty acid oxidation’ and ‘lipid metabolism’ (Fig. 5). *Essra* is targeted by *Ppargc1b* (peroxisome proliferator-activated receptor γ, coactivator 1 β) (PPAR-*γ* coactivator), a well-established regulator of *β*-oxidation of fatty acids and oxidative phosphorylation in mitochondria, which is highly induced during myogenic differentiation^(^ ^58^ ^)^. *Prkab*2 (protein kinase, AMP-activated, *β*2 non-catalytic subunit) is essential for the regulation of a multitude of metabolic processes maintaining energy homoeostasis, especially in tissues with high metabolic rates, such as skeletal muscle^(^ ^59^ ^)^. Bruckbauer *et al*.^(^ ^12^ ^)^ reported that HMB increases the activity of *Prkab*2 in adipocytes and muscle cells; however, our results showed that HMB slightly decreased its expression in ESC at the time of the analysis. *Prkab*2 senses cellular energy levels. In response to low cellular ATP levels, *Prkab*2 switches off ATP-consuming anabolic pathways (mechanistic target of rapamycin (mTOR) kinase pathway), which results in inhibition of cell growth, proliferation and macromolecules synthesis, and at the same time *Prkab*2 switches on catabolic pathways that generate ATP (e.g. glucose uptake, glycolysis, fatty acid oxidation)^(^ ^59^ ^)^.

In regulation of the cellular process ‘lipid metabolism’, two genes appear to take the lead – *Abca1* (ATP-binding cassette, sub-family A, member 1), encoding a membrane-associated protein belonging to the ATP-binding cassette transporters superfamily and *Abhd5* (abhydrolase domain-containing protein 5). The analysis indicated up-regulation of both in ESC. The latter encodes a co-activator of adipose triglyceride lipase, thereby enhancing adipocyte and muscle lipolysis^(^ ^60^ ^)^. *Abca1* is a key regulator of the reverse cholesterol transport process and HDL biogenesis. Increased *Abca1* expression was demonstrated in skeletal and cardiac muscles in response to training^(^ ^61^ ^)^, which indicates the role of *Abca1* in the reduction of CVD risk by physical exercise.

Several reports have established HMB’s role in supporting muscle cell membrane integrity during exercise^(^ ^13^ ^,^ ^14^ ^)^. However, as already mentioned, our analysis showed that at least at mRNA levels HMB decreased the expressions of genes encoding sarcolemmal scaffold proteins (*Dmd*, *Lama2*, *Lama5*). Alternatively, functional analysis enrichment of terms associated with lipid maintenance, as well as KEGG pathways ‘biosynthesis of unsaturated fatty acids’ and ‘glicosphingolipids biosynthesis’, may indicate HMB’s ability to support cell membrane integrity by decreasing its rigidity^(^ ^62^ ^)^. Moreover, this may have an indirect impact on the inflammatory processes, signal transduction and myoblast differentiation^(^ ^62^ ^,^ ^63^ ^)^ (Fig. 3).

### Conclusions

The results presented in this study suggest the capability of HMB to influence ESC proliferation, differentiation and apoptosis as well as inflammatory response, protein anabolism, sarcolemma integrity, and cell energy utilisation and storage. As we have summarised in Fig. 5, most of the above-mentioned processes could be controlled by the Mapk14 gene, which suggests that at least at the mRNA level HMB triggers its cellular responses by stress signalling pathways. It should be noted that *in vivo* response of ESC to HMB may differ from the presented results because of the heterogeneity of the SC population and undefined postprandial HMB concentrations in equine skeletal muscle. Moreover, transcription is only one step in the regulatory pathway that leads to functional protein synthesis, therefore, further research on the proteomic, biochemical and pharmacodynamic level is highly recommended.

In conclusion, this study demonstrated for the first time that HMB has the potential to influence ESC by controlling its global gene expression. Transcriptomic profile analysis identified valuable gene targets of HMB in ESC, which may support the role of HMB in improving skeletal muscle growth and regeneration in horses; however, the overall role of HMB in equine skeletal muscle remains equivocal and requires further research.

## Supplementary material

For supplementary material accompanying this paper visit http://dx.doi.org/10.1017/S000711451600324X. click here to view supplementary material
