# Supplementary material for: Characterisation of equine satellite cell transcriptomic profile response to β-hydroxy-β-methylbutyrate (HMB)
Source: Br J Nutr. 2016 Oct 3;116(8):1315–25. doi: 10.1017/S000711451600324X (PMC5082287; doi:10.1017/S000711451600324X)
Supplement: Supplementary file 1 [file S000711451600324Xsup.zip › S000711451600324Xsup001/S000711451600324Xsup001.pdf]

Supplementary material 1

No.	ProbeName	p (Corr)	p	Regulation	FC (abs)	FC	Log FC	GeneSymbol	Description	Sequence
1	A_69_P030882	0,05	0,00	down	1,80	-1,80	-0,85	TAGLN3	transgelin 3 [Source:HGNC Symbol;Acc:29868] [ENSECAT00000010210]	GTTGGATTATTTATTTATTTATTTGCCACAAATGTCCCTCCTCAACTTATAGAACGCACC
2	A_69_P104046	0,05	0,00	down	1,20	-1,20	-0,26	REST	RE1-silencing transcription factor [Source:HGNC Symbol;Acc:9966] [ENSECAT00000024094]	TGTTCGAACTCACACAGGAGAACGCCCATATAAATGTGAACTTTGTCCTTATTCAAGTTC
3	A_69_P095856	0,05	0,00	up	1,17	1,17	0,23	NDST2	Equus caballus N-deacetylase/N-sulfotransferase (heparan glucosaminyl) 2 (NDST2), transcript variant X1, mRNA [ENSECAT00000019975]	AATCCAATGATTTCCCTCTTCATCCCCAGCAGTGTATGTATTTATACCTGACTATGGGAG
4	A_69_P113376	0,04	0,00	down	1,28	-1,28	-0,36	SLC17A5	PREDICTED: Equus caballus solute carrier family 17 (acidic sugar transporter), member 5 (SLC17A5), mRNA XM_001498010.3	GCATCTTAAGTGGTTTTCAAGCACACTTTGAAGGCATTTATGTGATTTAGAACTTGGTTT
5	A_69_P117885	0,03	0,00	down	6,21	-6,21	-2,63	DQB	PREDICTED: Equus caballus MHC class II DQ-beta chain (DQB), mRNA	AAGCCCATTGAAGCCATTTACCTGACCCTAGAGCTTTTATCATAATTAAACATGATTATG
6	A_69_P107556	0,04	0,00	down	2,53	-2,53	-1,34	PEG3	Select seq ref|XM_014730920.1| PREDICTED: Equus caballus paternally expressed 3 (PEG3), transcript variant X9, mRNA	TAGAAACTTTGAAGTTGCAGAAGCATAGAAGAGAAACTCGCCCATATTTTCATCATCCAG
7	A_69_P050501	0,04	0,00	up	1,13	1,13	0,18	FARSB	phenylalanyl-tRNA synthetase, beta subunit [Source:HGNC Symbol;Acc:17800] [ENSECAT00000025155]	GGAACATCCATTTGTGCTTTAATGTTTAATAAAGGAGAAACACTGTCTGGCTCTGTGGGT

8	A_69_P103296	0,04	0,00	up	1,14	1,14	0,19	FOSL2	PREDICTED: Equus caballus FOS-like antigen 2 (FOSL2), transcript variant X3, mRNA [XM_005600107]	AAGAGAACACAACGAAAGATCCTACACTGTTCCTCAAAAACTATTTTCTCTGTGTGATTA
9	A_69_P071344	0,04	0,00	up	1,26	1,26	0,34	ANAPC15	anaphase promoting complex subunit 15 [Source:HGNC Symbol;Acc:24531] [ENSECAT00000022428]	ATGAGATGAATGACTACAATGAGTCACCTGATGATGGAGAGGTCAATGAGGTGGACATGG
10	A_69_P108817	0,04	0,00	down	1,93	-1,93	-0,95	SUSD5	PREDICTED: Equus caballus sushi domain containing 5 (SUSD5), mRNA	AATGTTTAATATTCATGAAAATTGGGTGGGGGGATCCATTAACACAAAGCTTCTGAGATA
11	A_69_P121564	0,04	0,00	up	1,33	1,33	0,42			ACACCGTATAATGCTATGGTTTCCCCTCTTGCTGGAAAGATTCTTCTTGAAAACCAGACT
12	A_69_P122693	0,04	0,00	down	1,73	-1,73	-0,79			CTTTCCATCTCTCAGACCCTGGCGCTTTCTTAATCTCTATCCAATAAAATGAAACCAAAA
13	A_69_P124671	0,05	0,00	up	1,19	1,19	0,25	PKIG	protein kinase (cAMP-dependent, catalytic) inhibitor gamma [Source:HGNC Symbol;Acc:9019] [ENSECAT00000007863]	ATGCAACAGGCATGATGGAGGTCGAGTCCTCCTACTCGGACTTCATCTCCTGTGACCGGA
14	A_69_P123391	0,05	0,00	down	1,92	-1,92	-0,94		TSA: Equus caballus contig26079.EqcaPBMC mRNA sequence. [JL640016]	GTATGTATTTACTTCAGGTTGACTTGTGTCCTTATAAACTCTTACTCGGATGATTTGAAC
15	A_69_P041061	0,04	0,00	down	1,10	-1,10	-0,13	ARGLU1	PREDICTED: Equus caballus arginine and glutamate rich 1 (ARGLU1), mRNA	CCACTGACTTCAGAGTTATACTCTGTTTATTACATCATAATGCTGGTTCTGCTGACTTTT
16	A_69_P012236	0,05	0,00	down	1,53	-1,53	-0,61	PLXDC2	plexin domain containing 2 [Source:HGNC Symbol;Acc:21013] [ENSECAT00000018697]	TATGCTGAGGTTGAACCAGTGGGAGAGAAAGAAGGTTTCATTGTATCAGAGCAATGCTAA

17	A_69_P077187	0,03	0,00	down	1,88	-1,88	-0,91	GBP6	guanylate binding protein family, member 6 [Source:HGNC Symbol;Acc:25395] [ENSECAT00000013954]	GATACTCCCTGGGTTGCAAAAATCTTGGACAGATTTGGCAATGAGGTCACTTCAGTATTA
18	A_69_P034276	0,04	0,00	up	1,54	1,54	0,63	CA4	carbonic anhydrase IV [Source:HGNC Symbol;Acc:1375] [ENSECAT00000024391]	ATTCTCCCAGAAGCTGTACTACGACAATGAGCAGAAACTGAGTATGACGGAAAACGTCAG
19	A_69_P055531	0,04	0,00	down	3,01	-3,01	-1,59	LRTM1	leucine-rich repeats and transmembrane domains 1 [Source:HGNC Symbol;Acc:25023] [ENSECAT00000011435]	ATTTTAGGCCAGAAGAAAGTGTCTGAGTAGGGCTAGTGTGTTCACTGTTACTGCCTCAAT
20	A_69_P102122	0,04	0,00	down	2,16	-2,16	-1,11	CYTIP	PREDICTED: Equus asinus cytohesin 1 interacting protein (CYTIP), transcript variant X1, mRNA	CCATTAAAGTTCATGATTTCTCTGTTCTGGTGATACAGCTGCTCTTTGCCTATATTCTAG
21	A_69_P038318	0,04	0,00	down	1,22	-1,22	-0,28	DRB	Equus caballus MHC class II DR-beta chain (DRB), mRNA [NM_001142811]	ATGCACCTGAACTCACCTCTGCCCACATTTCTTTATAAAGTTTTTCTCAAATAAACATGG
22	A_69_P086096	0,04	0,00	down	1,50	-1,50	-0,59	COL8A2	collagen, type VIII, alpha 2 [Source:HGNC Symbol;Acc:2216] [ENSECAT00000013270]	TGAGTACATCCACTCCTCCTTTTCAGGATTCTTGCTCTGCCCCACATAACCCGCGGGGGA
23	A_69_P017336	0,04	0,00	up	1,31	1,31	0,39	KDELR2	KDEL (Lys-Asp-Glu-Leu) endoplasmic reticulum protein retention receptor 2 [Source:HGNC Symbol;Acc:6305] [ENSECAT00000006623]	CGTGTCTACAATGAAATTGTACTCCTTGACGGCTGGTAGATTATATATTCTTCCATCTAT

24	A_69_P065073	0,04	0,00	up	1,38	1,38	0,46	POP5	processing of precursor 5, ribonuclease P/MRP subunit (S. cerevisiae) [Source:HGNC Symbol;Acc:17689] [ENSECAT00000010367]	ATGTCAGAAGTTCCTGATTCAGTACAACCGGAGACAGCTGTTGATCTTGTTGCAACACTG
25	A_69_P013386	0,05	0,00	down	1,52	-1,52	-0,61	TMEM38B	transmembrane protein 38B [Source:HGNC Symbol;Acc:25535] [ENSECAT00000014020]	CAAAGCCTACAGCTGATGCTTCAGATAAAATGAAAAAGAAACATGCTAAGAAGACTGAGT
26	A_69_P000971	0,04	0,00	up	1,34	1,34	0,42	C-SKI	Equus caballus SKI (C-SKI), mRNA [NM_001081818]	TGGACACCAAGGAAGCCAAAGAGAAGTTCCTGCACGAGGTGGTGAAGATGCGTGTGAAGC
27	A_69_P008071	0,05	0,00	down	1,35	-1,35	-0,43	GPR126	G protein-coupled receptor 126 [Source:HGNC Symbol;Acc:13841] [ENSECAT00000016130]	CAGACAACTTCTATAAAAATATTATCATGTCAGACTCCTTCAGCCACAGCACAAAGTTTT
28	A_69_P034666	0,05	0,00	up	1,17	1,17	0,22	TMEM97	transmembrane protein 97 [Source:HGNC Symbol;Acc:28106] [ENSECAT00000012907]	CTGCTTTTCATGTTGTGGAGCCCCCACTACAAGTATGAGGAGAAAAGAAAGAAAAAATGA
29	A_69_P119107	0,05	0,00	down	2,12	-2,12	-1,09	MYF5	myogenic factor 5 [Source:HGNC Symbol;Acc:7565] [ENSECAT00000021416]	ATGTGCTATGAACTAAAAATCTAGTCTAGATCAGTTCTGCCAGGAGGGCCTATTACACAG
30	A_69_P011041	0,03	0,00	up	1,10	1,10	0,14	DPF2	D4, zinc and double PHD fingers family 2 [Source:HGNC Symbol;Acc:9964] [ENSECAT00000003581]	ATGCCTGTGACATTTGTGGAAAACGTTATAAAAACCGACCAGGGCTCAGCTACCACTATG
31	A_69_P034866	0,05	0,00	down	1,54	-1,54	-0,62	SSH2	slingshot protein phosphatase 2 [Source:HGNC Symbol;Acc:30580] [ENSECAT00000015910]	AGACTGAAAAAAGCAAATGATAAAAAACGGACAACCAACCCCTTCTATAATACCATGTGA

32	A_69_P041901	0,04	0,00	down	1,37	-1,37	-0,46	TANC1	tetratricopeptide repeat, ankyrin repeat and coiled-coil containing 1 [Source:HGNC Symbol;Acc:29364] [ENSECAT00000019966]	AGCCCACAGGAGTCACCTTCCTTCAGCAAAACCAAAACGATCATTCATAGAGTCAAACGT
33	A_69_P038366	0,04	0,00	down	2,31	-2,31	-1,21	LOC100060996	PREDICTED: Equus caballus HLA class II histocompatibility antigen, DM beta chain (LOC100060996), transcript variant X1, mRNA	TCATTCCTGGGAACATTCTGTAACAAAGTTATTGTCCAAGCCTGTTTGTAGGGTGAATGT
34	A_69_P012239	0,04	0,00	down	1,51	-1,51	-0,60	PLXDC2	plexin domain containing 2 [Source:HGNC Symbol;Acc:21013] [ENSECAT00000018697]	TGCCTATGCTGAGGTTGAACCAGTGGGAGAGAAAGAAGGTTTCATTGTATCAGAGCAATG
35	A_69_P059191	0,05	0,00	down	1,35	-1,35	-0,43	AAK1	AP2 associated kinase 1 [Source:HGNC Symbol;Acc:19679] [ENSECAT00000006573]	GAAAAACTAATTGAGGGACTCAAATCTCCTGACACTTCTCTTCTGCTCCCTGACCTCTTG
36	A_69_P050086	0,04	0,00	down	1,17	-1,17	-0,23	REPS1	RALBP1 associated Eps domain containing 1 [Source:HGNC Symbol;Acc:15578] [ENSECAT00000017544]	CCGTTAACTGTGAACATGCCCATTTTTAAGCGGTGTTGAGTTCAAGTCAATTTGGAGACC
37	A_69_P085918	0,04	0,00	down	1,35	-1,35	-0,43	MACF1	microtubule-actin crosslinking factor 1 [Source:HGNC Symbol;Acc:13664] [ENSECAT00000017611]	GCGGTGCAGGGTTGTAAACCTGCTTTATCTTTTAGGATTATTCCTAAATGCATCTTCTTT
38	A_69_P058226	0,03	0,00	down	1,74	-1,74	-0,80	KCNIP3	Kv channel interacting protein 3, calsenilin [Source:HGNC Symbol;Acc:15523] [ENSECAT00000019724]	ACCTGTCAGAAGGATGAAAACATCATGAGCTCCATGCAGCTGTTCGAGAATGTCATCTAG
39	A_69_P091441	0,05	0,00	down	1,94	-1,94	-0,96	ITGB1BP2	integrin beta 1 binding protein (melusin) 2 [Source:HGNC Symbol;Acc:6154] [ENSECAT00000016364]	GAGGCTGAAGGAGGGGAGAACAAAAGTGTCCAAACTATGCTGTTTTTTCCCTTAAATAAA

40	A_69_P042716	0,05	0,00	down	1,21	-1,21	-0,28	NABP1	nucleic acid binding protein 1 [Source:HGNC Symbol;Acc:26232] [ENSECAT00000017708]	AATCAAACAGTCATGACCACAATAAGTAATGGCAGGGACCCTCGGAGAGCCTTTAAAAGA
41	A_69_P001166	0,05	0,00	down	1,68	-1,68	-0,75	ANG	Equus caballus angiogenin, ribonuclease, RNase A family, 5 (ANG), mRNA [NM_001081899]	AGAAGCATTGTCATTGCCTGTGAAAACGGCTTGCCTGTCCACTTTGATGAGTCCTTTTTC
42	A_69_P085917	0,04	0,00	down	1,33	-1,33	-0,41	MACF1	microtubule-actin crosslinking factor 1 [Source:HGNC Symbol;Acc:13664] [ENSECAT00000017611]	CGGTGCAGGGTTGTAAACCTGCTTTATCTTTTAGGATTATTCCTAAATGCATCTTCTTTA
43	A_69_P023981	0,05	0,00	down	1,24	-1,24	-0,31	NKX2-2	NK2 homeobox 2 [Source:HGNC Symbol;Acc:7835] [ENSECAT00000020059]	AGTCTCTGCAGCACATGCAGTACAACGCCCAGTACAGCTCGGCCAGCACCCCCCAGTATC
44	A_69_P086176	0,05	0,00	up	1,39	1,39	0,48	ZMYM6NB	ZMYM6 neighbor [Source:HGNC Symbol;Acc:40021] [ENSECAT00000019530]	TAACTTGCTTTTGACCCTGCTCATGATGGGGGCTCTCTTCACTTTGGTATCTCTGAAAGA
45	A_69_P005416	0,03	0,00	up	1,22	1,22	0,28	ARG2	arginase 2 [Source:HGNC Symbol;Acc:664] [ENSECAT00000017770]	AAATACACAATACAGGGTTGCTGTCAGCACTGGATCTTGTTGAAGTCAATCCTTGGGTGG
46	A_69_P106902	0,04	0,00	down	1,86	-1,86	-0,89	TGFB2	Select seq ref|XM_005608026.2| PREDICTED: Equus caballus transforming growth factor beta 2 (TGFB2), transcript variant X3, mRNA	CAAGCAAGTCTGCATTGAGATAAAGACCCTAAAAACACATGCTTTGTACCAGCTGCCTAA
47	A_69_P013540	0,04	0,00	down	1,65	-1,65	-0,73	KIAA1958	KIAA1958 [Source:HGNC Symbol;Acc:23427] [ENSECAT00000008002]	ACTTAGCCAAGAAGGTCAAGCTGGAAAACTGTGAGAACTTCACCTTCGTCTCGTTTACTC

48	A_69_P114566	0,04	0,00	up	1,23	1,23	0,29	FAM134A	PREDICTED: Equus caballus family with sequence similarity 134 member A (FAM134A), partial mRNA	CAGTGTGATATGTTTAGTGAGACTTAGCATGTGTGAATAAAGTGTATGCAGGAGGAAAAA
49	A_69_P114667	0,05	0,00	down	1,69	-1,69	-0,76	CPE	PREDICTED: Equus przewalskii carboxypeptidase E (CPE), mRNA	TCTGATTCTATGGCTCTTATGCATCAGTTGTGGACATGGACTTTAAATAAATATACACAC
50	A_69_P067026	0,04	0,00	up	1,36	1,36	0,45	SERPINB8	serpin peptidase inhibitor, clade B (ovalbumin), member 8 [Source:HGNC Symbol;Acc:8952] [ENSECAT00000011218]	TTCATCAGGCATCTCAAAACCAATAGCATTTTGTTCTGTGGCAGGTTCTCTTCTCCATAA
51	A_69_P037991	0,05	0,00	down	2,27	-2,27	-1,18	LOC100053918	Select seq ref|XM_014734669.1| PREDICTED: Equus caballus HLA class I histocompatibility antigen, B-46 alpha chain-like (LOC100053918), transcript variant X1, mRNA	TTGTGATGTGGATGAAGAAGTGCTCAGGTGGAAAAGGAACAAGCTATGTTGTGGCTGCAA
52	A_69_P041495	0,05	0,00	up	1,54	1,54	0,63	STEAP3	STEAP family member 3, metalloreductase [Source:HGNC Symbol;Acc:24592] [ENSECAT00000006653]	AGAATGACACACACGTGCGTGTGATGTATATGTTCCTATATATTTCATATGTATAACAGG
53	A_69_P090516	0,03	0,00	down	2,00	-2,00	-1,00	DMD	PREDICTED: Equus caballus dystrophin (DMD), mRNA	GCCATGGAGTCCTTAGTTTCAGTCATGACAGATGAAGAAGGGGCAGAATAAATGTTTTAC
54	A_69_P116470	0,04	0,00	down	1,21	-1,21	-0,28	BSDC1	PREDICTED: Equus caballus BSD domain containing 1 (BSDC1), transcript variant X1, mRNA [XM_001503784]	TTGTCCTGAACCTTTTCCAGACTTGCCGTTAACAGCATTAAAGAGATTCAACAGAAGCCG
55	A_69_P109091	0,05	0,00	up	1,31	1,31	0,39	RFXAP	PREDICTED: Equus przewalskii regulatory factor X-associated protein (RFXAP), partial mRNA	GTCAAGAGAGTATTTCCAATGTTAGGACAATAAATGCACGTGTCTGAAGTTTAATAATGC

56	A_69_P088046	0,03	0,00	up	1,42	1,42	0,51	PUSL1	pseudouridylate synthase-like 1 [Source:HGNC Symbol;Acc:26914] [ENSECAT00000006377]	TTCGAGAGCCAGTCATTCCTGTACAGACAGGTACGGAGGATGACGGCTGTACTGGTGGCT
57	A_69_P021389	0,04	0,00	up	1,07	1,07	0,10	TOMM22	translocase of outer mitochondrial membrane 22 homolog (yeast) [Source:HGNC Symbol;Acc:18002] [ENSECAT00000010721]	TGAATTATTCTCTTTCACTCTGATACCAGAGTGCAGCAATGCAGACAGTTACTCCAGCTC
58	A_69_P086931	0,05	0,00	down	1,70	-1,70	-0,77	MYOM3	PREDICTED: Equus caballus myomesin 3 (MYOM3), mRNA [ENSECAT00000024630]	AGCAAGGTCAAGTACTACAACGTGGAGTACATGAAGACTGCCTGGTTCCACAAAGAGAAA
59	A_69_P081776	0,04	0,00	down	1,22	-1,22	-0,29	NFATC3	nuclear factor of activated T-cells, cytoplasmic, calcineurin-dependent 3 [Source:HGNC Symbol;Acc:7777] [ENSECAT00000014815]	GTTGCTTAGCTAGGATTGGTGATTTGTACTGCTTTATGGTCATCTGAAGGACCCTTTAGC
60	A_69_P013493	0,04	0,00	down	1,20	-1,20	-0,26	KIAA0368	KIAA0368 [Source:HGNC Symbol;Acc:29020] [ENSECAT00000022800]	AAATTAGAAGGGGAAGAAACAAACAAGTGCCATGTTCATTGGGGGTTGAAATGGTGGTAT
61	A_69_P075876	0,05	0,00	up	2,01	2,01	1,01	GABPB2	GA binding protein transcription factor, beta subunit 2 [Source:HGNC Symbol;Acc:28441] [ENSECAT00000015504]	TAACTGTACCTGCTGGTCAGGTTGCAGAGGAGACTGTAATTGAAGAAGAAGAGGAAGAAG
62	A_69_P000869	0,04	0,00	down	2,39	-2,39	-1,26	INOS	Equus caballus inducible nitric oxide synthase (INOS), mRNA [NM_001081769]	AGGCACTTGGGTCTTCCTTGTATGTCTCCTTGATGGAGATATTTATATGAAATGCATTTT
63	A_69_P042741	0,04	0,00	down	1,62	-1,62	-0,70	STK17B	Serine/threonine-protein kinase 17B-like protein; Uncharacterized protein [Source:UniProtKB/TrEMBL;Acc:F7D434] [ENSECAT00000000416]	GCTGCTAGTAGAATTTTATTTCCTATCCTGAGATATCAAGTCTGCAGAGAAGAAGATATT

64	A_69_P069336	0,04	0,00	up	1,17	1,17	0,23	ROBO3	roundabout, axon guidance receptor, homolog 3 (Drosophila) [Source:HGNC Symbol;Acc:13433] [ENSECAT00000014933]	AGAGCAGCAGGCAGCATGTCCTCCTTGGAACGGGAGCGCAGTGGGGAGAGAAGAATGGTG
65	A_69_P109101	0,04	0,00	down	1,27	-1,27	-0,34	SAMD4A	PREDICTED: Equus caballus sterile alpha motif domain containing 4A (SAMD4A), transcript variant X6, mRNA	CCTCCCATGTAACTAAATCATGGCTATATTTCATATCAACGTTATATTGAAAGTGAAGGG
66	A_69_P117286	0,04	0,00	down	1,93	-1,93	-0,95	CPE	PREDICTED: Equus caballus carboxypeptidase E (CPE), mRNA [XM_001915786]	TTAGGGTTTTCTCTTGGTTGTAGATTGGCCCAGATTTGCATTCTGAATGAATAAAAGTAA
67	A_69_P021751	0,03	0,00	up	1,35	1,35	0,43	TSPO	translocator protein (18kDa) [Source:HGNC Symbol;Acc:1158] [ENSECAT00000023154]	ATTGGGCAGCTGGCTCTGAACTGGGCATGGCCGCCCATCTTCTTTGGCGCCCGACAAATG
68	A_69_P122695	0,03	0,00	down	1,69	-1,69	-0,76	KCNK12		TGCTTTCCATCTCTCAGACCCTGGCGCTTTCTTAATCTCTATCCAATAAAATGAAACCAA
69	A_69_P019361	0,04	0,00	up	1,11	1,11	0,15	RSL1D1	ribosomal L1 domain containing 1 [Source:HGNC Symbol;Acc:24534] [ENSECAT00000027038]	AGAAAACGTGGAGATACAAAAGCATGCCACAGGAAAGAAGTCTCCAAAACAGAGTCCTGG
70	A_69_P069542	0,04	0,00	down	1,11	-1,11	-0,15	PRDM10	PR domain containing 10 [Source:HGNC Symbol;Acc:13995] [ENSECAT00000012308]	CATCATTACAACCACCACCAACGGGAACGGAAGCAGTGAAGTGCATATCACTAAACCTTA
71	A_69_P129027	0,04	0,00	down	1,51	-1,51	-0,59	STK17B	TSA: Equus caballus contig26573.EqcaPBMC mRNA sequence. [JL616416]	CTTGTGTTCAGTCCTACTGGGGGCAGATTCACTCCTTTTTGGGTAATCTTTGGTGACTTT
72	A_69_P052336	0,04	0,00	down	1,38	-1,38	-0,46	BCAT1	branched chain amino-acid transaminase 1, cytosolic [Source:HGNC Symbol;Acc:976] [ENSECAT00000022642]	AAATTGACTGATATTCAGTATGGAAGAGAAGAGAGCAACTGGACAATCATAGTATCCTGA

73	A_69_P075971	0,05	0,00	up	1,17	1,17	0,23	TARS2	threonyl-tRNA synthetase 2, mitochondrial (putative) [Source:HGNC Symbol;Acc:30740] [ENSECAT00000015021]	ATGCTGAAGAGATTTTCTGAGCTTCTGTATATAGATGTGACAGAGACCTGCAGGAGCCAC
74	A_69_P019206	0,05	0,00	down	1,70	-1,70	-0,76	SYT17	synaptotagmin XVII [Source:HGNC Symbol;Acc:24119] [ENSECAT00000009185]	TTTCTACAACGAGTCCTTCAGCTTCAAAGTTCCCCAAGAAGAACTGGAAAATGCCAGCCT
75	A_69_P008146	0,05	0,00	down	1,58	-1,58	-0,66	ZBTB18	zinc finger and BTB domain containing 18 [Source:HGNC Symbol;Acc:13030] [ENSECAT00000019934]	GAGGGGGGAATAACTTATAATGCTGTTTAGTTTTGTACTATTGGTGTGTTGGTGAATTTT
76	A_69_P066438	0,04	0,00	down	2,06	-2,06	-1,04	GAREM	GRB2 associated, regulator of MAPK1 [Source:HGNC Symbol;Acc:26136] [ENSECAT00000012124]	GAAGAAAATCATGCAATTCATTAATGGCTGGAGGCCCAAAATATAGCCAAATGACCCCAG
77	A_69_P118776	0,03	0,00	down	3,90	-3,90	-1,96	MAL	PREDICTED: Equus caballus myelin and lymphocyte protein-like (LOC102147820), mRNA [XM_005600243]	TACATGGTCCATGCGGTGTTTTCTTTAATCAGATGGAAGAAAGCAGCAGAAGAACTTTAG
78	A_69_P106762	0,03	0,00	down	1,24	-1,24	-0,31	IMPAD1	PREDICTED: Equus caballus inositol monophosphatase domain containing 1 (IMPAD1), mRNA	TGCATTCTTTACTTCGGATGCTACCCCATGGCCAATTAGCTCTAGAAAATGTCATTGCCT
79	A_69_P055622	0,04	0,00	up	1,26	1,26	0,33	GNL3	guanine nucleotide binding protein-like 3 (nucleolar) [Source:HGNC Symbol;Acc:29931] [ENSECAT00000015819]	GAACCTTGTCCTTTTGTAAGAGTTTTTGTTTTTAATGTTTTGAGGAGACTCCTAAGCTGT
80	A_69_P061854	0,05	0,00	up	1,24	1,24	0,31	RPS14	ribosomal protein S14 [Source:HGNC Symbol;Acc:10387] [ENSECAT00000026184]	CGTGGTCGCCGTCTGTGAACAGGACTCCTCAAATTGTTTTCTGTTAATAAATTGTCTTCA

81	A_69_P102193	0,03	0,00	up	5,09	5,09	2,35		APL1_9_G09.g1_A008 Liver (APL1) Equus caballus cDNA, mRNA sequence [BM780851]	CACAACTTGCCTGTTTCTATGAGATCTGTGAATTCCAAATAAACGTGTGTTGACTCTAAA
82	A_69_P052156	0,05	0,00	down	1,57	-1,57	-0,65	MGP	matrix Gla protein [Source:HGNC Symbol;Acc:7060] [ENSECAT00000011027]	GTGAAGGTTGAAGGGGAGTTAAGATGTGTAATTACTACACAATAAACTTCTGGTTGGATC
83	A_69_P012696	0,04	0,00	down	1,17	-1,17	-0,22	SPCS1	PREDICTED: Equus asinus signal peptidase complex subunit 1 (SPCS1), partial mRNA	TTGCACCTGCTTTTGTTTCGTATGAGATGAGCTAAATTTCACACCCAAAACATGGGCTAA
84	A_69_P081269	0,05	0,00	up	7,28	7,28	2,86	LOC100630858	Metallothionein [Source:UniProtKB/TrEMBL;Acc:F6VKN2] [ENSECAT00000001640]	ACCCCTGACTTGATTGCTACATTCCTTTTTCTATGAAATGTTTGAGTGGCAATAAACTCA
85	A_69_P000866	0,04	0,00	down	2,39	-2,39	-1,25	INOS	Equus caballus inducible nitric oxide synthase (INOS), mRNA [NM_001081769]	CACTTGGGTCTTCCTTGTATGTCTCCTTGATGGAGATATTTATATGAAATGCATTTTGTT
86	A_69_P095967	0,04	0,00	up	1,29	1,29	0,37	PLAC9	placenta-specific 9 [Source:HGNC Symbol;Acc:19255] [ENSECAT00000011851]	CAGAGCCGTGCATTCTGGCCTTCCTTCCTCAGCTTTGTACACAATAAAAGCTACTTCTTT
87	A_69_P096536	0,04	0,00	up	1,34	1,34	0,43	GDF10	growth differentiation factor 10 [Source:HGNC Symbol;Acc:4215] [ENSECAT00000009917]	GTCTTTCATTGCTAGTGACTTAGCCCCTCAGTGCTGGTGTGAACCCGCGAAGGAATCTGG
88	A_69_P120601	0,04	0,00	down	1,43	-1,43	-0,52		TSA: Equus caballus contig07897.EqcaPBMC mRNA sequence. [JL623434]	GTCCAGTAATTTCAATCTGATCCTTACTTTGTCTTCATTGTGCTTATGCTGAATACTCCT
89	A_69_P078174	0,05	0,00	up	1,10	1,10	0,14	MRPS24	mitochondrial ribosomal protein S24 [Source:HGNC Symbol;Acc:14510] [ENSECAT00000015296]	AATAAATAGACTCCATGTGTCTTGCCTCTCAGTCTGTCTTTCCTCTTTAGGTGAGGGAAC

90	A_69_P113296	0,04	0,00	down	1,76	-1,76	-0,82	SEMA4D	PREDICTED: Equus caballus sema domain, immunoglobulin domain (Ig), transmembrane domain (TM) and short cytoplasmic domain, (semaphorin) 4D (SEMA4D), transcript variant X4, mRNA	CTCGTCTTCCAGCATGATCAGATTCAAATGCTTTGGTGTAAACATTTGTGTTTTATAAGA
91	A_69_P095858	0,04	0,00	up	1,19	1,19	0,25	NDST2	Uncharacterized protein [Source:UniProtKB/TrEMBL;Acc:F7AV98] [ENSECAT00000019975]	TTAATCCAATGATTTCCCTCTTCATCCCCAGCAGTGTATGTATTTATACCTGACTATGGG
92	A_69_P112707	0,05	0,00	up	1,25	1,25	0,32	MFSD10	PREDICTED: Equus caballus major facilitator superfamily domain containing 10 (MFSD10), mRNA	TCCCAAGCCTGTGTCGTATATGCCACTAAACTTCATGTTGTAATACAAAGAGTTAAAATA
93	A_69_P117221	0,04	0,00	up	1,86	1,86	0,89	ZFP36L2	PREDICTED: Equus przewalskii ZFP36 ring finger protein-like 2 (ZFP36L2), mRNA	TCGGCAAAATGTGGAGTGTATGTTCTTTTCACAGTAATATATGCCTTTTGTAACTTCACT
94	A_69_P108957	0,05	0,00	up	1,20	1,20	0,27		CT02032A1A11 Equine Articular Cartilage cDNA Library Equus caballus cDNA clone CT02032A1A11, mRNA sequence [CX602199]	GGGCAGCTGCACTGGTTTACTTTAAAATGTACAGATCTTCCCATTAAATTCATGATAGAT
95	A_69_P102587	0,04	0,00	up	1,87	1,87	0,90	ABCA1	PREDICTED: Equus caballus ATP-binding cassette, sub-family A (ABC1), member 1 (ABCA1), mRNA [XM_001493790]	GAGATTCCTGCCTTTGCTTCTTTTGGACACTCTGGAAACCTTATTAACAACTGTGAATAT
96	A_69_P112463	0,05	0,00	up	1,34	1,34	0,42		HL01014B1G03 Equine lymphocyte cDNA Library Equus caballus cDNA clone HL01014B1G03, mRNA sequence [DN505256]	CTGAGCACTTGCCTTGTCTGTAGAGATAATAGTAGAACCTGTTTCATAAGACTGTTTTGT

97	A_69_P005420	0,04	0,00	up	1,22	1,22	0,28	ARG2	arginase 2 [Source:HGNC Symbol;Acc:664] [ENSECAT00000017770]	GAGGAAATACACAATACAGGGTTGCTGTCAGCACTGGATCTTGTTGAAGTCAATCCTTGG
98	A_69_P017337	0,05	0,00	up	1,28	1,28	0,35		KDEL (Lys-Asp-Glu-Leu) endoplasmic reticulum protein retention receptor 2 [Source:HGNC Symbol;Acc:6305] [ENSECAT00000006623]	CTTTGAAATTGTCGATTCTTCGTGTCTACAATGAAATTGTACTCCTTGACGGCTGGTAGA
99	A_69_P117881	0,03	0,00	down	6,59	-6,59	-2,72			CCATTGAAGCCATTTACCTGACCCTAGAGCTTTTATCATAATTAAACATGATTATGGGTT
100	A_69_P119108	0,05	0,00	down	2,09	-2,09	-1,06	MYF5	myogenic factor 5 [Source:HGNC Symbol;Acc:7565] [ENSECAT00000021416]	CATGTGCTATGAACTAAAAATCTAGTCTAGATCAGTTCTGCCAGGAGGGCCTATTACACA
101	A_69_P015701	0,05	0,00	up	1,22	1,22	0,28		Uncharacterized protein [Source:UniProtKB/TrEMBL;Acc:F7ALP2] [ENSECAT00000014068]	CTCTTACAGTCGCTGAGGCTCCTGCAGCTCCTTGCATCAGTTTCTTTGGTGAGTAAAATT
102	A_69_P021624	0,04	0,00	up	1,16	1,16	0,22	NHP2L1	NHP2 non-histone chromosome protein 2-like 1 (S. cerevisiae) [Source:HGNC Symbol;Acc:7819] [ENSECAT00000000315]	TGTGTCTCGTTTTGTCTGTTTTAGCATGTAGTATTTCCAGCTACCTTCTCCTGATATAAA
103	A_69_P081267	0,05	0,00	up	7,54	7,54	2,91	LOC100630858	Metallothionein [Source:UniProtKB/TrEMBL;Acc:F6VKN2] [ENSECAT00000001640]	CCCTGACTTGATTGCTACATTCCTTTTTCTATGAAATGTTTGAGTGGCAATAAACTCATT
104	A_69_P010501	0,04	0,00	up	1,33	1,33	0,41	FADS1	fatty acid desaturase 1 [Source:HGNC Symbol;Acc:3574] [ENSECAT00000025250]	TTCCTTTTTCTAGTTTGGCAGATGCAGTAGGTTGGTGAGCAAAGAGGAAGTCAGGAGAGT

105	A_69_P071343	0,04	0,00	up	1,23	1,23	0,30	ANAPC15	anaphase promoting complex subunit 15 [Source:HGNC Symbol;Acc:24531] [ENSECAT00000022428]	TGAGATGAATGACTACAATGAGTCACCTGATGATGGAGAGGTCAATGAGGTGGACATGGA
106	A_69_P037955	0,04	0,00	down	1,68	-1,68	-0,75	EQMCE1	Equus caballus MHC class I antigen 3.7 (EQMCE1), mRNA [NM_001278933]	ATTCTTGCCATGAGGGATCAATGGGTTAGTTAAAGGAGAAGATTCCAAAAATTTGAGAGA
107	A_69_P013582	0,05	0,00	up	1,53	1,53	0,61	SLC31A1	solute carrier family 31 (copper transporter), member 1 [Source:HGNC Symbol;Acc:11016] [ENSECAT00000020834]	ATACTTTTTCTTCAGCTGGAAGAAGGCGGTGGTAGTGGATATCACAGAGCATTGCCATTA
108	A_69_P062215	0,05	0,00	down	1,57	-1,57	-0,65	PCDHB7	protocadherin beta 7 [Source:HGNC Symbol;Acc:8692] [ENSECAT00000001301]	GTCACAGAACACAGGGAGGGAAGTGGAAGAAAATCCTTCATTTGGGAATGATTTGGGTTT
109	A_69_P105626	0,05	0,00	up	1,39	1,39	0,47	ITGBL1	PREDICTED: Equus caballus integrin beta like 1 (ITGBL1), mRNA	TTCCTTTGTGTGTGTGTGTGTCTGTTTGATTTTGACTCTAGCAACACAAAATCAATAGAT
110	A_69_P004690	0,05	0,00	up	1,23	1,23	0,29	SMAD5	Equus caballus SMAD family member 5 (SMAD5), mRNA [NM_001163888]	TATGATTCCTCAGATTATGCCCAGTATATCCAGTAGAGATGTTCAGCCTGTTGCCTATGA
111	A_69_P020682	0,04	0,00	down	1,64	-1,64	-0,72	TMCC3	Uncharacterized protein [Source:UniProtKB/TrEMBL;Acc:F7AJN5] [ENSECAT00000012141]	TGTCGGCACCTTCTTTGCTGTGACTCTTCTTGCAATATTTTGTAAAAACTGGGACCATAT
112	A_69_P104364	0,04	0,00	down	1,20	-1,20	-0,27	DHRS11	PREDICTED: Equus caballus dehydrogenase/reductase (SDR family) member 11 (DHRS11), transcript variant X1, mRNA [XM_001501168]	ACCAGGGCCTGCCTCCCAAGTGGATTTCAGTATGATAATTAAAAGAGAAAAATCGCAACG
113	A_69_P090519	0,04	0,00	down	2,06	-2,06	-1,04	DMD	Uncharacterized protein [Source:UniProtKB/TrEMBL;Acc:F6Z1L7] [ENSECAT00000023688]	AGAGCCATGGAGTCCTTAGTTTCAGTCATGACAGATGAAGAAGGGGCAGAATAAATGTTT

114	A_69_P114728	0,05	0,00	up	1,46	1,46	0,55		HL02021A1F07 Equine placenta cDNA Library Equus caballus cDNA clone HL02021A1F07, mRNA sequence [DN510872]	ACTGTGGGACTTGGGTAAATAAATAAAGGGGATTCCACTCTGTGGGAACCACATCCAAAA
115	A_69_P089556	0,05	0,00	down	1,77	-1,77	-0,83	MYOZ2	myozenin 2 [Source:HGNC Symbol;Acc:1330] [ENSECAT00000018474]	CTTACTGGCAAAGCCACTTACACTCTTCATTGGTAGCAATAATTTTATAGCAATTTAGCA
116	A_69_P108571	0,03	0,00	down	1,16	-1,16	-0,22	TRAM2	REDICTED: Equus caballus translocation associated membrane protein 2 (TRAM2), mRNA	GGGGACTTTTTACTTTCAGTGAACTTTTTCCAAAATCCTTTTTCCACTGTTTCTGTCTGG
117	A_69_P092161	0,04	0,00	down	1,51	-1,51	-0,59	BEX1L	PREDICTED: Equus caballus protein BEX1-like (LOC100630227), mRNA	GCAGTTAGCATTGACCCCCCTCACCATGACCATCATGATGAGTTTTGCCTTATGCCTTGA
118	A_69_P019210	0,04	0,00	down	1,69	-1,69	-0,75	SYT17	synaptotagmin XVII [Source:HGNC Symbol;Acc:24119] [ENSECAT00000009185]	TGATCCTTTCTACAACGAGTCCTTCAGCTTCAAAGTTCCCCAAGAAGAACTGGAAAATGC
119	A_69_P059516	0,04	0,00	down	1,43	-1,43	-0,52		coiled-coil domain containing 88A [Source:HGNC Symbol;Acc:25523] [ENSECAT00000007818]	GACAAAGTACAAGAAAGCAGAAATTCAAAAAGCAGGTCTAGGGAGCAACAAAGCTCCTAA
120	A_69_P114729	0,05	0,00	up	1,46	1,46	0,54	POC1A	PREDICTED: Equus caballus POC1 centriolar protein A (POC1A), mRNA	TACTGTGGGACTTGGGTAAATAAATAAAGGGGATTCCACTCTGTGGGAACCACATCCAAA
121	A_69_P117121	0,05	0,00	down	1,41	-1,41	-0,49	CHCHD10	PREDICTED: Equus przewalskii coiled-coil-helix-coiled-coil-helix domain containing 10 (CHCHD10), mRNA	TGCAGAGGGGGTTTCTCACCCCACCAATGACCCGAGACACAAATGTGCAATTAAAAGAGT
122	A_69_P028206	0,05	0,00	up	1,29	1,29	0,37	MEF2BNB	MEF2B neighbor [Source:HGNC Symbol;Acc:37247] [ENSECAT00000007627]	TGTGGACAGTAGCGTCTACTTCCGCAGCGTGGAGGGTCTCCTGAAACAGGCCATCAGTAT

123	A_69_P113858	0,03	0,00	down	1,29	-1,29	-0,37	GRB10	PREDICTED: Equus caballus growth factor receptor bound protein 10 (GRB10), transcript variant X3, mRNA	GGCTCGTGAACCTAATTGTAAACTTTCAGGTATTTTTGTACAAATAAGGACTGATGTTC
124	A_69_P037952	0,04	0,00	down	1,66	-1,66	-0,73	EQMCE1	Equus caballus MHC class I antigen 3.7 (EQMCE1), mRNA [NM_001278933]	CTTGCCATGAGGGATCAATGGGTTAGTTAAAGGAGAAGATTCCAAAAATTTGAGAGAGGA
125	A_69_P002091	0,05	0,00	up	1,25	1,25	0,32	ARG2	arginase 2 [Source:HGNC Symbol;Acc:664] [ENSECAT00000017770]	TGTCTATGACCAACTTCCTACTCACCAGATGAATCACAAAGTGAAGAACTTGTGAGAATT
126	A_69_P119461	0,03	0,00	up	1,21	1,21	0,28	GLI1	Equus caballus GLI family zinc finger 1 (GLI1), mRNA [NM_001163860]	ATGAGTGTGTTACTGGGGTCCCTGCCTGGGGAGACACAATTCCTCAACTCTAGTGCCTAA
127	A_69_P076264	0,04	0,00	up	1,37	1,37	0,46	TBX15	Uncharacterized protein [Source:UniProtKB/TrEMBL;Acc:F6VPR5] [ENSECAT00000025103]	AGCCCAACACCTTTGAAAAACAGGAACTGTGTATTATTTTTATTTTCTTTTCTGGAGGAG
128	A_69_P114567	0,04	0,00	up	1,20	1,20	0,26	FAM134A	PREDICTED: Equus caballus family with sequence similarity 134 member A (FAM134A), partial mRNA	ACAGTGTGATATGTTTAGTGAGACTTAGCATGTGTGAATAAAGTGTATGCAGGAGGAAAA
129	A_69_P127216	0,04	0,00	up	1,39	1,39	0,47	TMPRSS9	transmembrane protease, serine 9 [Source:HGNC Symbol;Acc:30079] [ENSECAT00000015318]	AAGTGTCTCATCTCGGGCTGGGGCTACCTCAAGGAGGACTTCCTGGTCAAGCCGGAGAGG
130	A_69_P053051	0,05	0,00	down	1,66	-1,66	-0,73	GPD1	glycerol-3-phosphate dehydrogenase 1 (soluble) [Source:HGNC Symbol;Acc:4455] [ENSECAT00000001556]	TATAAGATATGCTACGAGAACCAGCCAGTGGGGGAATTCATCCACTGCCTACAAAATCAC
131	A_69_P103263	0,04	0,00	down	1,57	-1,57	-0,65	GNAI1	REDICTED: Equus caballus guanine nucleotide binding protein (G protein), alpha inhibiting activity polypeptide 1 (GNAI1), mRNA	ACGTACATATTGGTGACCATCATGGATGATTCCTGAACTTTATTCTGTGTAATTGTGTTA

132	A_69_P105473	0,04	0,00	down	2,04	-2,04	-1,03	CPE	CT02033A1F06 Equine Articular Cartilage cDNA Library Equus caballus cDNA clone CT02033A1F06, mRNA sequence [CX602587]	TGAAAGGAATAGAAACTGACTTGCTTGTACATATAGGGACAATGCCATTGTATTATTCAG
133	A_69_P090497	0,05	0,00	up	1,42	1,42	0,51	GK	glycerol kinase [Source:HGNC Symbol;Acc:4289] [ENSECAT00000006644]	TTGGGTTACAACTCAGTCTTCGGAAAGTGGTGACCCTAGTATCTTCTGTAGTCTACCCTT
134	A_69_P065076	0,04	0,00	down	1,18	-1,18	-0,24	RNF10	ring finger protein 10 [Source:HGNC Symbol;Acc:10055] [ENSECAT00000013662]	GGGGAACCAAGAAAATTTTAAATACAGTGTATTTTCCAGCTTCCTGTCTTTACACCAAAA
135	A_69_P013537	0,05	0,00	down	1,60	-1,60	-0,68	KIAA1958	KIAA1958 [Source:HGNC Symbol;Acc:23427] [ENSECAT00000008002]	AAGAAGGTCAAGCTGGAAAACTGTGAGAACTTCACCTTCGTCTCGTTTACTCAGGTGTCT
136	A_69_P106747	0,05	0,00	down	1,32	-1,32	-0,41	BRK1	PREDICTED: Equus caballus BRICK1, SCAR/WAVE actin-nucleating complex subunit (BRK1), mRNA	AGGTCAAGCCCAGATCCCCAATTGGCTTGAAGGGACATTTTCAGAGTTTTCTGTCATTTG
137	A_69_P086788	0,05	0,00	down	2,02	-2,02	-1,02	TRIM63	tripartite motif containing 63, E3 ubiquitin protein ligase [Source:HGNC Symbol;Acc:16007] [ENSECAT00000026380]	GCCATCGACTTTGGGACAGATGAGGAAGAGGAAGAGTTCTTTGAAGAAGAAGATCAGGAA
138	A_69_P095860	0,03	0,00	up	1,19	1,19	0,25	NDST2	Uncharacterized protein [Source:UniProtKB/TrEMBL;Acc:F7AV98] [ENSECAT00000019975]	TCTTAATCCAATGATTTCCCTCTTCATCCCCAGCAGTGTATGTATTTATACCTGACTATG
139	A_69_P095890	0,04	0,00	down	1,82	-1,82	-0,86	DUSP13	dual specificity phosphatase 13 [Source:HGNC Symbol;Acc:19681] [ENSECAT00000000110]	TGACCCAGTCTAGAGATTCTTTATGCAAAAGAGAGTTCAGTCTGTCTCTATAATAAAAGG

140	A_69_P010503	0,04	0,00	up	1,33	1,33	0,41	FADS1	fatty acid desaturase 1 [Source:HGNC Symbol;Acc:3574] [ENSECAT00000025250]	GTTTCCTTTTTCTAGTTTGGCAGATGCAGTAGGTTGGTGAGCAAAGAGGAAGTCAGGAGA
141	A_69_P105472	0,05	0,00	down	2,03	-2,03	-1,02	CPE	CT02033A1F06 Equine Articular Cartilage cDNA Library Equus caballus cDNA clone CT02033A1F06, mRNA sequence [CX602587]	TTGCTTGTACATATAGGGACAATGCCATTGTATTATTCAGCCTCTTAACACTACTTCAAA
142	A_69_P085919	0,04	0,00	down	1,37	-1,37	-0,45	MACF1	microtubule-actin crosslinking factor 1 [Source:HGNC Symbol;Acc:13664] [ENSECAT00000017611]	CGCGGTGCAGGGTTGTAAACCTGCTTTATCTTTTAGGATTATTCCTAAATGCATCTTCTT
143	A_69_P010875	0,04	0,00	down	1,13	-1,13	-0,18	ESRRA	estrogen-related receptor alpha [Source:HGNC Symbol;Acc:3471] [ENSECAT00000016651]	TGCTGTACAGAGACTCTATTTTAATGTATATTTGCTGCAAAGAGAAACCGCTTTTGGTTT
144	A_69_P116255	0,03	0,00	down	1,87	-1,87	-0,90		LeukoS2_3_C09.b1_A024 Stimulated peripheral blood leukocytes S2 Equus caballus cDNA clone LeukoS2_3_C09_A024 3', mRNA sequence [CD469289]	AGAAGAAGATTTTTAAAGTTTGAGAGTGGAAATAAATGGAAGCCCTGAGACCCTTCCAGA
145	A_69_P083007	0,04	0,00	down	1,27	-1,27	-0,34	HSD17B11	hydroxysteroid (17-beta) dehydrogenase 11 [Source:HGNC Symbol;Acc:22960] [ENSECAT00000008370]	ATCTTCCATACGCTTTTTAACACTGCTGGAAAGGATCTTTCCTGAGCGTTTCTTGGCAAT
146	A_69_P103631	0,04	0,00	down	1,60	-1,60	-0,68	SLC15A4	PREDICTED: Equus caballus solute carrier family 15 (oligopeptide transporter), member 4 (SLC15A4), mRNA [XM_005615190]	CCTCTGACTTCTGTTTCCTTATTTTACATGTATGTGGGGGTTCGCGTTTGTTAAATAAAC

147	A_69_P106761	0,04	0,00	down	1,23	-1,23	-0,29	IMPAD1	PREDICTED: Equus caballus inositol monophosphatase domain containing 1 (IMPAD1), mRNA	GCCAATTAGCTCTAGAAAATGTCATTGCCTCAAATTAAATGATTATCCCAAACTCTCTGC
148	A_69_P049781	0,04	0,00	down	1,42	-1,42	-0,50	LAMA2	laminin, alpha 2 [Source:HGNC Symbol;Acc:6482] [ENSECAT00000025657]	GATGGTGCTAATTCAGATCCCAGACTGAAGTTTAATTCAAATTCTTTCTCAAATCCATGA
149	A_69_P038319	0,04	0,00	down	1,25	-1,25	-0,32	DRB	Equus caballus MHC class II DR-beta chain (DRB), mRNA [NM_001142811]	TATGCACCTGAACTCACCTCTGCCCACATTTCTTTATAAAGTTTTTCTCAAATAAACATG
150	A_69_P103995	0,04	0,00	down	1,05	-1,05	-0,07	MLX	PREDICTED: Equus caballus MLX, MAX dimerization protein (MLX), transcript variant X2, mRNA [XM_001494111]	TGACAATTTTGGAAAATTAACCGGGCCTCAAAACTACTCCCAGCCTTTCTGTCAGGTGTA
151	A_69_P108113	0,05	0,00	up	1,15	1,15	0,20	NR2C2AP	PREDICTED: Equus caballus nuclear receptor 2C2-associated protein (NR2C2AP), transcript variant X5, mRNA	AACCTCAACTTCCCCTAAGTTTGTAAAAATGCATTTTGGAGGCTTTTCAGAGTCATAAAA
152	A_69_P058236	0,03	0,00	down	3,42	-3,42	-1,77	LOC100630878	Uncharacterized protein [Source:UniProtKB/TrEMBL;Acc:F7CYE1] [ENSECAT00000015366]	GCCACTCTGCTGTACGTGGAACATGCATGGTTTTCTTTAATCAGATGGAAGTGGTTTTCC
153	A_69_P036721	0,04	0,00	down	1,15	-1,15	-0,20	NOL7	nucleolar protein 7, 27kDa [Source:HGNC Symbol;Acc:21040] [ENSECAT00000000165]	GAGGTTTAAAAGACGGTGGATGGTCAGAAGGATGAAAACTTCTAAGAAGTAAATCAGAGC
154	A_69_P119427	0,04	0,00	down	1,09	-1,09	-0,13		TSA: Equus caballus contig06812.EqcaPBMC mRNA sequence. [JL616321]	CACTTGAATGGACTGAAAATTTGCTGGATTATCTAGAACAACAAGATGACATGCTTCTGT
155	A_69_P037951	0,04	0,00	down	1,61	-1,61	-0,69	EQMCE1	Equus caballus MHC class I antigen 3.7 (EQMCE1), mRNA [NM_001278933]	TTGCCATGAGGGATCAATGGGTTAGTTAAAGGAGAAGATTCCAAAAATTTGAGAGAGGAA

156	A_69_P074357	0,04	0,00	up	1,93	1,93	0,95	LOC100057656	CD55 molecule, decay accelerating factor for complement (Cromer blood group) [Source:HGNC Symbol;Acc:2665] [ENSECAT00000017807]	ATAGTTCTCAGATTTCCAAGGTCACACCAGCGGTTCAGAAACCCACCACTGTAGATGTTA
157	A_69_P031058	0,03	0,00	up	1,89	1,89	0,92	ABI3BP	PREDICTED: Equus asinus ABI family member 3 binding protein (ABI3BP), transcript variant X31, mRNA [ENSECAT00000002615]	AAAGAAGGCTATTTCAGAGCAGTTCGCCAGGAACCTGTCCAATTTGGAGAAATAGGTGGT
158	A_69_P104871	0,05	0,00	down	2,27	-2,27	-1,18	ANG	PREDICTED: Equus caballus angiogenin, ribonuclease, RNase A family, 5 (ANG), transcript variant X1, mRNA [XM_005602591]	ACTCTTCCCAAATGAAAGATTTTTCATGTTTTCCTTGTAATAAAGGCCCTAAATTGCAGG
159	A_69_P102981	0,05	0,00	down	1,17	-1,17	-0,23	DPYD	PREDICTED: Equus caballus dihydropyrimidine dehydrogenase (DPYD), transcript variant X2, mRNA	GGAAAAAGAGGAATATTGGCAACAGATGTTAGCTCAGGGCCAATCTTCCTCACCAAAAAA
160	A_69_P119691	0,05	0,00	up	1,09	1,09	0,12	ZNF786	PREDICTED: Equus caballus zinc finger protein 786 (ZNF786), mRNA [XM_001492819]	TAATGCTCCAGACAAGCCTGACGTTAAGAAAAGGCTTAGTCAGCTGTTGGCAATGATCGA
161	A_69_P118906	0,05	0,00	down	1,27	-1,27	-0,35	TAB2	PREDICTED: Equus caballus TGF-beta activated kinase 1/MAP3K7 binding protein 2 (TAB2), transcript variant X2, mRNA	CCTTGTTTTTCAGCTTCATCTGCAGTTCTATGTGAAGATTGATCAATCAGTTTTTACTTG
162	A_69_P022612	0,04	0,00	down	1,35	-1,35	-0,43	DCAF5	DDB1 and CUL4 associated factor 5 [Source:HGNC Symbol;Acc:20224] [ENSECAT00000010943]	GCCATAGTGGCCTCAAAAGGCACCGAGTTGAATTGGAAGATACAGATTCAGAGAATTCCT

163	A_69_P033701	0,05	0,00	up	1,27	1,27	0,34	HOXB13	homeobox B13 [Source:HGNC Symbol;Acc:5112] [ENSECAT00000014223]	TCACCATCTGGTTTCAGAACCGCCGGGTCAAGGAGAAGAAGGTCCTCGCCAAGGTCAAGA
164	A_69_P029847	0,03	0,00	up	1,80	1,80	0,85	B3GNT5	UDP-GlcNAc:betaGal beta-1,3-N-acetylglucosaminyltransferase 5 [Source:HGNC Symbol;Acc:15684] [ENSECAT00000001723]	CACAGACCCAAAAGTGAAAACAATTTCAAAAGGGTTTTATGGTCAAATATACTGCAGGAT
165	A_69_P119311	0,04	0,00	up	1,26	1,26	0,34	TMEM159	transmembrane protein 159 [Source:HGNC Symbol;Acc:30136] [ENSECAT00000011006]	TTTGTGTCCTCTGTGGCTTGGGTTTCGTGTCACTCATCATATCGGGGACAATTATAGTGT
166	A_69_P065074	0,05	0,00	up	1,36	1,36	0,44	POP5	processing of precursor 5, ribonuclease P/MRP subunit (S. cerevisiae) [Source:HGNC Symbol;Acc:17689] [ENSECAT00000010367]	CATGTCAGAAGTTCCTGATTCAGTACAACCGGAGACAGCTGTTGATCTTGTTGCAACACT
167	A_69_P102194	0,03	0,00	up	5,15	5,15	2,37		APL1_9_G09.g1_A008 Liver (APL1) Equus caballus cDNA, mRNA sequence [BM780851]	CCACAACTTGCCTGTTTCTATGAGATCTGTGAATTCCAAATAAACGTGTGTTGACTCTAA
168	A_69_P011646	0,04	0,00	down	1,16	-1,16	-0,21	KCNQ1	potassium voltage-gated channel, KQT-like subfamily, member 1 [Source:HGNC Symbol;Acc:6294] [ENSECAT00000026942]	AAAGCCCTCCCTCTTCATCTCCGGCTCAGAAAAGAGCAAGGACCGCGGCAATAACACCAT
169	A_69_P009021	0,05	0,00	down	1,35	-1,35	-0,43	LDLRAD3	low density lipoprotein receptor class A domain containing 3 [Source:HGNC Symbol;Acc:27046] [ENSECAT00000020348]	TTGTGACTTCGGAGAACCAGCTGGTCTACTACCCCAGCATCACCTACGCCATCATCGGCA

170	A_69_P094796	0,03	0,00	down	1,11	-1,11	-0,15	SLC25A28	solute carrier family 25 (mitochondrial iron transporter), member 28 [Source:HGNC Symbol;Acc:23472] [ENSECAT00000009644]	CTCGACTGTTATAGAGGGATAAGAAGCTCATTCCCCTGGTTCCTAATAAAAAGTCTTTAA
171	A_69_P111086	0,05	0,00	down	1,12	-1,12	-0,17	UBE4A	PREDICTED: Equus caballus ubiquitination factor E4A (UBE4A), mRNA [XM_001502841]	GAGGGTAATACTAGGAAAATTATTTAAGTAGGTTTTATCAAGCAATCTGGGTTGGTTTGG
172	A_69_P037954	0,04	0,00	down	1,65	-1,65	-0,72	EQMCE1	Equus caballus MHC class I antigen 3.7 (EQMCE1), mRNA [NM_001278933]	TTCTTGCCATGAGGGATCAATGGGTTAGTTAAAGGAGAAGATTCCAAAAATTTGAGAGAG
173	A_69_P051554	0,04	0,00	up	1,37	1,37	0,46	VAMP1	vesicle-associated membrane protein 1 (synaptobrevin 1) [Source:HGNC Symbol;Acc:12642] [ENSECAT00000015343]	GAAAAACTGCAAGATGATGATCATGCTGGGAGCTATCTGTGCCATCGTCGTGGTAGTTAT
174	A_69_P103262	0,04	0,00	down	1,55	-1,55	-0,63	GNAI1	PREDICTED: Equus caballus guanine nucleotide binding protein (G protein), alpha inhibiting activity polypeptide 1 (GNAI1), mRNA	CGTACATATTGGTGACCATCATGGATGATTCCTGAACTTTATTCTGTGTAATTGTGTTAC
175	A_69_P107787	0,05	0,00	down	1,66	-1,66	-0,73	SVIL	PREDICTED: Equus caballus supervillin (SVIL), transcript variant X1, mRNA	AAAGAACAATTTGCTAACCACAAAAGAGGAATCGATGCTAAGCTTTTAACTGCACTTCCT
176	A_69_P086566	0,05	0,00	down	1,29	-1,29	-0,37	PTAFR	eyes absent homolog 3 (Drosophila) [Source:HGNC Symbol;Acc:3521] [ENSECAT00000018417]	TGAAGAAATTGCAGCCAAACAGCACAACATGCCTTTCTGGAGAATCACAAACCATGGAGA
177	A_69_P001357	0,04	0,00	up	1,13	1,13	0,18	PRKAA1	Equus caballus protein kinase, AMP-activated, alpha 1 catalytic subunit (PRKAA1), mRNA [NM_001081803]	ATCAAGTTATGTATAACTATAACTGAAGATTGGCCTTTTGGAATGCAGTTTGCACAGGGA

178	A_69_P070291	0,05	0,00	up	1,31	1,31	0,39	S1PR2	sphingosine-1-phosphate receptor 2 [Source:HGNC Symbol;Acc:3169] [ENSECAT00000004599]	ATTATCTTGTTGGCCATCGTGGTGCTGTACATCCGCATCTACTGCGTGGTCCGCTCCAGC
179	A_69_P078175	0,04	0,00	up	1,09	1,09	0,12		mitochondrial ribosomal protein S24 [Source:HGNC Symbol;Acc:14510] [ENSECAT00000015296]	GAATAAATAGACTCCATGTGTCTTGCCTCTCAGTCTGTCTTTCCTCTTTAGGTGAGGGAA
180	A_69_P017490	0,04	0,00	up	1,23	1,23	0,30	NUDT1	nudix (nucleoside diphosphate linked moiety X)-type motif 1 [Source:HGNC Symbol;Acc:8048] [ENSECAT00000009517]	TGACAGATACTGGTTTCCCCTCTTGCTTCAGAAGAAGAAATTCCATGGGTACTTCAAGTT
181	A_69_P071231	0,05	0,00	up	1,17	1,17	0,23	MRPL48	Mitochondrial 39S ribosomal protein L48-like protein; Uncharacterized protein [Source:UniProtKB/TrEMBL;Acc:F6XF35] [ENSECAT00000023582]	TAATCCAAAGCAATCTTCCTGAAGGAGTCAAACTGTCAGTGAGAGAGCACACTGAAGAGG
182	A_69_P070886	0,04	0,00	down	1,95	-1,95	-0,96	SYTL2	synaptotagmin-like 2 [Source:HGNC Symbol;Acc:15585] [ENSECAT00000007290]	ACTTGGATTGAAGCAACCCTGCCTCTCAGAATGCTGTTGATTGCCAAAATTTCCAAATGA
183	A_69_P063421	0,05	0,00	up	1,39	1,39	0,48	EDIL3	EGF-like repeats and discoidin I-like domains 3 [Source:HGNC Symbol;Acc:3173] [ENSECAT00000016344]	CACGAGTTGATAGAGCCTTTATAATCAGTACTCAGCTAATTCTTATAAAAGACATACTGC
184	A_69_P103261	0,04	0,00	down	1,56	-1,56	-0,64		LeukoN1_2_H10.g1_A023 Unstimulated peripheral blood leukocytes N1 Equus caballus cDNA clone LeukoN1_2_H10_A023 5', mRNA sequence [CD465244]	GTACATATTGGTGACCATCATGGATGATTCCTGAACTTTATTCTGTGTAATTGTGTTACT

185	A_69_P104736	0,04	0,00	down	1,23	-1,23	-0,30	GHITM	PREDICTED: Equus asinus growth hormone inducible transmembrane protein (GHITM), transcript variant X1, mRNA	TAACCGCCTCAGATCACATAGGATGCTTGTTTCTGAAAGAATAAAGGTTCCAGCCCGAAA
186	A_69_P096141	0,05	0,00	down	1,68	-1,68	-0,75	CHRM3	cholinergic receptor, muscarinic 3 [Source:HGNC Symbol;Acc:1952] [ENSECAT00000010538]	GATGAAAATGTTTTTATCACCCAGATATGAAAGAAGCTGCCTGTTTACTGATCCATTGAA
187	A_69_P038316	0,05	0,00	down	1,23	-1,23	-0,30	DRB	Equus caballus MHC class II DR-beta chain (DRB), mRNA [NM_001142811]	GCACCTGAACTCACCTCTGCCCACATTTCTTTATAAAGTTTTTCTCAAATAAACATGGAG
188	A_69_P068736	0,04	0,00	down	1,16	-1,16	-0,21	TRAPPC4	trafficking protein particle complex 4 [Source:HGNC Symbol;Acc:19943] [ENSECAT00000006099]	TATTCAGACTTTGCCCTCAAGAATCCTTTCTACTCCCTGGAAATGCCAATCAGGTGTGAG
189	A_69_P055611	0,03	0,00	down	1,18	-1,18	-0,24	SPCS1	Uncharacterized protein [Source:UniProtKB/TrEMBL;Acc:F6U4C1] [ENSECAT00000025246]	AAATTTCAAACCCAAAACATGGGCTAAAACCCATCTATGCTCTTACAGCACAGCTGTCTT
190	A_69_P116191	0,03	0,00	down	8,02	-8,02	-3,00			TTTAGGGACCCTGATCCAATGTTTCCATGGAAGCAATAAATTTTCCTTTATGAGTTCTTT
191	A_69_P092438	0,05	0,00	down	1,29	-1,29	-0,37	CAPN6	calpain 6 [Source:HGNC Symbol;Acc:1483] [ENSECAT00000011583]	AACTCCCCAATCCCCATCACCAACAATATTTCAATGCTTCTGCAATGATGAAAAAGAAAA
192	A_69_P015956	0,05	0,00	down	1,10	-1,10	-0,13	STOX2	Uncharacterized protein [Source:UniProtKB/TrEMBL;Acc:F6S2T9] [ENSECAT00000013310]	AATTTCATTTTTAAATGCCACAGATGCGTGTTGCTCCCAGCCTGTGGTCAAATGGTGCCA
193	A_69_P118777	0,03	0,00	down	3,35	-3,35	-1,74	LOC102147820	PREDICTED: Equus caballus myelin and lymphocyte protein-like (LOC102147820), mRNA [XM_005600243]	CTACATGGTCCATGCGGTGTTTTCTTTAATCAGATGGAAGAAAGCAGCAGAAGAACTTTA

194	A_69_P105676	0,04	0,00	down	1,27	-1,27	-0,34	LANCL1	PREDICTED: Equus caballus LanC lantibiotic synthetase component C-like 1 (bacterial) (LANCL1), transcript variant X2, mRNA	AGGCCTGGGATCGTATGTATTTCTCTGTTCTGTAAAGGATTTAACTTACTGCTTCTCAAT
195	A_69_P087793	0,04	0,00	down	1,20	-1,20	-0,26	TNFRSF25	tumor necrosis factor receptor superfamily, member 25 [Source:HGNC Symbol;Acc:11910] [ENSECAT00000020471]	AAGTACGGTTACTTATGCGTGTAGACATTTTATGTCACTTATTAAGCCCCTGGCGCCGAC
196	A_69_P086787	0,05	0,00	down	1,85	-1,85	-0,89	TRIM63	tripartite motif containing 63, E3 ubiquitin protein ligase [Source:HGNC Symbol;Acc:16007] [ENSECAT00000026380]	CCATCGACTTTGGGACAGATGAGGAAGAGGAAGAGTTCTTTGAAGAAGAAGATCAGGAAG
197	A_69_P055376	0,04	0,00	up	1,30	1,30	0,37		chromosome 3 open reading frame 67 [Source:HGNC Symbol;Acc:24763] [ENSECAT00000012445]	GACCCATGTCTGAACTGTTACTTTGATCCTCAGACTGGGAAATACTACGAGTTGGTGTAA
198	A_69_P076726	0,05	0,00	down	1,47	-1,47	-0,56	SYPL2	synaptophysin-like 2 [Source:HGNC Symbol;Acc:27638] [ENSECAT00000011974]	AACTTCTTCCTGTGGGCCGGGAACTGTTGGTTTGTGTTCAAGGAGACCCCATGGCATGGG
199	A_69_P081256	0,04	0,00	up	5,68	5,68	2,51	LOC100630653	Metallothionein [Source:UniProtKB/TrEMBL;Acc:F7DUC3] [ENSECAT00000000277]	TTTGGATCTCAGGACTCAACAAGGCTCGGAATGGATCCCAACTGCTCGTGCCCCACTGGT
200	A_69_P004372	0,05	0,00	up	1,25	1,25	0,32		Equus caballus subtracted library fragment 40. [AY246804]	TATAGGTATATGGTAAATGTAGCCTCCCCTGACACAGGAAGGGTTAATAGTCACTGCCAA
201	A_69_P102590	0,03	0,00	up	1,78	1,78	0,83	ABCA1	PREDICTED: Equus caballus ATP-binding cassette, sub-family A (ABC1), member 1 (ABCA1), mRNA [XM_001493790]	TCAGAGATTCCTGCCTTTGCTTCTTTTGGACACTCTGGAAACCTTATTAACAACTGTGAA

202	DCP_22_0	0,03	0,00	up	6,29	6,29	2,65		Unknown	
203	A_69_P018974	0,04	0,00	up	1,14	1,14	0,20	USP31	ubiquitin specific peptidase 31 [Source:HGNC Symbol;Acc:20060] [ENSECAT00000006222]	TCTTCAAAAAAGTTATCTTCTAGCATGCAAACCTCTGCACGGCCTTCTCAAAAACCTCAG
204	A_69_P099794	0,05	0,00	up	1,39	1,39	0,48	RNASE6	ribonuclease, RNase A family, k6 [Source:HGNC Symbol;Acc:10048] [ENSECAT00000012091]	GACCCTCCATATCACTTGGTTCCTGTACACTTAGATAGTATTGTTTAAAGTTTCCACCAT
205	A_69_P113231	0,04	0,00	down	2,06	-2,06	-1,04	TPD52	PREDICTED: Equus caballus tumor protein D52 (TPD52), transcript variant X6, mRNA	GGCCTGCATGTGCCTATCACCTGCCGGGATTTCATACTTAAATAAATGCTATTTGACGAC
206	A_69_P073516	0,04	0,00	down	1,12	-1,12	-0,16	KIFAP3	kinesin-associated protein 3 [Source:HGNC Symbol;Acc:17060] [ENSECAT00000005585]	CTGTATTAAGTACAATTGATGCTTGACACTGTTCAGTCATCAAGTCACATCTTGATGCTG
207	A_69_P112708	0,05	0,00	up	1,23	1,23	0,30	MFSD10	PREDICTED: Equus caballus major facilitator superfamily domain containing 10 (MFSD10), mRNA	CTCCCAAGCCTGTGTCGTATATGCCACTAAACTTCATGTTGTAATACAAAGAGTTAAAAT
208	A_69_P002411	0,03	0,00	down	4,70	-4,70	-2,23	DRA	Equus caballus MHC class II DR alpha chain (DRA), mRNA [NM_001284542]	AATCATTGTTGGGACCATCTTCATCATCAAGGGTGTGCGCAAAGGCAATACTGTTGAACG
209	A_69_P103265	0,04	0,00	down	1,55	-1,55	-0,63		LeukoN1_2_H10.g1_A023 Unstimulated peripheral blood leukocytes N1 Equus caballus cDNA clone LeukoN1_2_H10_A023 5', mRNA sequence [CD465244]	AGACGTACATATTGGTGACCATCATGGATGATTCCTGAACTTTATTCTGTGTAATTGTGT
210	A_69_P061006	0,05	0,00	up	1,17	1,17	0,23	GRM6	glutamate receptor, metabotropic 6 [Source:HGNC Symbol;Acc:4598] [ENSECAT00000025528]	AGTGCGTCGGTGTCCCTGGGCATGCTCTATGTACCCAAAACCTACGTCATCCTGTTCCAC

211	A_69_P114669	0,04	0,00	down	1,59	-1,59	-0,67		HL02020B1G03 Equine placenta cDNA Library Equus caballus cDNA clone HL02020B1G03, mRNA sequence [DN510748]	GGTTCTGATTCTATGGCTCTTATGCATCAGTTGTGGACATGGACTTTAAATAAATATACA
212	A_69_P019931	0,04	0,00	up	1,35	1,35	0,43	GFER	growth factor, augmenter of liver regeneration [Source:HGNC Symbol;Acc:4236] [ENSECAT00000017991]	TAAGTTTTACCCGTGTGAGGAGTGTGCTGAAGACATAAGAAAGAGGATACGCAGGAACCA
213	A_69_P114546	0,05	0,00	down	1,44	-1,44	-0,53	TMCC3	PREDICTED: Equus caballus transmembrane and coiled-coil domain family 3 (TMCC3), transcript variant X4, mRNA	GCATCAAATGGGAACGTTTTTGTATTGAATCCAAATCTAATTATGTTGGTTGGGGAAAAC
214	A_69_P065391	0,04	0,00	up	1,21	1,21	0,27	PPP1CC	protein phosphatase 1, catalytic subunit, gamma isozyme [Source:HGNC Symbol;Acc:9283] [ENSECAT00000018299]	ACTTGCCAGCATCACTGGTATGATGTATATTTAATTAAAGCACACTTTCCCCTACTGTAT
215	A_69_P021736	0,05	0,00	up	1,26	1,26	0,33	TTLL1	tubulin tyrosine ligase-like family, member 1 [Source:HGNC Symbol;Acc:1312] [ENSECAT00000013441]	ACAAGTCACCCCCAAAGGAAGTTCTTGGCAATTATGAAATTCTGTACGACGAAGAGCTGG
216	A_69_P105916	0,05	0,00	down	1,26	-1,26	-0,33	SPG11	Select seq ref|XM_014733570.1| PREDICTED: Equus caballus spastic paraplegia 11 (autosomal recessive) (SPG11), mRNA	CTTCTACTTTAGGAGATGATACAATATCGATTAAAGAATATCTATGCCTGCAGATTCCAG
217	A_69_P116901	0,04	0,00	up	1,88	1,88	0,91	ABI3BP	PREDICTED: Equus caballus ABI family member 3 binding protein (ABI3BP), transcript variant X26, mRNA	GAAAATCTGACTCATTTTTATTTAAAAAGGTATTGCTTTCTTTCACTGGGGGAGGGAGAC

218	A_69_P019211	0,04	0,00	up	1,36	1,36	0,44	SMG1	Uncharacterized protein [Source:UniProtKB/TrEMBL;Acc:F7BC08] [ENSECAT00000011670]	TTTAGCCAACACTCTTGTTGAAGATGTGAATATCTGTCTGCAGGCGTGCAGCAGTCTACA
219	A_69_P117288	0,04	0,00	down	2,01	-2,01	-1,01			TGCCATTGTATTATTCAGCCTCTTAACACTACTTCAAAGTTTAGGGTTTTCTCTTGGTTG
220	A_69_P004150	0,05	0,00	up	1,11	1,11	0,14	SOD2	Equus caballus manganese superoxide dismutase (Mn-SOD) mRNA, 3' UTR and partial cds. [AY246751]	AGGCCTGATTATCTGAAAGCCATTTGGAATGTGATCAACTGGGAGAATGTATCTGAAAGA
221	A_69_P119693	0,05	0,00	up	1,15	1,15	0,20	ZNF786	PREDICTED: Equus caballus zinc finger protein 786 (ZNF786), mRNA [XM_001492819]	GATAATGCTCCAGACAAGCCTGACGTTAAGAAAAGGCTTAGTCAGCTGTTGGCAATGATC
222	A_69_P088941	0,03	0,00	down	1,35	-1,35	-0,43	GUCY1A3	guanylate cyclase 1, soluble, alpha 3 [Source:HGNC Symbol;Acc:4685] [ENSECAT00000006300]	CCACCAAACTTCCCCAGTGAGATTGGAATCTGTTATTTTCTGGAAGCTTATCAACAAGGA
223	A_69_P095966	0,04	0,00	up	1,31	1,31	0,39	PLAC9	placenta-specific 9 [Source:HGNC Symbol;Acc:19255] [ENSECAT00000011851]	AGAGCCGTGCATTCTGGCCTTCCTTCCTCAGCTTTGTACACAATAAAAGCTACTTCTTTG
224	A_69_P097941	0,05	0,00	up	1,31	1,31	0,39	UACA	uveal autoantigen with coiled-coil domains and ankyrin repeats [Source:HGNC Symbol;Acc:15947] [ENSECAT00000017420]	AATTGCAATTTATCGGACACACCTTCTTAGTGCTGCACAGGGTCACATGGATGAAGACGT
225	A_69_P118779	0,04	0,00	down	3,79	-3,79	-1,92	LOC102147820	PREDICTED: Equus caballus myelin and lymphocyte protein-like (LOC102147820), mRNA [XM_005600243]	CTCTACATGGTCCATGCGGTGTTTTCTTTAATCAGATGGAAGAAAGCAGCAGAAGAACTT

226	A_69_P086921	0,04	0,00	down	1,30	-1,30	-0,38	IL22RA1	interleukin 22 receptor, alpha 1 [Source:HGNC Symbol;Acc:13700] [ENSECAT00000017099]	AAATCCTTCCAGCAGCATCTGGGGGTTCACACGGACAGCACACCTGACCCTGATGTGCTA
227	A_69_P090520	0,03	0,00	down	2,09	-2,09	-1,06		Uncharacterized protein [Source:UniProtKB/TrEMBL;Acc:F6Z1L7] [ENSECAT00000023688]	CAGAGCCATGGAGTCCTTAGTTTCAGTCATGACAGATGAAGAAGGGGCAGAATAAATGTT
228	A_69_P055306	0,05	0,00	down	1,11	-1,11	-0,15	PSMD6	proteasome (prosome, macropain) 26S subunit, non-ATPase, 6 [Source:HGNC Symbol;Acc:9564] [ENSECAT00000022688]	CAAGAAACTATCAAGAAAGGAGATCTGCTACTAAACAGAGTTCAGAAACTTTCCAGAGTA
229	A_69_P090012	0,03	0,00	up	1,17	1,17	0,23	PRPS2	phosphoribosyl pyrophosphate synthetase 2 [Source:HGNC Symbol;Acc:9465] [ENSECAT00000002926]	CAGGAGGACAAGATGAGACACTGCTCCAAGATTCAGGTGATTGACATTTCGATGATCTTG
230	A_69_P074553	0,05	0,00	down	1,26	-1,26	-0,34	B4GALT3	UDP-Gal:betaGlcNAc beta 1,4-galactosyltransferase, polypeptide 3 [Source:HGNC Symbol;Acc:926] [ENSECAT00000023482]	TCCCTCTTCTGGACCTGGCTCAGGGGGCTGGGATTTTGATATATTTTCTAATAAAGGACT
231	A_69_P117289	0,05	0,00	down	2,00	-2,00	-1,00		CT02033A1F06 Equine Articular Cartilage cDNA Library Equus caballus cDNA clone CT02033A1F06, mRNA sequence [CX602587]	TTGCTTGTACATATAGGGACAATGCCATTGTATTATTCAGCCTCTTAACACTACTTCAAA
232	A_69_P095857	0,04	0,00	up	1,17	1,17	0,23	NDST2	Uncharacterized protein [Source:UniProtKB/TrEMBL;Acc:F7AV98] [ENSECAT00000019975]	TAATCCAATGATTTCCCTCTTCATCCCCAGCAGTGTATGTATTTATACCTGACTATGGGA

233	A_69_P100403	0,04	0,00	down	1,56	-1,56	-0,64	SLC7A7	solute carrier family 7 (amino acid transporter light chain, y+L system), member 7 [Source:HGNC Symbol;Acc:11065] [ENSECAT00000001184]	TCCTCTTTTCCTTTAAAAGGGCCAATAATGCTCCAAACTTCCGTCTCCTTTAGAGAGACA
234	A_69_P012852	0,04	0,00	up	1,13	1,13	0,18	RGP1	RGP1 retrograde golgi transport homolog (S. cerevisiae) [Source:HGNC Symbol;Acc:21965] [ENSECAT00000010431]	AGACAGGCTTTCAGCTGTAGCATTAATTTATTCAGAATAAACTGGCAGCTGCTAGTGGTT
235	A_69_P116226	0,03	0,00	down	1,44	-1,44	-0,53	MAFK	PREDICTED: Equus przewalskii v-maf avian musculoaponeurotic fibrosarcoma oncogene homolog K (MAFK), mRNA	AAGGTGGCCACCACCAGCGTCATCACCATCGTCAAGTCCGCTGAGATCTCCAGCTCCGTG
236	A_69_P089347	0,04	0,00	up	1,21	1,21	0,28		Enolase [Source:UniProtKB/TrEMBL;Acc:F6WCY1] [ENSECAT00000024763]	AAATTGTGCAATTGCCTGCTGCTCAAAGCGAACCACATCAACTCTTTGACCAAGTCTCTT
237	A_69_P122861	0,05	0,00	down	1,50	-1,50	-0,58	PLCB1	PREDICTED: Equus caballus phospholipase C beta 1 (PLCB1), mRNA	GGCATGATGTTTCTTCCACTTGTAATTTTATGTGCTTTCATCACAAATCCAAAGGAAACA
238	A_69_P102191	0,03	0,00	up	5,11	5,11	2,35		APL1_9_G09.g1_A008 Liver (APL1) Equus caballus cDNA, mRNA sequence [BM780851]	CAACTTGCCTGTTTCTATGAGATCTGTGAATTCCAAATAAACGTGTGTTGACTCTAAAAA
239	A_69_P117287	0,04	0,00	down	1,93	-1,93	-0,95	CPE	PREDICTED: Equus caballus carboxypeptidase E (CPE), mRNA [XM_001915786]	TCTTAACACTACTTCAAAGTTTAGGGTTTTCTCTTGGTTGTAGATTGGCCCAGATTTGCA
240	A_69_P112363	0,03	0,00	up	1,12	1,12	0,17	ENSA	PREDICTED: Equus caballus endosulfine alpha (ENSA), transcript variant 1, mRNA [XM_001490179]	CCCACAGAGCCCAGGGGACTTTTTCAGTATTTGAAATAAAAAGAAAAGAAGACCCACAA

241	A_69_P040761	0,04	0,00	down	1,29	-1,29	-0,36	RNF219	ring finger protein 219 [Source:HGNC Symbol;Acc:20308] [ENSECAT00000011765]	CAAAAAGAAAAATCCAGAGCAGTCTTTCCAATGCCAGCCCATCAAAAGCAACTAAAAGTT
242	A_69_P055378	0,05	0,00	up	1,35	1,35	0,43		chromosome 3 open reading frame 67 [Source:HGNC Symbol;Acc:24763] [ENSECAT00000012445]	ATGACCCATGTCTGAACTGTTACTTTGATCCTCAGACTGGGAAATACTACGAGTTGGTGT
243	A_69_P102195	0,03	0,00	up	5,06	5,06	2,34		APL1_9_G09.g1_A008 Liver (APL1) Equus caballus cDNA, mRNA sequence [BM780851]	ACCACAACTTGCCTGTTTCTATGAGATCTGTGAATTCCAAATAAACGTGTGTTGACTCTA
244	A_69_P062468	0,04	0,00	down	1,20	-1,20	-0,26	FAM53C	family with sequence similarity 53, member C [Source:HGNC Symbol;Acc:1336] [ENSECAT00000001697]	CTGTGTGTGTTAAACATGAGGTCCTGCCTCTGTGGCTGTGTTTGAAAAAATAAAGTTTTA
245	A_69_P040651	0,04	0,00	down	1,22	-1,22	-0,28	PCDH9	protocadherin 9 [Source:HGNC Symbol;Acc:8661] [ENSECAT00000018446]	GATAGATTGCGACTAAGCCATATAGGAAGTCTTCTCTTTGATTTCAGATGAGTTTATTGT
246	A_69_P063021	0,04	0,00	down	2,29	-2,29	-1,20	CDO1	cysteine dioxygenase type 1 [Source:HGNC Symbol;Acc:1795] [ENSECAT00000013286]	TATTGTAGATAGAGAAACTTAAGGTACTTTGCCATTTGGTAGTGAAATGAAGGGCAGAGG
247	A_69_P064742	0,05	0,00	down	1,82	-1,82	-0,86	C8H22orf31	chromosome 22 open reading frame 31 [Source:HGNC Symbol;Acc:26931] [ENSECAT00000011961]	CTCATGGGATCAGGATATTCTCTGCTAGATGTCTGAAAAATAAACACCACTTTGCACCTT
248	A_69_P108994	0,04	0,00	down	1,77	-1,77	-0,83	FAM107A	Select seq ref|XM_005600506.2| PREDICTED: Equus caballus family with sequence similarity 107 member A (FAM107A), transcript variant X2, mRNA	TGTTGCTTTCATCAGAGATGTAAATAATTGCCAATCTGCATTTCTTACCACACTGTTATG

249	A_69_P118780	0,03	0,00	down	3,39	-3,39	-1,76	MAL	PREDICTED: Equus caballus myelin and lymphocyte protein-like (LOC102147820), mRNA [XM_005600243]	GCTCTACATGGTCCATGCGGTGTTTTCTTTAATCAGATGGAAGAAAGCAGCAGAAGAACT
250	A_69_P104115	0,05	0,00	up	1,16	1,16	0,22	IPO9	PREDICTED: Equus caballus importin 9 (IPO9), mRNA [XM_005608061]	AAGTGGTTAAGTTGTAGCCCTCAAATTTGCAACGTGTATTTTTCTAGGAGAGTAAAGTAA
251	A_69_P032363	0,05	0,00	down	1,19	-1,19	-0,25	C11H17orf58	chromosome 17 open reading frame 58 [Source:HGNC Symbol;Acc:27568] [ENSECAT00000018026]	CATTTCTGGACCGTAAGAGACTTGGAATTCGGCAACAAGACAGGAAAGGTCTTCATGTCA
252	A_69_P033771	0,05	0,00	up	1,22	1,22	0,29	PHB	prohibitin [Source:HGNC Symbol;Acc:8912] [ENSECAT00000018748]	TAATCACTGGTAGCAAAGATTTACAGAATGTCAACATCACCCTGCGCATCCTTTTCCGGC
253	A_69_P087806	0,04	0,00	up	1,24	1,24	0,31	ACOT7	acyl-CoA thioesterase 7 [Source:HGNC Symbol;Acc:24157] [ENSECAT00000025347]	TAACTTTCATGACAAGATCAGAAAAGGGTGTGTCATCACCATCTCTGGGCGCATGACCTT
254	A_69_P061081	0,04	0,00	up	1,23	1,23	0,30	B4GALT7	xylosylprotein beta 1,4-galactosyltransferase, polypeptide 7 [Source:HGNC Symbol;Acc:930] [ENSECAT00000011688]	ACATCATGTTGGACTGCGACAAGGCAGCTACCCCCTGGTGCACATTTGGCTGAGTTGGCT
255	A_69_P097216	0,04	0,00	down	1,29	-1,29	-0,37	ASB7	ankyrin repeat and SOCS box containing 7 [Source:HGNC Symbol;Acc:17182] [ENSECAT00000004079]	CCTAAAGCTACTTGATGAACTACCAATTGCCAAGGTCATGAAAGACTACTTAAAACACAA
256	A_69_P108993	0,04	0,00	down	1,84	-1,84	-0,88	FAM107A	CT02036A2F04 Equine Articular Cartilage cDNA Library Equus caballus cDNA clone CT02036A2F04, mRNA sequence [CX603734]	GTTGCTTTCATCAGAGATGTAAATAATTGCCAATCTGCATTTCTTACCACACTGTTATGA

257	A_69_P116251	0,05	0,00	down	2,12	-2,12	-1,08		LeukoS2_3_C09.b1_A024 Stimulated peripheral blood leukocytes S2 Equus caballus cDNA clone LeukoS2_3_C09_A024 3', mRNA sequence [CD469289]	GAAGATTTTTAAAGTTTGAGAGTGGAAATAAATGGAAGCCCTGAGACCCTTCCAGAAGTC
258	A_69_P076032	0,04	0,00	up	1,42	1,42	0,50	MTMR11	myotubularin related protein 11 [Source:HGNC Symbol;Acc:24307] [ENSECAT00000025124]	ACAGACTTGAGCTTTCATTGCAGATGTGTGGTGCTAACCTTCTCCAACTCTTGTCCTCAT
259	A_69_P017488	0,04	0,00	up	1,19	1,19	0,25	NUDT1	nudix (nucleoside diphosphate linked moiety X)-type motif 1 [Source:HGNC Symbol;Acc:8048] [ENSECAT00000009517]	ACAGATACTGGTTTCCCCTCTTGCTTCAGAAGAAGAAATTCCATGGGTACTTCAAGTTCC
260	A_69_P047857	0,05	0,00	down	1,42	-1,42	-0,50		Uncharacterized protein [Source:UniProtKB/TrEMBL;Acc:F6TJN6] [ENSECAT00000024688]	AAGAAATTCCGAAGAAACTTGGATTAAGCTTTCAGTCACATCTGGCTGAATTGCAGAGAT
261	A_69_P113856	0,03	0,00	down	1,30	-1,30	-0,38		HL02015B1D08 Equine placenta cDNA Library Equus caballus cDNA clone HL02015B1D08, mRNA sequence [DN509322]	CTCGTGAACCTAATTGTAAACTTTCAGGTATTTTTGTACAAATAAGGGACTGATGTTCTG
262	A_69_P043706	0,03	0,00	up	1,25	1,25	0,33	NSMAF	neutral sphingomyelinase (N-SMase) activation associated factor [Source:HGNC Symbol;Acc:8017] [ENSECAT00000002019]	CTTGAAGGCAGATCAAGATAAGGCAGTGTTAGGGAGAAAACATTACATTTGTAAGGCAGA
263	A_69_P043571	0,05	0,00	down	1,13	-1,13	-0,17	VCPIP1	PREDICTED: Equus caballus valosin containing protein (p97)/p47 complex interacting protein 1 (VCPIP1), mRNA	TAAAGTCAATGTGATTCAGTAATGTTGCCGTCTGAGCCAACACAGCCAATTGTGTGCTCT

264	A_69_P063302	0,04	0,00	down	1,16	-1,16	-0,22	RHOBTB3	Rho-related BTB domain containing 3 [Source:HGNC Symbol;Acc:18757] [ENSECAT00000026534]	TCAGTGGAAGAGCGCAGTTTTGTTGAAAAGCACAGATGGCCGTCGAATATGTACTTGAAG
265	A_69_P061890	0,05	0,00	down	1,22	-1,22	-0,29	PPARGC1B	peroxisome proliferator-activated receptor gamma, coactivator 1 beta [Source:HGNC Symbol;Acc:30022] [ENSECAT00000021080]	TTCAGAAGAGGCCCTTCCTGCGTCAGTGAAAAACAAGTATGAAGCCATGGATTTTGACAG
266	A_69_P013146	0,04	0,00	up	1,23	1,23	0,30	GALNT12	UDP-N-acetyl-alpha-D-galactosamine:polypeptide N-acetylgalactosaminyltransferase 12 (GalNAc-T12) [Source:HGNC Symbol;Acc:19877] [ENSECAT00000017503]	ATGATGGTTCTTTATTTCACATACAGTCCAAGAAGTGTGTGCAGGCCGAAAGGAAGGCCT
267	A_69_P033292	0,05	0,00	up	1,91	1,91	0,93	KRT27	keratin 27 [Source:HGNC Symbol;Acc:30841] [ENSECAT00000011556]	TAGATCCTCGTGGCAAAGTTCTTTCCTCCAGAGTTCACACAATGGAAGAGAAATCTACCA
268	A_69_P124951	0,05	0,00	down	1,87	-1,87	-0,90			ATCCCTGCAGGCTGGGTCTTATACCACCTGGAGAATTACAAGCGCAGCTCTGCAGAATGA
269	A_69_P032537	0,04	0,00	up	1,21	1,21	0,28	CDC27	cell division cycle 27 [Source:HGNC Symbol;Acc:1728] [ENSECAT00000010389]	GTATCTCCCATAAACAAATGTGATGGGCATATTGGGACTCATGTGTAGGAATCAAATATT
270	A_69_P041493	0,04	0,00	up	1,53	1,53	0,61	STEAP3	STEAP family member 3, metalloreductase [Source:HGNC Symbol;Acc:24592] [ENSECAT00000006653]	GACACACACGTGCGTGTGATGTATATGTTCCTATATATTTCATATGTATAACAGGATTTG

271	A_69_P122512	0,04	0,00	up	1,18	1,18	0,23	TMEM243	transmembrane protein 243, mitochondrial [Source:HGNC Symbol;Acc:21707] [ENSECAT00000029037]	CCTGCATACTTATCTACTGGTATCGACAAGGAGACTTAGAACCGAAATTCAGAAATCTAA
272	A_69_P081046	0,03	0,00	down	1,13	-1,13	-0,18	ITFG1	integrin alpha FG-GAP repeat containing 1 [Source:HGNC Symbol;Acc:30697] [ENSECAT00000014564]	GTGCATTTGTGTTCCTTTGTGGAATGTGTTGCATGTTCTCAGGTGTTCATGTATTTATAA
273	A_69_P112211	0,05	0,00	down	1,19	-1,19	-0,26	TAPBPL	PREDICTED: Equus caballus TAP binding protein-like (TAPBPL), transcript variant X2, mRNA	TAACAAGCACTCCCTCTTTATCTAAGCCAACAATTAAAACTAACTGAGCGTCTCAAAAAA
274	A_69_P108995	0,03	0,00	down	1,80	-1,80	-0,85	FAM107A	CT02036A2F04 Equine Articular Cartilage cDNA Library Equus caballus cDNA clone CT02036A2F04, mRNA sequence [CX603734]	GATTGTTGCTTTCATCAGAGATGTAAATAATTGCCAATCTGCATTTCTTACCACACTGTT
275	A_69_P062466	0,05	0,00	down	1,20	-1,20	-0,26	FAM53C	family with sequence similarity 53, member C [Source:HGNC Symbol;Acc:1336] [ENSECAT00000001697]	GTGTGTGTTAAACATGAGGTCCTGCCTCTGTGGCTGTGTTTGAAAAAATAAAGTTTTATT
276	A_69_P012237	0,04	0,00	down	1,55	-1,55	-0,63	PLXDC2	plexin domain containing 2 [Source:HGNC Symbol;Acc:21013] [ENSECAT00000018697]	CTATGCTGAGGTTGAACCAGTGGGAGAGAAAGAAGGTTTCATTGTATCAGAGCAATGCTA
277	A_69_P059195	0,05	0,00	down	1,88	-1,88	-0,91	AAK1	AP2 associated kinase 1 [Source:HGNC Symbol;Acc:19679] [ENSECAT00000006573]	GATGCACCAGAAAAACTAATTGAGGGACTCAAATCTCCTGACACTTCTCTTCTGCTCCCT
278	A_69_P019476	0,05	0,00	down	2,07	-2,07	-1,05	RBFOX1	RNA binding protein, fox-1 homolog (C. elegans) 1 [Source:HGNC Symbol;Acc:18222] [ENSECAT00000013739]	CATCTTATGTGCATGCAGTAAGATCGGTTGGATATTAAACCATCAATAAAGTTTCACAAG

279	A_69_P117521	0,03	0,00	up	1,38	1,38	0,46	RNASEH 2C	PREDICTED: Equus caballus ribonuclease H2 subunit C-like (LOC100057522), mRNA [XM_001494343]	TGATGGTGATGGAAGAGAAGCATGA CTTCTCGGAGGGTTCGGAGAATAAC GAAGAGCAAG
280	A_69_P103264	0,04	0,00	down	1,59	-1,59	-0,67	GNAI1	PREDICTED: Equus caballus guanine nucleotide binding protein (G protein), alpha inhibiting activity polypeptide 1 (GNAI1), mRNA	GACGTACATATTGGTGACCATCATG GATGATTCCTGAACTTTATTCTGTGT AATTGTGTT
281	A_69_P113860	0,03	0,00	down	1,28	-1,28	-0,35	GRB10	PREDICTED: Equus caballus growth factor receptor bound protein 10 (GRB10), transcript variant X3, mRNA	TTGGCTCGTGAACCTAATTGTAAAC TTTCAGGTATTTTTGTACAAATAAGG GACTGATGT
282	A_69_P034596	0,04	0,00	up	1,13	1,13	0,17	COPRS	coordinator of PRMT5, differentiation stimulator [Source:HGNC Symbol;Acc:28848] [ENSECAT00000006253]	ATACCCCATTATTCCAAGATGGTCTT TGAAACGGGACAGTTTGATGATGCC GAGGACTGA
283	A_69_P049696	0,04	0,00	down	1,84	-1,84	-0,88	TPD52L1	tumor protein D52-like 1 [Source:HGNC Symbol;Acc:12006] [ENSECAT00000011106]	TCTCCTACTTTCAAATCATTTGAGGA GAGGGTTGAGACAACTGTCACAAGC CTTAAGACG
284	A_69_P095872	0,03	0,00	up	1,26	1,26	0,33	AP3M1	adaptor-related protein complex 3, mu 1 subunit [Source:HGNC Symbol;Acc:569] [ENSECAT00000008162]	TAACCTCTTGCCTAAAACAAGTGAA CTGGTCTCAGCCAGCAGGAGCTGT CTTAATCTGTA
285	A_69_P030547	0,04	0,00	down	1,20	-1,20	-0,26	KPNA1	karyopherin alpha 1 (importin alpha 5) [Source:HGNC Symbol;Acc:6394] [ENSECAT00000018698]	GGGGCACATATTAATTTGTATGGCT ACTGCTTCTCTTTGGTCTTACGTGTC TTAGGATTT
286	A_69_P000603	0,04	0,00	up	1,19	1,19	0,25	TMSB10	Equus caballus thymosin beta 10 (TMSB10), mRNA [NM_001081865]	ATCGGACTGCCAAATTCTTCGGTTT GCCCTGGGATATTATAGAAAATTATT TGTATGATT

287	A_69_P105382	0,04	0,00	down	2,45	-2,45	-1,29	NCALD	PREDICTED: Equus caballus neurocalcin delta (NCALD), transcript variant X2, mRNA [XM_005613237]	CATTGTCATTGCTGTGTTGAGGGACTTCTGGAAATGGAGTCCGTTCTGTCTGAAATCAAG
288	A_69_P060746	0,05	0,00	down	1,44	-1,44	-0,52		SRY (sex determining region Y)-box 11 [Source:HGNC Symbol;Acc:11191] [ENSECAT00000014050]	TCTGGTGGATAAGGATTTGGATTCGTTCAGCGAGGGCAGCCTGGGCTCCCACTTCGAGTT
289	A_69_P125606	0,05	0,00	up	1,29	1,29	0,36			TTTTAACTGGCCTTGGTTCTTATCTTCACATCCTTTTCAGCAACAAAGGCACTTCCTTCC
290	A_69_P095382	0,04	0,00	up	1,34	1,34	0,43	BICC1	bicaudal C homolog 1 (Drosophila) [Source:HGNC Symbol;Acc:19351] [ENSECAT00000014902]	TCTGGTATTAGGACCAAAGCATCGTATTCGCACCTGAACTTTGTGCCAAAAAGGAAGATA
291	A_69_P124646	0,04	0,00	down	1,26	-1,26	-0,34			GAGAAGCCCTATGAATGTAAAGAATGCAGCAAAGCCTTCACTGCTTCCAAATATCTTTGA
292	A_69_P027316	0,05	0,00	up	1,49	1,49	0,57	B4GALT1	UDP-Gal:betaGlcNAc beta 1,4-galactosyltransferase, polypeptide 1 [Source:HGNC Symbol;Acc:924] [ENSECAT00000012966]	TTTTCTACAACGTATTGGACGTACAGAGATACCCGTTGTATACCAAAGTCACAGTGGACA
293	A_69_P120480	0,05	0,00	up	1,75	1,75	0,81	MAPK14	PREDICTED: Equus caballus mitogen-activated protein kinase 14 (MAPK14), mRNA [XM_005604060]	TAGAACACGAGAAACTCACGGTGGATGAATGGAAGCAGCACATCTACAAGGAGATTGTGA
294	A_69_P038420	0,03	0,00	up	1,53	1,53	0,61	RPS18	ribosomal protein S18 [Source:HGNC Symbol;Acc:10401] [ENSECAT00000016886]	GCCGCACCGTGGGTGTGTCTAAGAAGAAATAAATCTGTAGGCTTTGTCTGTTAATAAATA
295	A_69_P063931	0,04	0,00	down	1,25	-1,25	-0,32	C14H1orf35	chromosome 1 open reading frame 35 [Source:HGNC Symbol;Acc:19032] [ENSECAT00000020840]	AAGACAAGGAGGCTGCTAAGCTGGGGCTCTCCGTGTTCACGCACCACCGTGTCGAGAGCA

296	A_69_P117661	0,05	0,00	down	1,94	-1,94	-0,96	RBFOX1		CACATCACGCAATCTGCAAACCCAGAAAATGTGTATATCTGTCTTTAGAAATTAGTGTTT
297	A_69_P000796	0,04	0,00	down	1,63	-1,63	-0,71	SLC2A5	Equus caballus solute carrier family 2 (facilitated glucose/fructose transporter), member 5 (SLC2A5), mRNA [NM_001081877]	GGAACAGGTCCCTGTGGAGATTTTAACTGAGTCAGAGATTATTGATCATAAATTATATTG
298	A_69_P057253	0,04	0,00	down	1,65	-1,65	-0,72	RAB6B	RAB6B, member RAS oncogene family [Source:HGNC Symbol;Acc:14902] [ENSECAT00000026634]	CAGGGATCACGTCACTCTTAACAGCTGTTACTTAAACAACTATTTTTTGGTTTGGTTGTA
299	A_69_P066437	0,04	0,00	down	2,07	-2,07	-1,05	GAREM	GRB2 associated, regulator of MAPK1 [Source:HGNC Symbol;Acc:26136] [ENSECAT00000012124]	TAATGGCTGGAGGCCCAAAATATAGCCAAATGACCCCAGCTAGTATTGAACGAAACTGAT
300	A_69_P064745	0,04	0,00	down	1,85	-1,85	-0,89	C8H22orf31	chromosome 22 open reading frame 31 [Source:HGNC Symbol;Acc:26931] [ENSECAT00000011961]	GAGCTCATGGGATCAGGATATTCTCTGCTAGATGTCTGAAAAATAAACACCACTTTGCAC
301	A_69_P000868	0,04	0,00	down	2,36	-2,36	-1,24	INOS	Equus caballus inducible nitric oxide synthase (INOS), mRNA [NM_001081769]	GGCACTTGGGTCTTCCTTGTATGTCTCCTTGATGGAGATATTTATATGAAATGCATTTTG
302	A_69_P101684	0,05	0,00	down	1,09	-1,09	-0,13	RNF10	ring finger protein 10 [Source:HGNC Symbol;Acc:10055] [ENSECAT00000013662]	TTATAATCGCAAACGTGAACCTTCCTACCCCAAAAATGAAAATTTCAGCAACCAGTTCCG
303	A_69_P008511	0,05	0,00	down	2,10	-2,10	-1,07	TGFB2	transforming growth factor, beta 2 [Source:HGNC Symbol;Acc:11768] [ENSECAT00000017826]	TTTCTAATATGATCGTCAAGTCTTGCAAATGCAGCTAAGATTCTTGGAAAAGTGGCCAGA

304	A_69_P095968	0,04	0,00	up	1,31	1,31	0,39	PLAC9	placenta-specific 9 [Source:HGNC Symbol;Acc:19255] [ENSECAT00000011851]	ACAGAGCCGTGCATTCTGGCCTTCCTTCCTCAGCTTTGTACACAATAAAAGCTACTTCTT
305	A_69_P112364	0,04	0,00	up	1,12	1,12	0,16	ENSA	PREDICTED: Equus caballus endosulfine alpha (ENSA), transcript variant 1, mRNA [XM_001490179]	CCCCACAGAGCCCAGGGGACTTTTTCAGTATTTGAAATAAAAAAGAAAAGAAGACCCACA
306	A_69_P073452	0,05	0,00	up	1,12	1,12	0,16	SFT2D2	SFT2 domain containing 2 [Source:HGNC Symbol;Acc:25140] [ENSECAT00000024115]	ATCTTCTGCATTTTGCAGTCTTTGGCCCTGACGTGGTATAGCCTTTCCTTCATACCGTTT
307	A_69_P000612	0,03	0,00	up	1,22	1,22	0,29	SOD2	Equus caballus superoxide dismutase 2, mitochondrial (SOD2), mRNA [NM_001082517]	TACATGGCTTGCAAAAAGTAAAGCATTATCGTTACACCGAGTGCATTAAGCAATTTCCAA
308	A_69_P008147	0,05	0,00	down	1,74	-1,74	-0,80	ZBTB18	zinc finger and BTB domain containing 18 [Source:HGNC Symbol;Acc:13030] [ENSECAT00000019934]	GACTGACATCTAGCTTTGACAATCATAGTATGTTTTATTTTCCTGAGGGGGGAATAACTT
309	A_69_P020681	0,04	0,00	down	1,70	-1,70	-0,77	TMCC3	Uncharacterized protein [Source:UniProtKB/TrEMBL;Acc:F7AJN5] [ENSECAT00000012141]	TGACTCTTCTTGCAATATTTTGTAAAAACTGGGACCATATTCTGTGTGCCATAGAAAGGA
310	A_69_P011116	0,04	0,00	up	1,44	1,44	0,52	RNASEH2C	ribonuclease H2, subunit C [Source:HGNC Symbol;Acc:24116] [ENSECAT00000018200]	AGGAAGTGTCGGTGCCACCCGGCCTCGTGGGATACGTGATGGTGATGGAAGAGAAGCATG
311	A_69_P058276	0,04	0,00	down	3,70	-3,70	-1,89	MAL	Uncharacterized protein [Source:UniProtKB/TrEMBL;Acc:F6Y3W5] [ENSECAT00000017968]	GCCACTCTGCTGTACGTGGTCCATGCGGTATTTTCTTTAATCAGATGGAAGTCTTCGTAA

312	A_69_P095889	0,03	0,00	down	1,88	-1,88	-0,91	DUSP13	dual specificity phosphatase 13 [Source:HGNC Symbol;Acc:19681] [ENSECAT00000000110]	GACCCAGTCTAGAGATTCTTTATGCAAAAGAGAGTTCAGTCTGTCTCTATAATAAAAGGT
313	A_69_P092431	0,04	0,00	up	1,24	1,24	0,31	PAK3	p21 protein (Cdc42/Rac)-activated kinase 3 [Source:HGNC Symbol;Acc:8592] [ENSECAT00000017283]	CATTTTTAAAATTAGCCAAGCCTCTCTCCAGCCTGACTCCTCTGATTATCGCTGCAAAGG
314	A_69_P099001	0,04	0,00	up	1,15	1,15	0,20	GCHFR	GTP cyclohydrolase I feedback regulator [Source:HGNC Symbol;Acc:4194] [ENSECAT00000007126]	AAGTGTCCTAGGAAACAACTTTTGTGAGTACTACGTCAATGACCCTCCTCGCATAGTCCT
315	A_69_P012238	0,04	0,00	down	1,54	-1,54	-0,62	PLXDC2	plexin domain containing 2 [Source:HGNC Symbol;Acc:21013] [ENSECAT00000018697]	CCTATGCTGAGGTTGAACCAGTGGGAGAGAAAGAAGGTTTCATTGTATCAGAGCAATGCT
316	A_69_P087936	0,05	0,00	down	1,66	-1,66	-0,73	FAM213B	family with sequence similarity 213, member B [Source:HGNC Symbol;Acc:28390] [ENSECAT00000008442]	TGGGAGTCAGATGTGAAGACTCGTTCTGGAATCTCTGGGCCGGGATGCCGAATTCTGCCT
317	A_69_P057255	0,03	0,00	down	1,72	-1,72	-0,78	RAB6B	RAB6B, member RAS oncogene family [Source:HGNC Symbol;Acc:14902] [ENSECAT00000026634]	ATCAGGGATCACGTCACTCTTAACAGCTGTTACTTAAACAACTATTTTTGGTTTGGTTG
318	A_69_P018308	0,04	0,00	up	1,12	1,12	0,17	VKORC1L1	vitamin K epoxide reductase complex, subunit 1-like 1 [Source:HGNC Symbol;Acc:21492] [ENSECAT00000011515]	ACTTCATTCTTCTTATCATCAACTACAAACGACTAGTTTACTTAAATGAGGCCTGGAAAC
319	A_69_P094831	0,04	0,00	down	2,10	-2,10	-1,07	CRTAC1	cartilage acidic protein 1 [Source:HGNC Symbol;Acc:14882] [ENSECAT00000007808]	ATATGGAAGCTACAGGTGCCGGACCAACAAGAGATGCAGTCGGGGCTATGAGCCCAACGA

320	A_69_P055850	0,05	0,00	up	1,14	1,14	0,19	NPRL2	nitrogen permease regulator-like 2 (S. cerevisiae) [Source:HGNC Symbol;Acc:24969] [ENSECAT00000024287]	GACTAGTCCATATTGTCTCAAGGTTGACCCTCACCTCTGTAAATAAAGTCATCCATTTCA
321	A_69_P115816	0,05	0,00	up	5,02	5,02	2,33		APL1_3_H09.g1_A008 Liver (APL1) Equus caballus cDNA, mRNA sequence [BM780438]	TACAACCTTCCTGTTTCTATGAAATCTGTGAATTTGAATAAAAGTTGTGACTCTAAAAAA
322	A_69_P075972	0,04	0,00	up	1,14	1,14	0,19	TARS2	threonyl-tRNA synthetase 2, mitochondrial (putative) [Source:HGNC Symbol;Acc:30740] [ENSECAT00000015021]	ACAGAACACCAGGGTGCCAAATGCTGAAGAGATTTTCTGAGCTTCTGTATATAGATGTGA
323	A_69_P062196	0,05	0,00	down	1,99	-1,99	-0,99	PCDHB14	protocadherin beta 14 [Source:HGNC Symbol;Acc:8685] [ENSECAT00000001099]	GGTGGGAATACGGGGGAAAATGAGAACTTTAGGAATAGCTTTGGATTCAACATTCAATAA
324	A_69_P003436	0,04	0,00	up	2,81	2,81	1,49	LOC100630543	Metallothionein [Source:UniProtKB/TrEMBL;Acc:F7DM22] [ENSECAT00000015995]	CCCAATTGCTGCATCCCCTTTTCTATGAAATATGTGAATGACAATAAAAGTTATTGACCT
325	A_69_P004686	0,05	0,00	up	1,18	1,18	0,24	SMAD5	Equus caballus SMAD family member 5 (SMAD5), mRNA [NM_001163888]	ATTCCTCAGATTATGCCCAGTATATCCAGTAGAGATGTTCAGCCTGTTGCCTATGAAGAG
326	A_69_P019207	0,05	0,00	down	1,74	-1,74	-0,80	SYT17	synaptotagmin XVII [Source:HGNC Symbol;Acc:24119] [ENSECAT00000009185]	TCCTTTCTACAACGAGTCCTTCAGCTTCAAAGTTCCCCAAGAAGAACTGGAAAATGCCAG
327	A_69_P001293	0,04	0,00	up	1,03	1,03	0,04	ECATH-3	Equus caballus myeloid cathelicidin 3 (ECATH-3), mRNA [NM_001081930]	GAGGAAGATGGGCACAGATGTTAGCTCAGGGCTAATCTTCCTCAAATAAATAAATAAATA
328	A_69_P108561	0,04	0,00	down	1,10	-1,10	-0,13	ZFP91	PREDICTED: Acinonyx jubatus ZFP91 zinc finger protein (ZFP91), mRNA	CTTTGATGGGAGAGACAGGTAGTTACCTGAATCTAGGTTGAAAAGGTTATGTAAAAAGAA

329	A_69_P065091	0,04	0,00	up	1,19	1,19	0,26	SRSF9	serine/arginine-rich splicing factor 9 [Source:HGNC Symbol;Acc:10791] [ENSECAT00000009312]	GAGATCAGCAGTTAACTGTGACTGAGTCGGCCCATTCTGTTTAGAAATATATTTTAAACA
330	A_69_P116253	0,05	0,00	down	2,06	-2,06	-1,04		LeukoS2_3_C09.b1_A024 Stimulated peripheral blood leukocytes S2 Equus caballus cDNA clone LeukoS2_3_C09_A024 3', mRNA sequence [CD469289]	AAGAAGATTTTTAAAGTTTGAGAGTGGAAATAAATGGAAGCCCTGAGACCCTTCCAGAAG
331	A_69_P081270	0,05	0,00	up	7,14	7,14	2,84	LOC100630858	Metallothionein [Source:UniProtKB/TrEMBL;Acc:F6VKN2] [ENSECAT00000001640]	CACCCCTGACTTGATTGCTACATTCCTTTTTCTATGAAATGTTTGAGTGGCAATAAACTC
332	A_69_P072567	0,04	0,00	up	1,07	1,07	0,10	DENND5A	DENN/MADD domain containing 5A [Source:HGNC Symbol;Acc:19344] [ENSECAT00000013955]	GCAGGAGACGGAGAGGAAATGTACACTCACTGTAAAAAGAAAACTAGAGGATTTTTGGAA
333	A_69_P028266	0,05	0,00	up	1,48	1,48	0,56	LPAR2	lysophosphatidic acid receptor 2 [Source:HGNC Symbol;Acc:3168] [ENSECAT00000009394]	AACTCACTGGTCAACGCTGTAGTGTACTCGTGCCGTGATGCTGAGATGCGTCGCACCTTC
334	A_69_P111261	0,04	0,00	down	1,44	-1,44	-0,53	JADE1	PREDICTED: Equus caballus jade family PHD finger 1 (JADE1), mRNA	ACTAGTGGCATATGTCGGGGAATCTGGGCTTCCAACATGAACGGATTCCTTAAGAAAAAA
335	A_69_P083138	0,04	0,00	down	2,08	-2,08	-1,06	SCD5	stearoyl-CoA desaturase 5 [Source:HGNC Symbol;Acc:21088] [ENSECAT00000015537]	TGCGAGTGAATTTGGCCTAAATTTCAACCCAACCACCTGGTTCATTGACTTCATGTGCTG
336	A_69_P103632	0,05	0,00	down	1,67	-1,67	-0,74	SLC15A4	PREDICTED: Equus caballus solute carrier family 15 (oligopeptide transporter), member 4 (SLC15A4), mRNA [XM_005615190]	TGAACTTGGACTGTTGGACGGCCTCTGACTTCTGTTTCCTTATTTTACATGTATGTGGGG

337	A_69_P003017	0,05	0,00	up	1,19	1,19	0,25	SMAD5	Equus caballus SMAD family member 5 (SMAD5), mRNA [NM_001163888]	ACTCTCAAAAGTGTTGTGAACGATGCAGAGTAAGTATGATGCCCCAGTTCAAAAGTTTGG
338	A_69_P114808	0,05	0,00	down	1,59	-1,59	-0,67		HL02021B2B03 Equine placenta cDNA Library Equus caballus cDNA clone HL02021B2B03, mRNA sequence [DN511039]	TGGAGCTTGTTCTTTTGCTGGATGAGCACTCAAATTTTCTTTCCTGCAAAGATTGTTTCA
339	A_69_P065077	0,05	0,00	down	1,31	-1,31	-0,39	RNF10	ring finger protein 10 [Source:HGNC Symbol;Acc:10055] [ENSECAT00000013662]	GGGGGTTTCCACAGTGTGAGGGGGAACCAAGAAAATTTTAAATACAGTGTATTTTCCAGC
340	A_69_P107044	0,04	0,00	down	1,97	-1,97	-0,98	FBLN7	PREDICTED: Equus caballus fibulin 7 (FBLN7), mRNA [XM_001916548]	CATTCCATAGAACCATATAGAATGGTTTTCTACAAAATGCTCCATTCTGAGGACATACGA
341	A_69_P004149	0,04	0,00	up	1,17	1,17	0,22	SOD2	Equus caballus superoxide dismutase 2, mitochondrial (SOD2), mRNA [NM_001082517]	CATTTGGAATGTGATCAACTGGGAGAATGTATCTGAAAGATACATGGCTTGCAAAAAGTA
342	A_69_P026867	0,04	0,00	up	1,41	1,41	0,49	SMARCA2	SWI/SNF related, matrix associated, actin dependent regulator of chromatin, subfamily a, member 2 [Source:HGNC Symbol;Acc:11098] [ENSECAT00000026315]	ATCTCCTATGTTACCAATGTGTATCGTCTCCTTCTCCCTAAAGTGTACTTAATCTTTGCT
343	A_69_P012301	0,04	0,00	down	1,55	-1,55	-0,63	PTPLA	protein tyrosine phosphatase-like (proline instead of catalytic arginine), member A [Source:HGNC Symbol;Acc:9639] [ENSECAT00000021501]	CACGGTGCTTTTTCCAGAACAATCAGGATCACTTGAGTCCAAGTTTTAATAACAAGAATA

344	A_69_P045208	0,04	0,00	up	1,12	1,12	0,17		chromosome 8 open reading frame 33 [Source:HGNC Symbol;Acc:26104] [ENSECAT00000012375]	TCCAGTATTAGGTGTAGTAGCTGTTGGTAATGAGGATTAGTGGGAAAGCTGCATAGTTGT
345	A_69_P077890	0,05	0,00	up	1,11	1,11	0,16	KMT2E	lysine (K)-specific methyltransferase 2E [Source:HGNC Symbol;Acc:18541] [ENSECAT00000018531]	ACAGATTCCAATTCACAGAGCACAGGTGCCACCAACATTTCAGAACAATTACCATGGTTC
346	A_69_P012240	0,05	0,00	down	1,50	-1,50	-0,59	PLXDC2	plexin domain containing 2 [Source:HGNC Symbol;Acc:21013] [ENSECAT00000018697]	CTGCCTATGCTGAGGTTGAACCAGTGGGAGAGAAAGAAGGTTTCATTGTATCAGAGCAAT
347	A_69_P117884	0,03	0,00	down	6,56	-6,56	-2,71			AGCCCATTGAAGCCATTTACCTGACCCTAGAGCTTTTATCATAATTAAACATGATTATGG
348	A_69_P119106	0,05	0,00	down	2,22	-2,22	-1,15	MYF5	myogenic factor 5 [Source:HGNC Symbol;Acc:7565] [ENSECAT00000021416]	TGTGCTATGAACTAAAAATCTAGTCTAGATCAGTTCTGCCAGGAGGGCCTATTACACAGG
349	A_69_P102589	0,05	0,00	up	1,79	1,79	0,84	ABCA1	PREDICTED: Equus caballus ATP-binding cassette, sub-family A (ABC1), member 1 (ABCA1), mRNA [XM_001493790]	CAGAGATTCCTGCCTTTGCTTCTTTTGGACACTCTGGAAACCTTATTAACAACTGTGAAT
350	A_69_P062961	0,04	0,00	up	1,99	1,99	1,00	PRR16	proline rich 16 [Source:HGNC Symbol;Acc:29654] [ENSECAT00000017745]	AATCCTACGTTCAGCAAAGTGGCATAAAAATCACCTGGTAAGTATGCAGTATGGTTGTAT
351	A_69_P013616	0,05	0,00	down	1,33	-1,33	-0,41	ALAD	aminolevulinate dehydratase [Source:HGNC Symbol;Acc:395] [ENSECAT00000016415]	ACACACCTCTGCTCTTGCAGTGGCTGAAGGAGTGATGGAGAGAGTGTGGAAGCCCCAAGA

352	A_69_P057506	0,04	0,00	up	1,56	1,56	0,64	PCOLCE2	procollagen C-endopeptidase enhancer 2 [Source:HGNC Symbol;Acc:8739] [ENSECAT00000021960]	CCAAGTGGGTGAAGATGGGCGAGGCAAAATCATGCCAAACAGCTTTATCATGATGTTCAA
353	A_69_P108956	0,05	0,00	up	1,23	1,23	0,30		CT02032A1A11 Equine Articular Cartilage cDNA Library Equus caballus cDNA clone CT02032A1A11, mRNA sequence [CX602199]	GGCAGCTGCACTGGTTTACTTTAAAATGTACAGATCTTCCCATTAAATTCATGATAGATT
354	A_69_P081268	0,05	0,00	up	7,18	7,18	2,84	LOC100630858	Metallothionein [Source:UniProtKB/TrEMBL;Acc:F6VKN2] [ENSECAT00000001640]	CCCCTGACTTGATTGCTACATTCCTTTTTCTATGAAATGTTTGAGTGGCAATAAACTCAT
355	A_69_P102588	0,04	0,00	up	1,71	1,71	0,77	ABCA1	PREDICTED: Equus caballus ATP-binding cassette, sub-family A (ABC1), member 1 (ABCA1), mRNA [XM_001493790]	AGAGATTCCTGCCTTTGCTTCTTTTGGACACTCTGGAAACCTTATTAACAACTGTGAATA
356	A_69_P083006	0,05	0,00	down	1,26	-1,26	-0,34	HSD17B11	hydroxysteroid (17-beta) dehydrogenase 11 [Source:HGNC Symbol;Acc:22960] [ENSECAT00000008370]	CCTGAGCGTTTCTTGGCAATTTTAAAACGAAAGATGGATATAAAGTTTGATGCAGTGATT
357	A_69_P033766	0,03	0,00	up	1,28	1,28	0,36	NXPH3	neurexophilin 3 [Source:HGNC Symbol;Acc:8077] [ENSECAT00000018278]	GCACAGACTATAGGCTGGTCCAGAAGGTGTGCCCAGATTACAACTACCACAGTGATACCC
358	A_69_P071112	0,04	0,00	up	1,24	1,24	0,31	RPS3	Uncharacterized protein [Source:UniProtKB/TrEMBL;Acc:F7BA94] [ENSECAT00000014732]	TGGCAGCTGGATCTGGAGTCTGGATGTTGCTCTATAAAGACCTTTAATAAAATGTTTTTA
359	A_69_P086357	0,04	0,00	up	1,14	1,14	0,19	EIF3I	eukaryotic translation initiation factor 3, subunit I [Source:HGNC Symbol;Acc:3272] [ENSECAT00000014469]	CGGAAAGTTTTGGACTATGAGAAGTAAATCAGTTTGGGAATAAATTGTTACTGGTCAGAT

360	A_69_P114726	0,05	0,00	up	1,45	1,45	0,54	POC1A	PREDICTED: Equus caballus POC1 centriolar protein A (POC1A), mRNA	TGTGGGACTTGGGTAAATAAATAAAGGGGATTCCACTCTGTGGGAACCACATCCAAAAAA
361	A_69_P113586	0,03	0,00	down	1,60	-1,60	-0,68	LAMA5	PREDICTED: Equus caballus laminin, alpha 5 (LAMA5), partial mRNA	AGATAAATATACACGAACTTCCTCTGTGAAACGGGCTGAGGGCAGTGTTGTCAGTAAGCT
362	A_69_P054366	0,03	0,00	up	1,17	1,17	0,23	METTL1	methyltransferase like 1 [Source:HGNC Symbol;Acc:7030] [ENSECAT00000009295]	AAGTGCTACGCAATGGAGGAAAGAATTTCCCAGCCATCTTCCGAAGAATACAGGATCTCA
363	A_69_P114763	0,04	0,00	up	1,16	1,16	0,21	ANKRD13A	Select seq ref|XM_001496710.5| PREDICTED: Equus caballus ankyrin repeat domain 13A (ANKRD13A), transcript variant X2, mRNA	AAGTCAGTTAAAAAGGGACTGAGCATTTTGTAAACCTGTCCCGGTGTTTTTCATGTTATG
364	A_69_P022611	0,04	0,00	down	1,36	-1,36	-0,44	DCAF5	DDB1 and CUL4 associated factor 5 [Source:HGNC Symbol;Acc:20224] [ENSECAT00000010943]	CACCGAGTTGAATTGGAAGATACAGATTCAGAGAATTCCTCCTCAGAGAAGAAATTAAAA
365	A_69_P103846	0,05	0,00	down	1,85	-1,85	-0,89	TPD52	TSA: Equus caballus contig27111.EqcaPBMC mRNA sequence. [JL640875]	AGAGCTTCTGTTAGCACTTTAGATACAAGTGAAACTACGCTCTATTACATGTGAAAAGCT
366	A_69_P068801	0,05	0,00	down	2,06	-2,06	-1,04	MCAM	melanoma cell adhesion molecule [Source:HGNC Symbol;Acc:6934] [ENSECAT00000013706]	ATTTGTAGTTGAAGTTAAGTCAGATAAACTCCCAGAAGAGATGGCCTCCTACAGGGCAG
367	A_69_P012851	0,04	0,00	up	1,17	1,17	0,23	RGP1	RGP1 retrograde golgi transport homolog (S. cerevisiae) [Source:HGNC Symbol;Acc:21965] [ENSECAT00000010431]	CATTAATTTATTCAGAATAAACTGGCAGCTGCTAGTGGTTTCCCTGGAAGTGGCAGCAGC

368	A_69_P032002	0,05	0,00	down	1,31	-1,31	-0,39	HN1	hematological and neurological expressed 1 [Source:HGNC Symbol;Acc:14569] [ENSECAT00000020042]	CTGCACAACTTGAGCCTGACTGTACATCTTTTGGATTTGTTTCATTAAAAAAGAAGCACT
369	A_69_P043722	0,04	0,00	down	1,84	-1,84	-0,88	FAM110B	family with sequence similarity 110, member B [Source:HGNC Symbol;Acc:28587] [ENSECAT00000004724]	CATCAAGTGGTTATATAGCATCAAACAAGCTAGAGAGTCACAGAAGGTCTCCCACGTGTA
370	A_69_P110531	0,03	0,00	down	1,42	-1,42	-0,51		CT02034A1E10 Equine Articular Cartilage cDNA Library Equus caballus cDNA clone CT02034A1E10, mRNA sequence [CX602929]	CCGTTCTCTCCCTTAATGTTTACGTTGGTTTGCAATACATAAAGAAACAATGACAACAGA
371	A_69_P105417	0,04	0,00	down	1,22	-1,22	-0,28	CSNK1E	PREDICTED: Equus caballus casein kinase 1, epsilon (CSNK1E), transcript variant X5, mRNA	GAAGAACTTTGGGGGGAATTTTGTACTTTTTTCCTGTGTAAATATTGGACTTTTTTGAGC
372	A_69_P031116	0,05	0,00	down	1,84	-1,84	-0,88	ST3GAL6	ST3 beta-galactoside alpha-2,3-sialyltransferase 6 [Source:HGNC Symbol;Acc:18080] [ENSECAT00000010985]	GTTGCTGCCTGGTCATTCATAACCACCGGCTTAATTTCTGTGAATATATTTAATTTATGA
373	A_69_P019816	0,05	0,00	up	1,29	1,29	0,36	AMDHD2	N-acetylglucosamine-6-phosphate deacetylase [Source:UniProtKB/TrEMBL;Acc:F6PZ12] [ENSECAT00000013192]	AACGCCTTCCAGGACGTGCTGGACACCTATGGTTCCCTGGACAACGTCTGCATTGTGACA
374	A_69_P057254	0,03	0,00	down	1,63	-1,63	-0,70	RAB6B	RAB6B, member RAS oncogene family [Source:HGNC Symbol;Acc:14902] [ENSECAT00000026634]	TCAGGGATCACGTCACTCTTAACAGCTGTTACTTAAACAACTATTTTTTGGTTTGGTTGT

375	A_69_P017487	0,03	0,00	up	1,21	1,21	0,27	NUDT1	nudix (nucleoside diphosphate linked moiety X)-type motif 1 [Source:HGNC Symbol;Acc:8048] [ENSECAT00000009517]	CAGATACTGGTTTCCCCTCTTGCTTCAGAAGAAGAAATTCCATGGGTACTTCAAGTTCCG
376	A_69_P117831	0,04	0,00	down	1,27	-1,27	-0,34	CALM1	calmodulin 1 (phosphorylase kinase, delta) [Source:HGNC Symbol;Acc:1442] [ENSECAT00000009414]	GAAAAATGAAAGATACAGACAGCGAAGAAGAAATCCGTGAGGCATTCCGAGTCTTTGACA
377	A_69_P057251	0,05	0,00	down	1,65	-1,65	-0,73	RAB6B	RAB6B, member RAS oncogene family [Source:HGNC Symbol;Acc:14902] [ENSECAT00000026634]	GGGATCACGTCACTCTTAACAGCTGTTACTTAAACAACTATTTTTTGGTTTGGTTGTAAT
378	A_69_P114730	0,04	0,00	up	1,46	1,46	0,55	POC1A	HL02021A1F07 Equine placenta cDNA Library Equus caballus cDNA clone HL02021A1F07, mRNA sequence [DN510872]	GTACTGTGGGACTTGGGTAAATAAATAAAGGGGATTCCACTCTGTGGGAACCACATCCAA
379	A_69_P095969	0,04	0,00	up	1,32	1,32	0,40	PLAC9	placenta-specific 9 [Source:HGNC Symbol;Acc:19255] [ENSECAT00000011851]	AACAGAGCCGTGCATTCTGGCCTTCCTTCCTCAGCTTTGTACACAATAAAAGCTACTTCT
380	A_69_P066921	0,03	0,00	down	1,15	-1,15	-0,21	PIGN	phosphatidylinositol glycan anchor biosynthesis, class N [Source:HGNC Symbol;Acc:8967] [ENSECAT00000001039]	ACAAGCATCAGCCACTACGTGATTGTCATGTCCATGACCATGGTTCTGGTCTTTCTCAAT
381	A_69_P028466	0,04	0,00	down	1,26	-1,26	-0,33	KIF2A	kinesin heavy chain member 2A [Source:HGNC Symbol;Acc:6318] [ENSECAT00000021029]	GTGGACATAGCTGCTGGACCATTCCATCTTATATGTAAAGAAATCTGGAATTATGATTTT

382	A_69_P038418	0,05	0,00	up	1,42	1,42	0,51	RPS18	ribosomal protein S18 [Source:HGNC Symbol;Acc:10401] [ENSECAT00000016886]	CGCACCGTGGGTGTGTCTAAGAAGAAATAAATCTGTAGGCTTTGTCTGTTAATAAATAGT
383	A_69_P066439	0,04	0,00	down	2,10	-2,10	-1,07	GAREM	GRB2 associated, regulator of MAPK1 [Source:HGNC Symbol;Acc:26136] [ENSECAT00000012124]	AAATTGAGCAAACTGCAGGTGAAGAAAATCATGCAATTCATTAATGGCTGGAGGCCCAAA
384	A_69_P065110	0,05	0,00	down	1,24	-1,24	-0,31	LOC100053420	Cytochrome c oxidase subunit 6A, mitochondrial [Source:UniProtKB/TrEMBL;Acc:F6RVC7] [ENSECAT00000013033]	TCCTGACCTTCCGTCCCTTTGCTTATGGCAGGAGATGGCTTAAATAAATAACTTAGATTA
385	A_69_P000961	0,03	0,00	down	1,66	-1,66	-0,73	FST	Equus caballus follistatin (FST), mRNA [NM_001081811]	GCTAGTCTGCTGTACCTTTTAAATCAGTTCTTTTTCAACCAGTGTGTCACTAAAAGTTAT
386	A_69_P123562	0,04	0,00	down	1,46	-1,46	-0,54	LYRM9	Uncharacterized protein [Source:UniProtKB/TrEMBL;Acc:F6Z674] [ENSECAT00000010046]	TCCAGCAGATTATTAAAAGAGCCATAGAAGATGCCGACTGGATCATGAACAAATATAAAA
387	A_69_P119956	0,04	0,00	down	1,14	-1,14	-0,19			TAAAGAAATAAAAGGGGCTCTGAAAAGTGTGCTCAAGAGAAAGTGCCCCTCCTTGCCCAA
388	A_69_P057507	0,04	0,00	up	1,62	1,62	0,70	PCOLCE2	procollagen C-endopeptidase enhancer 2 [Source:HGNC Symbol;Acc:8739] [ENSECAT00000021960]	CAATGTCCTCTCCTCAGAAGAGGTCTAAATTACATTATTATGGCCAAGTGGGTGAAGAT
389	A_69_P082419	0,05	0,00	up	1,06	1,06	0,08	COX4I1	cytochrome c oxidase subunit IV isoform 1 [Source:HGNC Symbol;Acc:2265] [ENSECAT00000012285]	AGCATATATTTACTGGAAACCGTCACGTCGTGTACCAGTGCTAATAAATGAGCAGTTTAC

390	A_69_P043686	0,03	0,00	down	1,40	-1,40	-0,48	CHD7	chromodomain helicase DNA binding protein 7 [Source:HGNC Symbol;Acc:20626] [ENSECAT00000003445]	TTGCTGCCAACTGTAGTAATGATGCTTTTAATAAAAGTGACCCATGATATGCAGAGATGT
391	A_69_P105926	0,05	0,00	up	1,12	1,12	0,17	CPNE3	PREDICTED: Equus caballus copine III (CPNE3), mRNA	CCCGATAACACGTGTGTAAGTTACTAATATGTGAATTGTGTTAAATGTATCTTCCGTTTG
392	A_69_P038419	0,03	0,00	up	1,47	1,47	0,55	RPS18	ribosomal protein S18 [Source:HGNC Symbol;Acc:10401] [ENSECAT00000016886]	CCGCACCGTGGGTGTGTCTAAGAAGAAATAAATCTGTAGGCTTTGTCTGTTAATAAATAG
393	A_69_P107033	0,05	0,00	down	1,15	-1,15	-0,20	FAM20B	PREDICTED: Equus caballus family with sequence similarity 20, member B (FAM20B), transcript variant X1, mRNA [XM_001916737]	ATGTGTTTATTCCAGAGTGGGGAAATATGGGTACAGAGGTTTCATCCTAAAATTGTTCTT
394	A_69_P050961	0,04	0,00	up	1,15	1,15	0,21	ILKAP	integrin-linked kinase-associated serine/threonine phosphatase [Source:HGNC Symbol;Acc:15566] [ENSECAT00000026603]	TTCTGTGTGTCTCGTGTACTCCTGTGGGACTCCCATGGTTGTAAATAAAGGTTTCTTTCT
395	A_69_P081401	0,04	0,00	down	1,80	-1,80	-0,85	MMP15	matrix metallopeptidase 15 (membrane-inserted) [Source:HGNC Symbol;Acc:7161] [ENSECAT00000000336]	TACTGGAAGTTCGACAACGAGCGTCTGCGAATGGAGCCTGGCTACCCCAAATCCATCCTG
396	A_69_P104923	0,04	0,00	down	1,31	-1,31	-0,39	MAP1LC3B	TSA: Equus caballus contig01082.EqcaPBMC mRNA sequence. [JL617449]	AGAAATTCTGCTTTTTAAAAAACAAAAATACCATGCTGAGAGGGGGACTTACATCCCACA
397	A_69_P066436	0,03	0,00	down	2,01	-2,01	-1,01	GAREM	GRB2 associated, regulator of MAPK1 [Source:HGNC Symbol;Acc:26136] [ENSECAT00000012124]	TATAGCCAAATGACCCCAGCTAGTATTGAACGAAACTGATACATGTGTGTGCTAGAAGGG

398	A_69_P009086	0,04	0,00	up	1,33	1,33	0,42	ALKBH3	alkB, alkylation repair homolog 3 (E. coli) [Source:HGNC Symbol;Acc:30141] [ENSECAT00000011166]	AACTCACTGCTCTGCAATCTTTACCGAAATGAGAAGGACAGTGTGGACTGGCATAGTGAT
399	A_69_P125825	0,05	0,00	down	1,25	-1,25	-0,32	PMAIP1	PREDICTED: Equus caballus phorbol-12-myristate-13-acetate-induced protein 1 (PMAIP1), mRNA [XM_003365575]	GACAAACTGAATTTCCGGCAGAAACTTCTGAATCTCATAGCCAAACTCTTCCGCTCAGGA
400	A_69_P074260	0,03	0,00	down	1,23	-1,23	-0,30	KCNH1	potassium voltage-gated channel, subfamily H (eag-related), member 1 [Source:HGNC Symbol;Acc:6250] [ENSECAT00000000575]	ACTCAGGATATTAACTTCCAGAAGATCCTCTCAGTCTCCCCAGGAGCTGTTTGAAATATC
401	A_69_P030281	0,04	0,00	down	1,70	-1,70	-0,76	BDH1	3-hydroxybutyrate dehydrogenase, type 1 [Source:HGNC Symbol;Acc:1027] [ENSECAT00000005089]	CGGATGCAGATCATGACCCACTTTCCCGGAGCAATCTCTGACAAGATCTACATACACTGA
402	A_69_P096171	0,05	0,00	up	1,21	1,21	0,27	MTR	5-methyltetrahydrofolate-homocysteine methyltransferase [Source:HGNC Symbol;Acc:7468] [ENSECAT00000023851]	TATGCTTTGAGGAAGAACATGTCTGTGGCTGAGGTTGAGAAATGGCTTGGACCCATTTTG
403	A_69_P003437	0,04	0,00	up	2,80	2,80	1,48	LOC100630543	Metallothionein [Source:UniProtKB/TrEMBL;Acc:F7DM22] [ENSECAT00000015995]	ACCCAATTGCTGCATCCCCTTTTCTATGAAATATGTGAATGACAATAAAAGTTATTGACC
404	A_69_P063341	0,04	0,00	down	1,37	-1,37	-0,46	ANKRD32	ankyrin repeat domain 32 [Source:HGNC Symbol;Acc:25408] [ENSECAT00000016750]	GCAAAACTGTGGTATAGACTCCTGTCTTTTTCCAGTGTATAGAATTTGACTCAAAAGTAA

405	A_69_P105859	0,05	0,00	up	1,16	1,16	0,21	GNA12	PREDICTED: Equus caballus guanine nucleotide binding protein (G protein) alpha 12 (GNA12), transcript variant X3, mRNA [XM_005598542]	TCCCCTCTGTGTATTACTCTACTAAAAAAGTCTGTCTATTGTGTTTAGGACTTTTCCTAG
406	A_69_P102192	0,04	0,00	up	5,21	5,21	2,38		APL1_9_G09.g1_A008 Liver (APL1) Equus caballus cDNA, mRNA sequence [BM780851]	ACAACTTGCCTGTTTCTATGAGATCTGTGAATTCCAAATAAACGTGTGTTGACTCTAAAA
407	A_69_P111986	0,05	0,00	down	1,06	-1,06	-0,08	LAMP2	Select seq ref|XM_014729148.1| PREDICTED: Equus caballus lysosomal-associated membrane protein 2 (LAMP2), transcript variant X5, mRNA	CAGCAAAAAAGAAGAGGATTGGTGGCAGATGTTAGCTCAGGGCTAATCTTCCTCAAAAAA
408	A_69_P114570	0,04	0,00	up	1,21	1,21	0,27	FAM134A	Select seq ref|XM_014740307.1| PREDICTED: Equus caballus family with sequence similarity 134 member A (FAM134A), partial mRNA	CTGACAGTGTGATATGTTTAGTGAGACTTAGCATGTGTGAATAAAGTGTATGCAGGAGGA
409	A_69_P003576	0,04	0,00	down	1,21	-1,21	-0,28	SMAD1	Equus caballus SMAD family member 1 (SMAD1), mRNA [NM_001163882]	GCTCTCGTACATTGAGTACTTTTATTCCAAAACTAGTGGGTTTTCTCTACTGGAAATTTT
410	A_69_P080911	0,04	0,00	down	1,36	-1,36	-0,45		Uncharacterized protein [Source:UniProtKB/TrEMBL;Acc:F7DHT1] [ENSECAT00000007776]	AGTTTCCCAGACAAGCAAAACCTGAAGGAGTTTACCACCAAGAAACCAAGCTTACAAGAA
411	A_69_P085920	0,04	0,00	down	1,40	-1,40	-0,49	MACF1	microtubule-actin crosslinking factor 1 [Source:HGNC Symbol;Acc:13664] [ENSECAT00000017611]	GCGCGGTGCAGGGTTGTAAACCTGCTTTATCTTTTAGGATTATTCCTAAATGCATCTTCT

412	A_69_P097521	0,05	0,00	down	1,26	-1,26	-0,33	DNAJA4	DnaJ (Hsp40) homolog, subfamily A, member 4 [Source:HGNC Symbol;Acc:14885] [ENSECAT00000025523]	TTCTAGATGATCCTCCTCAAGAATAAAAGCACATCTGTGGATTGGACTTGGCTGCAGTGC
413	A_69_P029736	0,03	0,00	down	3,02	-3,02	-1,59	KCNMB2	potassium large conductance calcium-activated channel, subfamily M, beta member 2 [Source:HGNC Symbol;Acc:6286] [ENSECAT00000009305]	GGGGGGAAGAAGGAGGGATTGGTTTTAAACAAAAACATTCCATCTTTTATTTAACATCAA
414	A_69_P122692	0,05	0,00	down	1,70	-1,70	-0,77	KCNK12		TTTCCATCTCTCAGACCCTGGCGCTTTCTTAATCTCTATCCAATAAAATGAAACCAAAAA
415	A_69_P107353	0,05	0,00	up	1,15	1,15	0,20	HN1L	PREDICTED: Equus caballus hematological and neurological expressed 1-like (HN1L), mRNA [XM_001497464]	CTGTTCTCACCCTCTACGCTGCATTGAGAAATTACTAGTTTCTATTTTTCTTAAGCCTTA
416	A_69_P122511	0,03	0,00	up	1,29	1,29	0,37	TMEM243	transmembrane protein 243, mitochondrial [Source:HGNC Symbol;Acc:21707] [ENSECAT00000029037]	TTTTCTATCATCATGTTATGTATATGTGCAAACCTGTACTTCCATGATGTGGGAAAGTGA
417	A_69_P021621	0,04	0,00	up	1,17	1,17	0,23	NHP2L1	NHP2 non-histone chromosome protein 2-like 1 (S. cerevisiae) [Source:HGNC Symbol;Acc:7819] [ENSECAT00000000315]	GTCTCGTTTTGTCTGTTTTAGCATGTAGTATTTCCAGCTACCTTCTCCTGATATAAAATA
418	A_69_P034651	0,04	0,00	down	1,44	-1,44	-0,53	LYRM9	Uncharacterized protein [Source:UniProtKB/TrEMBL;Acc:F6Z674] [ENSECAT00000010046]	ACAAATATAAAAAACAAAACTGAGACTCCAGGGCCTGAGAGCTCTCCTCTCCTGGAGTTC
419	A_69_P062470	0,04	0,00	down	1,20	-1,20	-0,27	FAM53C	family with sequence similarity 53, member C [Source:HGNC Symbol;Acc:1336] [ENSECAT00000001697]	GTCTGTGTGTGTTAAACATGAGGTCCTGCCTCTGTGGCTGTGTTTGAAAAAATAAAGTTT

420	A_69_P117226	0,04	0,00	up	1,18	1,18	0,23	STK39	PREDICTED: Equus caballus serine threonine kinase 39 (STK39), mRNA [XM_001494117]	CGGGATGGTACCATTTGCTCTTTCCTAGCTAACCCTAGATATGGCAGCTCTTTAATGTAC
421	A_69_P025372	0,03	0,00	up	1,13	1,13	0,18	FITM2	fat storage-inducing transmembrane protein 2 [Source:HGNC Symbol;Acc:16135] [ENSECAT00000025439]	TCCCCAGAGCTGTAGTTTGAATTTGAAGCGAGACAGTTACAAGGAATAAAAGAGAAACAA
422	A_69_P105383	0,04	0,00	down	2,39	-2,39	-1,26	NCALD	PREDICTED: Equus caballus neurocalcin delta (NCALD), transcript variant X2, mRNA [XM_005613237]	CCATTGTCATTGCTGTGTTGAGGGACTTCTGGAAATGGAGTCCGTTCTGTCTGAAATCAA
423	A_69_P111262	0,04	0,00	down	1,44	-1,44	-0,52	JADE1	CT02037B2G01 Equine Articular Cartilage cDNA Library Equus caballus cDNA clone CT02037B2G01, mRNA sequence [CX604288]	CACTAGTGGCATATGTCGGGGAATCTGGGCTTCCAACATGAACGGATTCCTTAAGAAAAA
424	A_69_P105381	0,04	0,00	down	2,48	-2,48	-1,31	NCALD	PREDICTED: Equus caballus neurocalcin delta (NCALD), transcript variant X2, mRNA [XM_005613237]	ATTGTCATTGCTGTGTTGAGGGACTTCTGGAAATGGAGTCCGTTCTGTCTGAAATCAAGC
425	A_69_P062027	0,05	0,00	up	1,46	1,46	0,54	PRELID2	PRELI domain containing 2 [Source:HGNC Symbol;Acc:28306] [ENSECAT00000026846]	GACAGAGTTCATTCAAAGAGGCAGGATTTCAATCACAGGGGCTGGCTTTCTCAACTACAT
426	A_69_P081047	0,04	0,00	down	1,16	-1,16	-0,22	ITFG1	integrin alpha FG-GAP repeat containing 1 [Source:HGNC Symbol;Acc:30697] [ENSECAT00000014564]	AATAACTTGGGTTCTTTATTCACTTGTGCTGTGCATTTGTGTTCCTTTGTGGAATGTGTT
427	A_69_P057366	0,04	0,00	up	1,31	1,31	0,39	CEP70	centrosomal protein 70kDa [Source:HGNC Symbol;Acc:29972] [ENSECAT00000023685]	CCTTCTTGAAATCTTAGAAATTGATGACTTGGATGCCATTGTACCTGCAGTAAAGAAATT

428	A_69_P074081	0,05	0,00	up	1,83	1,83	0,87	HMCN1	hemicentin 1 [Source:HGNC Symbol;Acc:19194] [ENSECAT00000008543]	GGAGCAGTTACTTTCCAAAGATTATTCTGAACATCTAACAGGACATGTCAATGATGTTTT
429	A_69_P123551	0,04	0,00	down	1,69	-1,69	-0,75	TPM3	PREDICTED: Equus caballus tropomyosin 3 (TPM3), transcript variant X7, mRNA [XM_001496243]	CTGACTGTAGGATGTGTTCCATTGCGAGGATGCTTTCTAACAATAAAAGGACTGACCTGA
430	A_69_P104872	0,04	0,00	down	2,27	-2,27	-1,18	ANG	PREDICTED: Equus caballus angiogenin, ribonuclease, RNase A family, 5 (ANG), transcript variant X1, mRNA [XM_005602591]	CCCCTAGTAAGTCAGAGTATTAAAAATGTACTAGATCATTAAGAATCATTCACTCTTCCC
431	A_69_P108178	0,04	0,00	up	1,53	1,53	0,61		CT020017A10A01 Equine Articular Cartilage cDNA Library Equus caballus cDNA clone CT020017A10A01, mRNA sequence [CX597091]	AGGGGCACACAAAAAATACTGGTAATGTTTCATTTTTAAACTGTCGTGGTATATAGGTGC
432	A_69_P102586	0,03	0,00	up	1,80	1,80	0,85	ABCA1	PREDICTED: Equus caballus ATP-binding cassette, sub-family A (ABC1), member 1 (ABCA1), mRNA [XM_001493790]	AGATTCCTGCCTTTGCTTCTTTTGGACACTCTGGAAACCTTATTAACAACTGTGAATATG
433	A_69_P008406	0,05	0,00	down	1,66	-1,66	-0,73	DISP1	dispatched homolog 1 (Drosophila) [Source:HGNC Symbol;Acc:19711] [ENSECAT00000015234]	CCTGCTGTATTGACACACTCGGAACTTTCTGGTGAAAGTTTGTTAATAAAAACACTTTAA
434	A_69_P031057	0,04	0,00	up	2,06	2,06	1,04		ABI family, member 3 (NESH) binding protein [Source:HGNC Symbol;Acc:17265] [ENSECAT00000002615]	GAACCTGTCCAATTTGGAGAAATAGGTGGTCACACCCAAATCAATTATGTCCAGTGGTAT
435	A_69_P127891	0,04	0,00	up	1,11	1,11	0,15	RBM17	RNA binding motif protein 17 [Source:HGNC Symbol;Acc:16944] [ENSECAT00000009347]	TGATTTTAAGAACTAGAGCACAAGTCATCTCCAGTGACCCCTAAGTGAGCTGCAAGCTGA

436	A_69_P103633	0,05	0,00	down	1,56	-1,56	-0,64	SLC15A4	PREDICTED: Equus caballus solute carrier family 15 (oligopeptide transporter), member 4 (SLC15A4), mRNA [XM_005615190]	ACAGGTTTAGTGCAGTTAAAGGTAACTGCAAGTGTACAACGTGAACTTGGACTGTTGGAC
437	A_69_P000870	0,04	0,00	down	2,53	-2,53	-1,34	INOS	Equus caballus inducible nitric oxide synthase (INOS), mRNA [NM_001081769]	CAGGCACTTGGGTCTTCCTTGTATGTCTCCTTGATGGAGATATTTATATGAAATGCATTT
438	A_69_P074552	0,05	0,00	down	1,28	-1,28	-0,35	B4GALT3	UDP-Gal:betaGlcNAc beta 1,4-galactosyltransferase, polypeptide 3 [Source:HGNC Symbol;Acc:926] [ENSECAT00000023482]	CCCTCTTCTGGACCTGGCTCAGGGGGCTGGGATTTTGATATATTTTCTAATAAAGGACTT
439	A_69_P078172	0,04	0,00	up	1,10	1,10	0,14	MRPS24	mitochondrial ribosomal protein S24 [Source:HGNC Symbol;Acc:14510] [ENSECAT00000015296]	TAAATAGACTCCATGTGTCTTGCCTCTCAGTCTGTCTTTCCTCTTTAGGTGAGGGAACTA
440	A_69_P116254	0,04	0,00	down	2,15	-2,15	-1,10		LeukoS2_3_C09.b1_A024 Stimulated peripheral blood leukocytes S2 Equus caballus cDNA clone LeukoS2_3_C09_A024 3', mRNA sequence [CD469289]	GAAGAAGATTTTTAAAGTTTGAGAGTGGAAATAAATGGAAGCCCTGAGACCCTTCCAGAA
441	A_69_P065071	0,04	0,00	up	1,32	1,32	0,40	POP5	processing of precursor 5, ribonuclease P/MRP subunit (S. cerevisiae) [Source:HGNC Symbol;Acc:17689] [ENSECAT00000010367]	TTCCTGATTCAGTACAACCGGAGACAGCTGTTGATCTTGTTGCAACACTGCACTGATGAA
442	A_69_P063632	0,04	0,00	up	1,65	1,65	0,72	F2RL2	coagulation factor II (thrombin) receptor-like 2 [Source:HGNC Symbol;Acc:3539] [ENSECAT00000010830]	TATCTCATAGCCTTGTGCCTCGGTAGCTTGAACAGTTGCCTAGATCCATTCCTTTATTTT

443	A_69_P057252	0,04	0,00	down	1,60	-1,60	-0,68	RAB6B	RAB6B, member RAS oncogene family [Source:HGNC Symbol;Acc:14902] [ENSECAT00000026634]	AGGGATCACGTCACTCTTAACAGCTGTTACTTAAACAACTATTTTTTGGTTTGGTTGTAA
444	A_69_P003440	0,04	0,00	up	2,78	2,78	1,48	LOC100630543	Metallothionein [Source:UniProtKB/TrEMBL;Acc:F7DM22] [ENSECAT00000015995]	GCTGACCCAATTGCTGCATCCCCTTTTCTATGAAATATGTGAATGACAATAAAAGTTATT
445	A_69_P091481	0,05	0,00	down	1,21	-1,21	-0,27	NHSL2	Select seq ref|XM_005614263.2| PREDICTED: Equus caballus NHS like 2 (NHSL2), transcript variant X1, mRNA	ATAAACATGCAAGTTTGCGACACTCGTTGTTTAACACAGAGACAGCCGTGAACCCCAAGT
446	A_69_P062214	0,05	0,00	down	1,65	-1,65	-0,72	PCDHB7	protocadherin beta 7 [Source:HGNC Symbol;Acc:8692] [ENSECAT00000001301]	TCACAGAACACAGGGAGGGAAGTGGAAGAAAATCCTTCATTTGGGAATGATTTGGGTTTC
447	A_69_P026866	0,04	0,00	up	1,39	1,39	0,48		SWI/SNF related, matrix associated, actin dependent regulator of chromatin, subfamily a, member 2 [Source:HGNC Symbol;Acc:11098] [ENSECAT00000026315]	TTGCACAATGTCTTTGGTTGCAAGTCATAAGCCTGAGGCAAATAAAATTCCAGTAATTTC
448	A_69_P076606	0,04	0,00	down	1,46	-1,46	-0,55	PROK1	prokineticin 1 [Source:HGNC Symbol;Acc:18454] [ENSECAT00000023120]	AGGAGACGACTGCCACCCCGGCAGCCACAAGATCCCCTTCTTCAGAAAACGCCAGCACCA
449	A_69_P037401	0,04	0,00	down	1,15	-1,15	-0,20	ZSCAN16	zinc finger and SCAN domain containing 16 [Source:HGNC Symbol;Acc:20813] [ENSECAT00000010197]	CGAGTAAGTTCAGCCCTTATTAGACATCAAAGAATTCATACAGCAAATAAGCTCTACTAA

450	A_69_P065107	0,05	0,00	down	1,25	-1,25	-0,32	LOC100053420	Cytochrome c oxidase subunit 6A, mitochondrial [Source:UniProtKB/TrEMBL;Acc:F6RVC7] [ENSECAT00000013033]	TGACCTTCCGTCCCTTTGCTTATGGCAGGAGATGGCTTAAATAAATAACTTAGATTAGTC
451	A_69_P116116	0,04	0,00	down	1,39	-1,39	-0,48	CFL2	PREDICTED: Equus asinus cofilin 2 (muscle) (CFL2), mRNA	GCGTAAAGCCAAAAACTGATAAAATTAATGGTTCACATGTTACTTGAGACTAACTTGGCA
452	A_69_P102741	0,05	0,00	up	1,69	1,69	0,76	CCNG1	Equus caballus cyclin G1 (CCNG1), mRNA	GTCCTTAGTCCTTAGTAAAAACTTCTGGAGTGTTAAATCGGCAAGTTTTAAGAGGGATGT
453	A_69_P056906	0,04	0,00	up	1,35	1,35	0,44	RBMS3	Uncharacterized protein [Source:UniProtKB/TrEMBL;Acc:F6RCD0] [ENSECAT00000018866]	ATGTGCTGTGCAAATGGTTCCTTCACGTGGCGTGACAGTTTATTTTTGCCATAATTTTTT
454	A_69_P002421	0,04	0,00	down	4,54	-4,54	-2,18	DQB	Equus caballus MHC class II DQ-beta chain (DQB), mRNA [NM_001142813]	TTCCAGATCCTCGTGATGCTGGAAATGACTCCCCAGCGAGGAGATGTCTACACCTGCCAC
455	A_69_P097251	0,04	0,00	up	1,11	1,11	0,15	TM2D3	TM2 domain containing 3 [Source:HGNC Symbol;Acc:24128] [ENSECAT00000024103]	CGTACTTTCCCGAAAATGCTGTACTGCAATTGGACTGGAGGTTATAAGTGGTCTACTGCT
456	A_69_P120477	0,04	0,00	up	1,80	1,80	0,84	MAPK14	PREDICTED: Equus caballus mitogen-activated protein kinase 14 (MAPK14), mRNA [XM_005604060]	ACACGAGAAACTCACGGTGGATGAATGGAAGCAGCACATCTACAAGGAGATTGTGAACTT
457	A_69_P122691	0,05	0,00	down	1,71	-1,71	-0,77	KCNK12		TTCCATCTCTCAGACCCTGGCGCTTTCTTAATCTCTATCCAATAAAATGAAACCAAAAAA
458	A_69_P078231	0,04	0,00	up	1,18	1,18	0,24	NPC1L1	NPC1-like 1 [Source:HGNC Symbol;Acc:7898] [ENSECAT00000025973]	TCTATGTCAACTATGGCTTTGAACTTCCTGCCAAGAGTGCGGGCGGCAGCGGCTTTCTGC

459	A_69_P102121	0,04	0,00	down	2,23	-2,23	-1,16	CYTIP	PREDICTED: Equus asinus cytohesin 1 interacting protein (CYTIP), transcript variant X2, mRNA	GCTCTTTGCCTATATTCTAGAGCAGATGATCCAGACAATGCTAATAAATAGTCCTTACTG
460	A_69_P049087	0,05	0,00	up	1,33	1,33	0,41	USP45	ubiquitin specific peptidase 45 [Source:HGNC Symbol;Acc:20080] [ENSECAT00000024702]	TTTGAAAGAACCTGATAGTGAGTCGGCAGGCCAGTGGGTCCATGTTAGTGATACTTATGT
461	A_69_P020683	0,04	0,00	down	1,73	-1,73	-0,79	TMCC3	Uncharacterized protein [Source:UniProtKB/TrEMBL;Acc:F7AJN5] [ENSECAT00000012141]	ATGAAGAGCCGCTTCCACATTGTCGGCACCTTCTTTGCTGTGACTCTTCTTGCAATATTT
462	A_69_P109556	0,05	0,00	down	1,42	-1,42	-0,51	PRKAB2	Select seq ref|XM_008509324.1| PREDICTED: Equus przewalskii protein kinase, AMP-activated, beta 2 non-catalytic subunit (PRKAB2), mRNA	ACTCTGAGGTGTCTGAAAACTATGTGATATAACACATACAGAAATGTGAGGTTTTTTCCG
463	A_69_P106587	0,05	0,00	up	1,29	1,29	0,36	COLEC12	PREDICTED: Equus caballus collectin subfamily member 12 (COLEC12), mRNA	GTTAGCTCACTGATAATGCATCCTTTCAAAATACTGGACAGGGGTGACGGAGAAAAACTA
464	A_69_P034654	0,05	0,00	down	1,56	-1,56	-0,65	LYRM9	Uncharacterized protein [Source:UniProtKB/TrEMBL;Acc:F6Z674] [ENSECAT00000010046]	TGAACAAATATAAAAAACAAAACTGAGACTCCAGGGCCTGAGAGCTCTCCTCTCCTGGAG
465	A_69_P056486	0,04	0,00	up	1,24	1,24	0,31	ABHD5	abhydrolase domain containing 5 [Source:HGNC Symbol;Acc:21396] [ENSECAT00000023610]	GATGATGTGGGAAAACATACTGACTGATACAATCCCTCAGCAGTAATTCAGTCGATGGTG

466	A_69_P111172	0,03	0,00	down	1,82	-1,82	-0,87	FAM110B	Select seq ref|XM_014857796.1| PREDICTED: Equus asinus family with sequence similarity 110 member B (FAM110B), transcript variant X5, mRNA	AGCTGATAGGAAACTTTTTATGGTGTAAATGCTGTAAGACTTTGTACATACTTCGGTTGT
467	A_69_P113228	0,05	0,00	down	2,10	-2,10	-1,07	SLCO2A1	PREDICTED: Equus przewalskii solute carrier organic anion transporter family, member 2A1 (SLCO2A1), mRNA	GCTCTGCCTTCAACTGAGCTTACGAATGGTGAATGCCAATCTTGAGATTCTGGAAGATTG
468	A_69_P114666	0,05	0,00	down	1,64	-1,64	-0,72	CPE	PREDICTED: Equus przewalskii solute carrier organic anion transporter family, member 2A1 (SLCO2A1), mRNA	CTGATTCTATGGCTCTTATGCATCAGTTGTGGACATGGACTTTAAATAAATATACACACA
469	A_69_P117071	0,05	0,00	up	2,01	2,01	1,01	HMCN1	PREDICTED: Equus caballus hemicentin 1 (HMCN1), mRNA [XM_001491510]	CTGTACATGGTGGTAACAGACTCTAGAAGCAATTGCCAAGATGTATTCTATTTTTATGAA
470	A_69_P043707	0,04	0,00	up	1,26	1,26	0,33	NSMAF	neutral sphingomyelinase (N-SMase) activation associated factor [Source:HGNC Symbol;Acc:8017] [ENSECAT00000002019]	ATTTGTGGTTTTAAACTTTCTACATCATGGTGACTTCATTAAAAGCCGTTCGTTGCCATC
471	A_69_P043927	0,04	0,00	down	1,30	-1,30	-0,38	OSGIN2	oxidative stress induced growth inhibitor family member 2 [Source:HGNC Symbol;Acc:1355] [ENSECAT00000014116]	TCCTTTGGTTGGAGACAATTTTGTTCGATTCTTAAAGGGAGGGGCATTGGGTATTACACG
472	A_69_P037826	0,05	0,00	up	1,34	1,34	0,42	RPP21	TRIM39-RPP21 readthrough [Source:HGNC Symbol;Acc:38845] [ENSECAT00000017045]	GCTCCAGTCACCAGTGACAGATTCACCCTATCTTCCAAATAAATAAAGTTTAATTTTGTT

473	A_69_P122694	0,04	0,00	down	1,70	-1,70	-0,77			GCTTTCCATCTCTCAGACCCTGGCG CTTTCTTAATCTCTATCCAATAAAAT GAAACCAAA
474	A_69_P105384	0,04	0,00	down	2,51	-2,51	-1,33	NCALD	PREDICTED: Equus caballus neurocalcin delta (NCALD), transcript variant X2, mRNA [XM_005613237]	TCCATTGTCATTGCTGTGTTGAGGG ACTTCTGGAAATGGAGTCCGTTCTG TCTGAAATCA
475	A_69_P095886	0,03	0,00	down	1,94	-1,94	-0,96	DUSP13	dual specificity phosphatase 13 [Source:HGNC Symbol;Acc:19681] [ENSECAT00000000110]	CCAGTCTAGAGATTCTTTATGCAAAA GAGAGTTCAGTCTGTCTCTATAATAA AAGGTTCA
476	A_69_P072781	0,04	0,00	down	1,42	-1,42	-0,51	ABCC8	ATP-binding cassette, sub-family C (CFTR/MRP), member 8 [Source:HGNC Symbol;Acc:59] [ENSECAT00000022793]	CCTCCCACCTGGGGTTTCTAACTGT AAATCACTTGTAAATAAAGAGATTGG CTATTTCCT
477	A_69_P010871	0,05	0,00	down	1,17	-1,17	-0,23	ESRRA	estrogen-related receptor alpha [Source:HGNC Symbol;Acc:3471] [ENSECAT00000016651]	CAGAGACTCTATTTTAATGTATATTT GCTGCAAAGAGAAACCGCTTTTGGT TTTGAACCT
478	A_69_P114727	0,05	0,00	up	1,46	1,46	0,54	POC1A	HL02021A1F07 Equine placenta cDNA Library Equus caballus cDNA clone HL02021A1F07, mRNA sequence [DN510872]	CTGTGGGACTTGGGTAAATAAATAA AGGGGATTCCACTCTGTGGGAACCA CATCCAAAAA
479	A_69_P078171	0,05	0,00	up	1,09	1,09	0,12		mitochondrial ribosomal protein S24 [Source:HGNC Symbol;Acc:14510] [ENSECAT00000015296]	AAATAGACTCCATGTGTCTTGCCTC TCAGTCTGTCTTTCCTCTTTAGGTGA GGGAACTAG
480	A_69_P064271	0,04	0,00	up	2,94	2,94	1,55	SERPIND 1	serpin peptidase inhibitor, clade D (heparin cofactor), member 1 [Source:HGNC Symbol;Acc:4838] [ENSECAT00000014670]	GGGGCACTATAATTCTGTTTCCATT CTAACAATGAGAATGGAGATGTTCT GAGAAGCTTT

481	A_69_P056271	0,04	0,00	down	1,33	-1,33	-0,42	NBEAL2	neurobeachin-like 2 [Source:HGNC Symbol;Acc:31928] [ENSECAT00000016644]	TTTGTTCTACTGGGCACTGCCCAGTGTGCCCTGCACATCCTCCACCTGAATAAACTGCTC
482	A_69_P114569	0,03	0,00	up	1,23	1,23	0,30	FAM134A	HL02019B2G10 Equine placenta cDNA Library Equus caballus cDNA clone HL02019B2G10, mRNA sequence [DN510556]	TGACAGTGTGATATGTTTAGTGAGACTTAGCATGTGTGAATAAAGTGTATGCAGGAGGAA
483	A_69_P027327	0,04	0,00	down	1,42	-1,42	-0,50	BAG1	BCL2-associated athanogene [Source:HGNC Symbol;Acc:937] [ENSECAT00000019100]	GCCACTTTTCCTTCTCCTTCGCCTGCTCTCAATGAACGAGTATTGTCTTATAATCTTAAG
484	A_69_P050551	0,05	0,00	down	1,17	-1,17	-0,22	CUL3	cullin 3 [Source:HGNC Symbol;Acc:2553] [ENSECAT00000012128]	CTCGGAAAAATGTGAATGTGCCGCGTTTTGTTTTCAATACTGTATGAAAACAGGAAAAAA
485	A_69_P005970	0,04	0,00	up	1,77	1,77	0,82		Equus caballus clone 4-9 immunoglobulin heavy chain variable region mRNA, partial cds. [DQ125453]	CCAATATGATTACTTTGCTGGTGCTTATGGCCTCATCCCTTATGCTATAAAGTACTGGGG
486	A_69_P101363	0,04	0,00	up	1,41	1,41	0,50	RPS12	LeukoN2_5_F01.g1_A024 Unstimulated peripheral blood leukocytes N2 Equus caballus cDNA clone LeukoN2_5_F01_A024 5', mRNA sequence [CD466713]	ACTCCCTTTATGAGTGAAGCTTAATAGTCTGACAAACCTGTAGGTTCTGGGGCTCATTTC
487	A_69_P099056	0,04	0,00	down	1,62	-1,62	-0,69	C1H15orf52	chromosome 15 open reading frame 52 [Source:HGNC Symbol;Acc:33488] [ENSECAT00000018101]	AAGTCCAGACTTCCTCTGAGCCACAAAGAGGAGCAGGGCCCCTGGAACCCAGAGAAGATA
488	A_69_P034156	0,04	0,00	up	1,33	1,33	0,42	TRIM37	tripartite motif containing 37 [Source:HGNC Symbol;Acc:7523] [ENSECAT00000009956]	TTTCCTGATGGCGAACAAATAGGCCCTGAGGATCTCAGCTTCAATACCGATGAAAACAGT

489	A_69_P005417	0,05	0,00	up	1,21	1,21	0,28	ARG2	arginase 2 [Source:HGNC Symbol;Acc:664] [ENSECAT00000017770]	GAAATACACAATACAGGGTTGCTGTCAGCACTGGATCTTGTTGAAGTCAATCCTTGGGTG
490	A_69_P050382	0,05	0,00	down	1,16	-1,16	-0,22	ABCB6	ATP-binding cassette, sub-family B (MDR/TAP), member 6 [Source:HGNC Symbol;Acc:47] [ENSECAT00000017470]	TAGTGAGAGAAAGGGACCTTTCAGAAAAGCACTTTTGGGGGAAATAAAAGTATGAACAGT
491	A_69_P037953	0,03	0,00	down	1,63	-1,63	-0,71	EQMCE1	Equus caballus MHC class I antigen 3.7 (EQMCE1), mRNA [NM_001278933]	TCTTGCCATGAGGGATCAATGGGTTAGTTAAAGGAGAAGATTCCAAAAATTTGAGAGAGG
492	A_69_P112362	0,04	0,00	up	1,12	1,12	0,16	ENSA	PREDICTED: Equus caballus endosulfine alpha (ENSA), transcript variant 1, mRNA [XM_001490179]	CCACAGAGCCCAGGGGACTTTTTCAGTATTTGAAATAAAAAAGAAAAGAAGACCCACAAA
493	A_69_P105385	0,04	0,00	down	2,50	-2,50	-1,32	NCALD	PREDICTED: Equus caballus neurocalcin delta (NCALD), transcript variant X2, mRNA [XM_005613237]	TGTCCATTGTCATTGCTGTGTTGAGGGACTTCTGGAAATGGAGTCCGTTCTGTCTGAAAT
494	A_69_P127481	0,04	0,00	up	2,04	2,04	1,03	LOC100630653	Metallothionein [Source:UniProtKB/TrEMBL;Acc:F7DUC3] [ENSECAT00000000277]	AAAGAGTGCAGATGCACCTCCTGCAAGAAGAGCTGCTGCTCCTGCTGCCCCAGGGGCTGT
495	A_69_P010502	0,05	0,00	up	1,37	1,37	0,46	FADS1	fatty acid desaturase 1 [Source:HGNC Symbol;Acc:3574] [ENSECAT00000025250]	TTTCCTTTTTCTAGTTTGGCAGATGCAGTAGGTTGGTGAGCAAAGAGGAAGTCAGGAGAG
496	A_69_P015507	0,04	0,00	up	1,27	1,27	0,35	BRF2	BRF2, RNA polymerase III transcription initiation factor 50 kDa subunit [Source:HGNC Symbol;Acc:17298] [ENSECAT00000017862]	GAAATAGAGCAGTATTTGCGTACCCCTCAGGAAGTTAGGGACTTTCAAAAAGCCCAAGCT

497	A_69_P055621	0,03	0,00	up	1,49	1,49	0,58	GNL3	guanine nucleotide binding protein-like 3 (nucleolar) [Source:HGNC Symbol;Acc:29931] [ENSECAT00000015819]	GGTGTGTGGTGTACCAGTTTGTTACAGTATGGCATATGCTGTATAAATTTTGTGAATATG
498	A_69_P107043	0,05	0,00	down	1,99	-1,99	-0,99	FBLN7	PREDICTED: Equus caballus fibulin 7 (FBLN7), mRNA [XM_001916548]	AATGGTTTTCTACAAAATGCTCCATTCTGAGGACATACGATACTCCTGATGGACATTTGG
499	A_69_P129026	0,05	0,00	down	1,38	-1,38	-0,46	STK17B	TSA: Equus caballus contig26573.EqcaPBMC mRNA sequence. [JL616416]	GCACCTGGTGACTATCAAAAAGCCTGTTATTATGTTAAGATAACTTTAACCTCATGACAA
500	A_69_P049371	0,05	0,00	up	1,20	1,20	0,27	CDK19	cyclin-dependent kinase 19 [Source:HGNC Symbol;Acc:19338] [ENSECAT00000010295]	ACATTAGAGTGCGTATCCTACCTTTGCTTTGTCCTTGCTCCAGAGGATGAGTCCTATTCA
501	A_69_P008407	0,04	0,00	down	1,77	-1,77	-0,82	DISP1	dispatched homolog 1 (Drosophila) [Source:HGNC Symbol;Acc:19711] [ENSECAT00000015234]	ATTGTGAGAGTCAAGTGCAATTCTGTGGACTGCCAAATGCCAAACCTTGAAGCCAATGTG
502	A_69_P066886	0,05	0,00	up	1,79	1,79	0,84	CCBE1	collagen and calcium binding EGF domains 1 [Source:HGNC Symbol;Acc:29426] [ENSECAT00000008399]	AAGGGGTATAAATTTGTTCTTGGACAGTGCATCCCAGAAGATTATGATGTGTGCGCCGAG
503	A_69_P114568	0,03	0,00	up	1,24	1,24	0,31	FAM134A	HL02019B2G10 Equine placenta cDNA Library Equus caballus cDNA clone HL02019B2G10, mRNA sequence [DN510556]	GACAGTGTGATATGTTTAGTGAGACTTAGCATGTGTGAATAAAGTGTATGCAGGAGGAAA
504	A_69_P095970	0,04	0,00	up	1,31	1,31	0,39	PLAC9	placenta-specific 9 [Source:HGNC Symbol;Acc:19255] [ENSECAT00000011851]	AGAACAGAGCCGTGCATTCTGGCCTTCCTTCCTCAGCTTTGTACACAATAAAAGCTACTT

505	A_69_P125366	0,05	0,00	down	1,53	-1,53	-0,61	ZNF805	PREDICTED: Equus asinus zinc finger protein 805 (LOC106847400), transcript variant X3, mRNA	ACATCTTACTCAGAATCTGATCATTCATACCAAAGAGAAACCCCTCAGTTTTCTTCTCTG
506	A_69_P082966	0,05	0,00	down	2,89	-2,89	-1,53	SPP1	secreted phosphoprotein 1 [Source:HGNC Symbol;Acc:11255] [ENSECAT00000018317]	GTTTGGCCTACATTCTTTCCATATAAGAAATGCAAACTATCGCTGCATTTTAATGTTTGC
507	A_69_P095301	0,04	0,00	down	1,42	-1,42	-0,50	PRKG1	Select seq ref|XM_014866734.1| PREDICTED: Equus asinus protein kinase, cGMP-dependent, type I (PRKG1), transcript variant X2, mRNA	AGGAAGACTTTGCCAAGATTCTCATGCTCAAGGAAGAGAGGATCAAAGAGCTGGAAAAGC
508	A_69_P021276	0,04	0,00	up	1,32	1,32	0,40	NOL12	nucleolar protein 12 [Source:HGNC Symbol;Acc:28585] [ENSECAT00000011392]	TCTCTCACGGCATCACTGCACGCACACAGTCGCAAAAAGGTCAAGAGGAAACACCCCCGA
509	A_69_P058706	0,04	0,00	up	1,45	1,45	0,54	VAMP8	vesicle-associated membrane protein 8 [Source:HGNC Symbol;Acc:12647] [ENSECAT00000009704]	ATCATCGTCCTCCTCATTGTGCTCTTTGCCACTGGTGTCATCCATTAAGTAACTTAGGGA
510	A_69_P056746	0,04	0,00	up	1,45	1,45	0,54	DLEC1	deleted in lung and esophageal cancer 1 [Source:HGNC Symbol;Acc:2899] [ENSECAT00000021014]	TTCTTCACTGCCAGGAGCAGTAAGCTGTACGAGTCCACGCTGGTGGTGGAAGGTGTGCTT
511	A_69_P000867	0,04	0,00	down	2,43	-2,43	-1,28	NOS2	Equus caballus inducible nitric oxide synthase (INOS), mRNA [NM_001081769]	GCACTTGGGTCTTCCTTGTATGTCTCCTTGATGGAGATATTTATATGAAATGCATTTTGT
512	A_69_P003439	0,04	0,00	up	2,71	2,71	1,44	LOC100630543	Metallothionein [Source:UniProtKB/TrEMBL;Acc:F7DM22] [ENSECAT00000015995]	CTGACCCAATTGCTGCATCCCCTTTTCTATGAAATATGTGAATGACAATAAAAGTTATTG

513	A_69_P053057	0,05	0,00	down	1,23	-1,23	-0,30		cytochrome c oxidase assembly homolog 14 (S. cerevisiae) [Source:HGNC Symbol;Acc:28216] [ENSECAT00000008006]	AATGCGCATGTTTTCTCTGGTACTGCCATTTTGGGGAGGCCATCAGATCATTACAGGAAT
514	A_69_P059891	0,04	0,00	down	1,49	-1,49	-0,57	CDC42EP3	CDC42 effector protein (Rho GTPase binding) 3 [Source:HGNC Symbol;Acc:16943] [ENSECAT00000024281]	GAGGTGCTGAACGTCATGGATAAAAATAAGTAACAAAATGCCCATAACTATCCTCAAAGG
515	A_69_P063956	0,03	0,00	down	1,10	-1,10	-0,13	SNAP47	synaptosomal-associated protein, 47kDa [Source:HGNC Symbol;Acc:30669] [ENSECAT00000022288]	GTGGACCGGACAACCTTAACCATCGACAAGCATAATCGACGAATGAAAAAACTGACCTAG
516	A_69_P055701	0,05	0,00	up	1,58	1,58	0,66	POC1A	POC1 centriolar protein A [Source:HGNC Symbol;Acc:24488] [ENSECAT00000022802]	ATGTTGTCGATTATGGAGAAATCATAAAAGTGCAGAGGCCCCCAACTATGCTAGCCAGCT
517	A_69_P082721	0,04	0,00	down	1,88	-1,88	-0,91	SLC9B1	Uncharacterized protein [Source:UniProtKB/TrEMBL;Acc:F6SVC3] [ENSECAT00000013520]	GCCCAAACTTCTTACACGCTGTGATGCAAGCAAAATAAAAATGGAATTGACAGAATTAAA
518	A_69_P077186	0,03	0,00	down	1,68	-1,68	-0,75	GBP6	guanylate binding protein family, member 6 [Source:HGNC Symbol;Acc:25395] [ENSECAT00000013954]	TCTGCTCCTGCCAAACTTCTTGGTAATCTTGTGAAAGGCGTAGGCTCACTATTTAAAAAG
519	A_69_P096061	0,05	0,00	down	1,69	-1,69	-0,76		chromosome 10 open reading frame 10 [Source:HGNC Symbol;Acc:23355] [ENSECAT00000002986]	AGGAAAAGCGTCCAAGCAGGAGAGAACTGCCAAGGATGACTGGTCCTTCTGCTGAGCCCA
520	A_69_P011486	0,03	0,00	up	1,30	1,30	0,38	CHKA	choline kinase alpha [Source:HGNC Symbol;Acc:1937] [ENSECAT00000012200]	CTGCCGCACGCAGATGTGTATGATACGAAAGACTGTATTAAATGGAGTAACATTTCTTTC

521	A_69_P045535	0,05	0,00	down	1,19	-1,19	-0,25	LOC100051249	cytochrome c oxidase subunit VIb polypeptide 1 (ubiquitous) [Source:HGNC Symbol;Acc:2280] [ENSECAT00000012731]	ATGGTGATCCCCACCCTAGGATCCTGAATCATGGCTTAATAATAAATACTCGTTGGAAAA
522	A_69_P122636	0,05	0,00	down	1,69	-1,69	-0,76			CCTGCTTCTGATTTTATTGTTGTGAGGGAAACATACAATTGTGGTTACCTTCAAATTTTG
523	A_69_P029857	0,04	0,00	down	1,36	-1,36	-0,45	KLHL24	kelch-like family member 24 [Source:HGNC Symbol;Acc:25947] [ENSECAT00000022513]	CAGAGTGGGAACTTGACATATCTCCGTTGTGCGATATGCACAAAAAAGTAAAAATAATAA
524	A_69_P120002	0,05	0,00	down	1,18	-1,18	-0,24	CHRM3	cytochrome c oxidase subunit VIb polypeptide 1 (ubiquitous) [Source:HGNC Symbol;Acc:2280] [ENSECAT00000012731]	TCCCCACCCTAGGATCCTGAATCATGGCTTAATAATAAATACTCGTTGGAAAAGTGTAAG
525	A_69_P006976	0,04	0,00	up	1,32	1,32	0,40	PPAP2C	phosphatidic acid phosphatase type 2C [Source:HGNC Symbol;Acc:9230] [ENSECAT00000008747]	TTGTCTTTCTACTCTGGACACTCCTCCTTTGGGATGTACTGCATGATGTTCTTGGCGGTG
526	A_69_P018286	0,05	0,00	up	1,31	1,31	0,39	KCTD7	Uncharacterized protein [Source:UniProtKB/TrEMBL;Acc:F6YVX8] [ENSECAT00000026326]	AAGTGGATGTGTCTTTCGGGCCCTGGGAGGCTGTGGCTGATGTTTATGACCTGCTGCACT
527	A_69_P112616	0,04	0,00	up	1,19	1,19	0,25	DNPH1	2'-deoxynucleoside 5'-phosphate N-hydrolase 1 [Source:HGNC Symbol;Acc:21218] [ENSECAT00000006624]	AGAGGGAGAGGTGGAGGCCATGCTGGATCGATACTTTGAGGCTGATCCTTCTGAGGAGGT
528	A_69_P109745	0,04	0,00	down	2,23	-2,23	-1,16		CT02029B1C04 Equine Articular Cartilage cDNA Library Equus caballus cDNA clone CT02029B1C04, mRNA sequence [CX601398]	TGAGACAACATGCTATACACGGTTGACATTTTGAGTGTGAACAAAGTCAACTATGGGCAA

529	A_69_P096142	0,05	0,00	down	1,62	-1,62	-0,69	CHRM3	cholinergic receptor, muscarinic 3 [Source:HGNC Symbol;Acc:1952] [ENSECAT00000010538]	TGGGGGTGATTTCTGGTGATGATGAAAATGTTTTTATCACCCAGATATGAAAGAAGCTGC
530	A_69_P105471	0,04	0,00	down	1,96	-1,96	-0,97	CPE	CT02033A1F06 Equine Articular Cartilage cDNA Library Equus caballus cDNA clone CT02033A1F06, mRNA sequence [CX602587]	TGCCATTGTATTATTCAGCCTCTTAACACTACTTCAAAGTTTAGGGTTTTCTCTTGGTTG
531	A_69_P021746	0,04	0,00	up	1,16	1,16	0,22	MCAT	malonyl CoA:ACP acyltransferase (mitochondrial) [Source:HGNC Symbol;Acc:29622] [ENSECAT00000020168]	AACGTCAATGGGAACAAATACATGCATCCGAAACACATCCAGAAATTGCTGGTCCAGCAG
532	A_69_P062881	0,04	0,00	down	1,38	-1,38	-0,46	GRAMD3	GRAM domain containing 3 [Source:HGNC Symbol;Acc:24911] [ENSECAT00000018808]	TTACAAAAGCTGCTTGAGAACGGTGACTGATACACCAGATTGCTTGGGCCAACACAATAC
533	A_69_P076031	0,04	0,00	up	1,48	1,48	0,56	MTMR11	myotubularin related protein 11 [Source:HGNC Symbol;Acc:24307] [ENSECAT00000025124]	GTGCTAACCTTCTCCAACTCTTGTCCTCATTTGTTTCTTGTGATTTGAAGAATTAAGAAA
534	A_69_P072821	0,05	0,00	down	1,88	-1,88	-0,91	SAA1	Equus caballus serum amyloid A1 (SAA), mRNA. [Source:RefSeq mRNA;Acc:NM_001081853] [ENSECAT00000013971]	ATGGCCCCAGAAGCCAGTGGGGGTCACAGAATTAGTGTCTAATAAATGCTTAAGAGATTG
535	A_69_P111155	0,04	0,00	up	1,62	1,62	0,69		CT02037A2H10 Equine Articular Cartilage cDNA Library Equus caballus cDNA clone CT02037A2H10, mRNA sequence [CX604126]	CACTTATATTTCATTTCAGCTTGTTTGAGGTGCCAGTAGGACAGCTCATATGTGTGGGAA
536	A_69_P117972	0,04	0,00	up	1,23	1,23	0,30	RPL29	PREDICTED: Equus caballus ribosomal protein L29 (RPL29), mRNA	TGGGCTGGTGTCCTCCTGTGCTATTTGTACAAATAAACCTGAGGCAGGATCTGTCAAAAA

537	A_69_P082026	0,04	0,00	down	1,45	-1,45	-0,53	MTSS1L	metastasis suppressor 1-like [Source:HGNC Symbol;Acc:25094] [ENSECAT00000000215]	TGATCAAAGACCTGAAGGGCTCTGACTACAGCTGGTCATACCAGACCCCACCCTCATCAC
538	A_69_P117821	0,05	0,00	down	1,32	-1,32	-0,41	MEF2A	Select seq ref|XM_005602835.2| PREDICTED: Equus caballus myocyte enhancer factor 2A (MEF2A), transcript variant X5, mRNA	CAAGCTGGACTTTGTTGCCATCCTTGAGATGAACCTTTTAAGAAAAATAAGTTAATCTCA
539	A_69_P065060	0,04	0,00	up	1,43	1,43	0,52	UNC119B	Select seq ref|XM_005612553.2| PREDICTED: Equus caballus unc-119 lipid binding chaperone B (UNC119B), mRNA	ATGAGTTTCCCCAGCTTTCTGAGGATGTCATTCGTTTGATGATTGAAAATCCCTATGAGA
540	A_69_P003438	0,04	0,00	up	2,76	2,76	1,47	LOC100630543	Metallothionein [Source:UniProtKB/TrEMBL;Acc:F7DM22] [ENSECAT00000015995]	GACCCAATTGCTGCATCCCCTTTTCTATGAAATATGTGAATGACAATAAAAGTTATTGAC
541	A_69_P098836	0,03	0,00	down	1,61	-1,61	-0,68	ZNF106	zinc finger protein 106 [Source:HGNC Symbol;Acc:12886] [ENSECAT00000025152]	AAACAGGATGCTGCAGGACACATTGAACGACATGCTGAGGATGACAGCAAAATTGATTCA
542	A_69_P094540	0,03	0,00	up	1,11	1,11	0,16	SFXN2	sideroflexin 2 [Source:HGNC Symbol;Acc:16086] [ENSECAT00000026036]	AAGACACCATCAGGGCCAAGTATAGAGAACTCGTGCCTTATGTCTACTTCAATAAGGGTC
543	A_69_P044272	0,04	0,00	down	1,27	-1,27	-0,35	UBR5	ubiquitin protein ligase E3 component n-recognin 5 [Source:HGNC Symbol;Acc:16806] [ENSECAT00000017297]	CTGAGGGGAAAAAATGTAAAATGTTCTGAAAATTCACTGCTGCCTTTGTGGAAAGTGTTT

544	A_69_P113857	0,03	0,00	down	1,27	-1,27	-0,34	GRB10	HL02015B1D08 Equine placenta cDNA Library Equus caballus cDNA clone HL02015B1D08, mRNA sequence [DN509322]	GCTCGTGAACCTAATTGTAAACTTTCAGGTATTTTTGTACAAATAAGGGACTGATGTTCT
545	A_69_P051621	0,05	0,00	up	1,15	1,15	0,20	GPR162	G protein-coupled receptor 162 [Source:HGNC Symbol;Acc:16693] [ENSECAT00000020397]	TGCCCTACTCACCTTCCATCATCCCTAGCAAATGTATTAAAGTCTGAAGTGTTACTATGG
546	A_69_P004039	0,05	0,00	up	1,22	1,22	0,29	HNRNPD	Select seq ref|XM_003364678.3| PREDICTED: Equus caballus heterogeneous nuclear ribonucleoprotein D like (HNRNPDL), transcript variant X2, mRNA	ATTACCAGCCATACTAAAGGAGGACATTGGAGAAAACAGGAGGAGATTGCTAAAGTAACC
547	A_69_P116446	0,04	0,00	down	1,21	-1,21	-0,28			CCCTTGAATTTGAACACACCTTCTGCCCACATTTCTTTGTAAAGTTTTTCTCAAATAAAC
548	A_69_P013196	0,05	0,00	down	1,69	-1,69	-0,76	MURC	muscle-related coiled-coil protein [Source:HGNC Symbol;Acc:33742] [ENSECAT00000006670]	CCCCAAGTGAAAGTAGAGGATGATGAATCTCTTTTGCTAGATTTAAAGCAGTCATCATAA
549	A_69_P034668	0,04	0,00	up	1,20	1,20	0,26	TMEM97	transmembrane protein 97 [Source:HGNC Symbol;Acc:28106] [ENSECAT00000012907]	TTCTGCTTTTCATGTTGTGGAGCCCCCACTACAAGTATGAGGAGAAAAGAAAGAAAAAAT
550	A_69_P013555	0,04	0,00	up	1,25	1,25	0,32	SLC46A2	solute carrier family 46, member 2 [Source:HGNC Symbol;Acc:16055] [ENSECAT00000019674]	AATTGGCATCGTGGCCTATAAACAAGCCTCATGGTTGCAATATGGAGACATGACAGAGAA

551	A_69_P113859	0,03	0,00	down	1,26	-1,26	-0,33	GRB10	HL02015B1D08 Equine placenta cDNA Library Equus caballus cDNA clone HL02015B1D08, mRNA sequence [DN509322]	TGGCTCGTGAACCTAATTGTAAACTTTCAGGTATTTTTGTACAAATAAGGGACTGATGTT
552	A_69_P106230	0,04	0,00	up	1,28	1,28	0,35	SCARA3	PREDICTED: Equus caballus scavenger receptor class A, member 3 (SCARA3), mRNA	TCCTGTCTACATATGATATCATGCGACATAGTATTACTGACTCAGTAAAGAGCTATTTCT
553	A_69_P090517	0,03	0,00	down	2,07	-2,07	-1,05	DMD	Uncharacterized protein [Source:UniProtKB/TrEMBL;Acc:F6Z1L7] [ENSECAT00000023688]	AGCCATGGAGTCCTTAGTTTCAGTCATGACAGATGAAGAAGGGGCAGAATAAATGTTTTA
554	A_69_P065106	0,05	0,00	down	1,28	-1,28	-0,35	LOC100053420	Cytochrome c oxidase subunit 6A, mitochondrial [Source:UniProtKB/TrEMBL;Acc:F6RVC7] [ENSECAT00000013033]	GACCTTCCGTCCCTTTGCTTATGGCAGGAGATGGCTTAAATAAATAACTTAGATTAGTCA
555	A_69_P111703	0,05	0,00	down	1,16	-1,16	-0,22	RBFOX2	CT02040B2A02 Equine Articular Cartilage cDNA Library Equus caballus cDNA clone CT02040B2A02, mRNA sequence [CX605190]	TTATTGATCATGTTGCTGGCTTTTATAAACTCTAAGCGAAGGAGGAGCTCTGCCTCTGCC
556	A_69_P103912	0,04	0,00	down	1,07	-1,07	-0,10	GNB1	PREDICTED: Equus caballus guanine nucleotide binding protein (G protein), beta polypeptide 1 (GNB1), mRNA	TTGTATTTAATTTTAAGGTCGGTGTACTGCAAGGAAGCTGGATGCAAGATAGATACTATA
557	A_69_P095888	0,03	0,00	down	1,88	-1,88	-0,91		dual specificity phosphatase 13 [Source:HGNC Symbol;Acc:19681] [ENSECAT00000000110]	ACCCAGTCTAGAGATTCTTTATGCAAAAGAGAGTTCAGTCTGTCTCTATAATAAAAGGTT
558	A_69_P086861	0,04	0,00	down	1,43	-1,43	-0,52		chromosome 1 open reading frame 63 [Source:HGNC Symbol;Acc:25234] [ENSECAT00000007766]	AAATCAGCTAAAGCTACTGCTGAAGAGACCCTCTCAGGATCCCCAAAAATAAATCTGAAA

559	A_69_P060666	0,05	0,00	down	1,42	-1,42	-0,50	KLF11	Kruppel-like factor 11 [Source:HGNC Symbol;Acc:11811] [ENSECAT00000016631]	AACTATGTTTGCAACTTCACAGGCTGCCGGAAAACCTACTTCAAAAGTTCCCACCTCAAG
560	A_69_P005419	0,03	0,00	up	1,21	1,21	0,28	ARG2	arginase 2 [Source:HGNC Symbol;Acc:664] [ENSECAT00000017770]	AGGAAATACACAATACAGGGTTGCTGTCAGCACTGGATCTTGTTGAAGTCAATCCTTGGG
561	A_69_P066186	0,04	0,00	up	1,53	1,53	0,61	COLEC12	collectin sub-family member 12 [Source:HGNC Symbol;Acc:16016] [ENSECAT00000020273]	GAGTTCTCAAAGGCAAAGGATAATTCTTTTTGATTGCATCACCTTCTCCCCAGATTGAAA
562	A_69_P106901	0,03	0,00	down	1,75	-1,75	-0,80	TGFB2	CT020022A10A01 Equine Articular Cartilage cDNA Library Equus caballus cDNA clone CT020022A10A01, mRNA sequence [CX598801]	GGAAGCTTCTTGTAAGGTTCAAAACTGAAAAGCCTGTTAATAAAGAAAACTTTCAGTCAG
563	A_69_P071341	0,04	0,00	up	1,26	1,26	0,33	ANAPC15	anaphase promoting complex subunit 15 [Source:HGNC Symbol;Acc:24531] [ENSECAT00000022428]	AGATGAATGACTACAATGAGTCACCTGATGATGGAGAGGTCAATGAGGTGGACATGGAAG
564	A_69_P021622	0,05	0,00	up	1,17	1,17	0,23	NHP2L1	NHP2 non-histone chromosome protein 2-like 1 (S. cerevisiae) [Source:HGNC Symbol;Acc:7819] [ENSECAT00000000315]	TGTCTCGTTTTGTCTGTTTTAGCATGTAGTATTTCCAGCTACCTTCTCCTGATATAAAAT
565	A_69_P115861	0,04	0,00	down	1,56	-1,56	-0,64	MEF2C	PREDICTED: Vicugna pacos myocyte enhancer factor 2C (MEF2C), transcript variant X8, mRNA	CTCCTGCTGGAAATAAGGTATAATTTGTATTTTGCAGACAATTCAGTAAAGTTACTGGCT
566	A_69_P058571	0,04	0,00	up	1,23	1,23	0,30	RPIA	ribose 5-phosphate isomerase A [Source:HGNC Symbol;Acc:10297] [ENSECAT00000012343]	TATTCATCAACATGGCTGAGAGAGTTTACTTTGGCATGCAGGACGGCTCAGTGAACATGA

567	A_69_P116591	0,03	0,00	up	2,09	2,09	1,06			TGCAGATGCGCCTCCTGCAAGAAGAGTGAGTGGAATCCTCTTTTCATCCACCCCTCAGCC
568	A_69_P018306	0,04	0,00	up	1,15	1,15	0,20	VKORC1L1	vitamin K epoxide reductase complex, subunit 1-like 1 [Source:HGNC Symbol;Acc:21492] [ENSECAT00000011515]	TTCATTCTTCTTATCATCAACTACAAACGACTAGTTTACTTAAATGAGGCCTGGAAACGG
569	A_69_P002096	0,05	0,00	down	1,42	-1,42	-0,50	SLC8A3	Uncharacterized protein [Source:UniProtKB/TrEMBL;Acc:F6VU44] [ENSECAT00000024282]	GAAACACCCACTTTACAAAGTATTTAATTCAATACAAGCCAACAACAGCAACAAACCCAC
570	A_69_P117046	0,05	0,00	down	1,09	-1,09	-0,13	SLMAP	PREDICTED: Equus caballus sarcolemma associated protein (SLMAP), transcript variant X20, mRNA	CAATAGAATTTCTCGAGTACAGATTAAACTATTTGCACTAACACACGTGATGTGCATGAT
571	A_69_P097932	0,05	0,00	up	1,29	1,29	0,37	LRRC49	leucine rich repeat containing 49 [Source:HGNC Symbol;Acc:25965] [ENSECAT00000026212]	GCTTTGCAGAAGCTTTGGCCACAAATGTTCATTGAGCTTGTTAGGGATGCAGTCATAGAA
572	A_69_P017489	0,04	0,00	up	1,24	1,24	0,31	NUDT1	nudix (nucleoside diphosphate linked moiety X)-type motif 1 [Source:HGNC Symbol;Acc:8048] [ENSECAT00000009517]	GACAGATACTGGTTTCCCCTCTTGCTTCAGAAGAAGAAATTCCATGGGTACTTCAAGTTC
573	A_69_P020056	0,05	0,00	up	1,09	1,09	0,12	GNPTG	N-acetylglucosamine-1-phosphate transferase, gamma subunit [Source:HGNC Symbol;Acc:23026] [ENSECAT00000011965]	CTCACAGGTTGTGCTCAAAGATGTAGACAAAGTAAAGAGTCAAAGTTTTAATTAATTCCC

574	A_69_P012302	0,03	0,00	down	1,56	-1,56	-0,64	PTPLA	protein tyrosine phosphatase-like (proline instead of catalytic arginine), member A [Source:HGNC Symbol;Acc:9639] [ENSECAT00000021501]	AGGTGATTGTAGAAAAGGATGATTAAATGATCCCTACAAACACGGTGCTTTTTCCAGAAC
575	A_69_P071345	0,04	0,00	up	1,22	1,22	0,28	ANAPC15	anaphase promoting complex subunit 15 [Source:HGNC Symbol;Acc:24531] [ENSECAT00000022428]	GATGAGATGAATGACTACAATGAGTCACCTGATGATGGAGAGGTCAATGAGGTGGACATG
576	A_69_P076263	0,04	0,00	up	1,29	1,29	0,37	TBX15	Uncharacterized protein [Source:UniProtKB/TrEMBL;Acc:F6VPR5] [ENSECAT00000025103]	GCCCAACACCTTTGAAAAACAGGAACTGTGTATTATTTTTATTTTCTTTTCTGGAGGAGG
577	A_69_P118094	0,05	0,00	down	1,17	-1,17	-0,22	FAM156A	PREDICTED: Equus caballus family with sequence similarity 156, member A (FAM156A), transcript variant X2, mRNA [XM_005614210]	ATGCGGCAGGAAAGCAAAAGAATGTTTCAGAGGCTCCTAAAACAGTGGTTAAAAGAAAAC
578	A_69_P020676	0,04	0,00	up	1,33	1,33	0,41	CCDC41	coiled-coil domain containing 41 [Source:HGNC Symbol;Acc:17966] [ENSECAT00000008537]	CTGAACGCTTGGAAAAAGAGCTACAATCAAGCAGTGAACAAAATACTGTTTTAATCAGTA
579	A_69_P116902	0,04	0,00	up	1,97	1,97	0,98	ABI3BP	PREDICTED: Equus caballus ABI family member 3 binding protein (ABI3BP), transcript variant X26, mRNA	TAGGACCTCCAGGAATGAATGTATCAGAGGATGTTTTCAAATTCTAACTCAACCTATAAT
580	A_69_P098757	0,05	0,00	down	1,20	-1,20	-0,27	TP53BP1	tumor protein p53 binding protein 1 [Source:HGNC Symbol;Acc:11999] [ENSECAT00000008867]	GAGATTGTGTTTTAACCAGGTTTTAAATGTGTCTTGTGTGTAACTGGATTCCTTGCATGG
581	A_69_P052431	0,04	0,00	up	1,39	1,39	0,48	REP15	RAB15 effector protein [Source:HGNC Symbol;Acc:33748] [ENSECAT00000011159]	TGTCTGAGTAAGAAAGATGAGCTGCGAGAGGTGGGCAAGGTTTATATTAGCATCCTCTGA

582	A_69_P031056	0,04	0,00	up	1,99	1,99	0,99	ABI3BP	ABI family, member 3 (NESH) binding protein [Source:HGNC Symbol;Acc:17265] [ENSECAT00000002615]	CACACCCAAATCAATTATGTCCAGTGGTATGAATGCGGGACCACAATTCCTGGAAAATGG
583	A_69_P017486	0,03	0,00	up	1,24	1,24	0,31	NUDT1	nudix (nucleoside diphosphate linked moiety X)-type motif 1 [Source:HGNC Symbol;Acc:8048] [ENSECAT00000009517]	AGATACTGGTTTCCCCTCTTGCTTCAGAAGAAGAAATTCCATGGGTACTTCAAGTTCCGG
584	A_69_P004147	0,04	0,00	up	1,26	1,26	0,34	SOD2	Equus caballus superoxide dismutase 2, mitochondrial (SOD2), mRNA [NM_001082517]	TACATGGCTTGCAAAAAGTAAAGCATTATCGTTACACCGAGTGCATTAAGCAATTTCCAA
585	A_69_P095887	0,04	0,00	down	1,84	-1,84	-0,88		dual specificity phosphatase 13 [Source:HGNC Symbol;Acc:19681] [ENSECAT00000000110]	CCCAGTCTAGAGATTCTTTATGCAAAAGAGAGTTCAGTCTGTCTCTATAATAAAAGGTTC
586	A_69_P041492	0,04	0,00	up	1,72	1,72	0,78	STEAP3	STEAP family member 3, metalloreductase [Source:HGNC Symbol;Acc:24592] [ENSECAT00000006653]	ACACACACGTGCGTGTGATGTATATGTTCCTATATATTTCATATGTATAACAGGATTTGC
587	A_69_P038317	0,05	0,00	down	1,18	-1,18	-0,23	DRB	Equus caballus MHC class II DR-beta chain (DRB), mRNA [NM_001142811]	TGCACCTGAACTCACCTCTGCCCACATTTCTTTATAAAGTTTTTCTCAAATAAACATGGA
588	A_69_P059791	0,04	0,00	up	1,29	1,29	0,36	EML4	echinoderm microtubule associated protein like 4 [Source:HGNC Symbol;Acc:1316] [ENSECAT00000008379]	TCAGAAATTACTGTGTGTGTAAGTGGTATGGTGTAAATACTGGAACAAAAGCGGCAGTTG
589	A_69_P080471	0,03	0,00	up	1,90	1,90	0,92	FAM115C	family with sequence similarity 115, member C [Source:HGNC Symbol;Acc:26878] [ENSECAT00000014923]	ATCTATGGGTCAAGAAGTTCTCTGAAAAGGTGCAGAAGAATCTGGCTCCGTTCTTTGAGG

590	A_69_P120476	0,05	0,00	up	1,83	1,83	0,87	MAPK14	PREDICTED: Equus caballus mitogen-activated protein kinase 14 (MAPK14), mRNA [XM_005604060]	ACGAGAAACTCACGGTGGATGAATGGAAGCAGCACATCTACAAGGAGATTGTGAACTTCA
591	A_69_P108991	0,03	0,00	down	1,86	-1,86	-0,89	FAM107A	PREDICTED: Equus caballus family with sequence similarity 107 member A (FAM107A), transcript variant X3, mRNA	GAGATGTAAATAATTGCCAATCTGCATTTCTTACCACACTGTTATGATTCAATCTTAGCC
592	A_69_P094496	0,04	0,00	up	1,37	1,37	0,45	TAF5	TAF5 RNA polymerase II, TATA box binding protein (TBP)-associated factor, 100kDa [Source:HGNC Symbol;Acc:11539] [ENSECAT00000006298]	GATCCACTAACATTTTTGCTTGGCCCATGATTAGTGGAATGTACGTTAACTGGGTAGGGT
593	A_69_P065072	0,03	0,00	up	1,35	1,35	0,43	POP5	processing of precursor 5, ribonuclease P/MRP subunit (S. cerevisiae) [Source:HGNC Symbol;Acc:17689] [ENSECAT00000010367]	AAGTTCCTGATTCAGTACAACCGGAGACAGCTGTTGATCTTGTTGCAACACTGCACTGAT
594	A_69_P102596	0,05	0,00	up	1,27	1,27	0,34	F2R	TSA: Equus caballus contig28377.EqcaPBMC mRNA sequence. [JL641856]	CAACTGAGTGGAGATTTAGTCATTGTTCATTCAGTCTGTATGTATTAAAATACCTCTGAC
595	A_69_P111931	0,05	0,00	down	1,50	-1,50	-0,58	SPON1	Select seq ref|XM_014844124.1| PREDICTED: Equus asinus spondin 1, extracellular matrix protein (SPON1), mRNA	CTCTTTCTTACCAAAGTCTTGTGTTAGGTGGTTTATAGTTTTTCTGGCTAACCAATCATT
596	A_69_P056123	0,03	0,00	down	1,14	-1,14	-0,19	IP6K2	inositol hexakisphosphate kinase 2 [Source:HGNC Symbol;Acc:17313] [ENSECAT00000007129]	GTCCCAGCCTGAGCCAGCCCTAACTGTGCTCGGAGTCTATTTATTTTAACTATTTCTTCA
597	A_69_P090518	0,04	0,00	down	2,03	-2,03	-1,02		Uncharacterized protein [Source:UniProtKB/TrEMBL;Acc:F6Z1L7] [ENSECAT00000023688]	GAGCCATGGAGTCCTTAGTTTCAGTCATGACAGATGAAGAAGGGGCAGAATAAATGTTTT

598	A_69_P113227	0,05	0,00	down	2,16	-2,16	-1,11	SLCO2A1	Select seq ref|XM_008512913.1| PREDICTED: Equus przewalskii solute carrier organic anion transporter family, member 2A1 (SLCO2A1), mRNA	TACGAATGGTGAATGCCAATCTTGAGATTCTGGAAGATTGATTGCCCTCCGAAGCCTTTA
599	A_69_P058616	0,05	0,00	down	1,37	-1,37	-0,45	RMND5A	required for meiotic nuclear division 5 homolog A (S. cerevisiae) [Source:HGNC Symbol;Acc:25850] [ENSECAT00000021214]	AGGAAGAAAGCTGTGGTAGATTCCAAATTTGCTTTTTGATGTTTTCCTCTGCTCCAGCTC
600	A_69_P105857	0,05	0,00	up	1,17	1,17	0,22	GNA12	PREDICTED: Equus caballus guanine nucleotide binding protein (G protein) alpha 12 (GNA12), transcript variant X3, mRNA [XM_005598542]	CCCTCTGTGTATTACTCTACTAAAAAAGTCTGTCTATTGTGTTTAGGACTTTTCCTAGCA
601	A_69_P066187	0,04	0,00	up	1,65	1,65	0,73	COLEC12	collectin sub-family member 12 [Source:HGNC Symbol;Acc:16016] [ENSECAT00000020273]	TAAAGGACGGTGATGTAATAACATGAGCAAATTTTCAAAGAGTTCTCAAAGGCAAAGGAT
602	A_69_P079277	0,04	0,00	up	1,49	1,49	0,57	GGCT	gamma-glutamylcyclotransferase [Source:HGNC Symbol;Acc:21705] [ENSECAT00000018919]	CTGGAGTATCAAAAGATGTTAAATGCAATAGAACCAAATGACTACAAAGGAAAGGTCTCA
603	A_69_P032622	0,04	0,00	up	1,41	1,41	0,50	ARHGAP27	Rho GTPase activating protein 27 [Source:HGNC Symbol;Acc:31813] [ENSECAT00000006684]	TTTTTTCCCCTGGCCTGTTCTGCTAGAGGTTAATTGGGTTCTGTTCTTTATTACAGCGCC
604	A_69_P033696	0,04	0,00	down	1,26	-1,26	-0,34	HOXB9	homeobox B9 [Source:HGNC Symbol;Acc:5120] [ENSECAT00000002621]	AAATACCAGACGCTGGAGCTAGAGAAAGAGTTTCTGTTCAATATGTACCTCACCAGGGAC

605	A_69_P084436	0,05	0,00	up	1,41	1,41	0,50	SLC2A9	solute carrier family 2 (facilitated glucose transporter), member 9 [Source:HGNC Symbol;Acc:13446] [ENSECAT00000005389]	AACAAGGCATTCCCACCAGAGGAGAAGATCGACTTAGCTGTAACTGACGACAAGATGATT
606	A_69_P025436	0,05	0,00	down	1,21	-1,21	-0,28	KCNS1	potassium voltage-gated channel, delayed-rectifier, subfamily S, member 1 [Source:HGNC Symbol;Acc:6300] [ENSECAT00000010717]	CCAAAGTCTCAGATTTATTAAAACCAAGGTTCCATGGGCCTCTTCCCACATCCCTACAGT
607	A_69_P064743	0,04	0,00	down	1,76	-1,76	-0,81	C8H22orf31	chromosome 22 open reading frame 31 [Source:HGNC Symbol;Acc:26931] [ENSECAT00000011961]	GCTCATGGGATCAGGATATTCTCTGCTAGATGTCTGAAAAATAAACACCACTTTGCACCT
608	A_69_P038104	0,05	0,00	up	1,29	1,29	0,36	LY6G5B	lymphocyte antigen 6 complex, locus G5B [Source:HGNC Symbol;Acc:13931] [ENSECAT00000008570]	TGTTGCTGTCCTGCGCCACATATACTTGTTCCTTAACAATTCAGGACTTTTGGTTCTTCC
609	A_69_P025772	0,05	0,00	up	1,13	1,13	0,17	UBE2V1	ubiquitin-conjugating enzyme E2 variant 1 [Source:HGNC Symbol;Acc:12494] [ENSECAT00000012403]	CTAGATACGTCTTGTAGACCTCAAAGTACTGGAAAGGAAGCTCCCATTCAAAGGCAATTT
610	A_69_P119341	0,03	0,00	down	1,60	-1,60	-0,68	LOC102147361	PREDICTED: Equus caballus uncharacterized LOC102147361 (LOC102147361), misc_RNA [XR_288371]	CGAAAAACAAGCACTAAAGGGTCCCCAACACTTGCAGCCCTGGTTCAAGAAACATCATAA
611	A_69_P033946	0,05	0,00	down	1,29	-1,29	-0,37	CA10	carbonic anhydrase X [Source:HGNC Symbol;Acc:1369] [ENSECAT00000013652]	ACACATGCAAACATATCTATATACATGTAAACTCACCCAGCCTCCTTCATGGGAAGTCAA

612	A_69_P111106	0,05	0,00	up	1,23	1,23	0,29	PEX3	PREDICTED: Equus przewalskii peroxisomal biogenesis factor 3 (PEX3), mRNA	GCTGAAATTGTGTTGATGCGGTTTACTCACGGCTGTTATTAACTACATAGTTTCAATAAT
613	A_69_P004086	0,03	0,00	up	1,13	1,13	0,18	RPS26	Equus caballus ribosomal protein S26 (RPS26), mRNA [NM_001163890]	TTACATTACTGTGTGAGTTGTGCCATTCACAGCAAGGTAGTCAGGAATCGCTCTCGTGAA
614	A_69_P006328	0,05	0,00	up	1,29	1,29	0,36	TLR1	Equus caballus toll-like receptor 1 (TLR1), mRNA [NM_001256899]	TATGGGGAAAAAGAAGACTCTGAGAGCCTTCGACACCTTAACACAGAGAGTCTGCACATT
615	A_69_P019208	0,05	0,00	down	1,67	-1,67	-0,74	SYT17	synaptotagmin XVII [Source:HGNC Symbol;Acc:24119] [ENSECAT00000009185]	ATCCTTTCTACAACGAGTCCTTCAGCTTCAAAGTTCCCCAAGAAGAACTGGAAAATGCCA
616	A_69_P117882	0,03	0,00	down	5,97	-5,97	-2,58	TBC1D8	PREDICTED: Equus caballus TBC1 domain family member 8 (TBC1D8), mRNA	CCCATTGAAGCCATTTACCTGACCCTAGAGCTTTTATCATAATTAAACATGATTATGGGT
617	A_69_P113226	0,05	0,00	down	2,05	-2,05	-1,03	SLCO2A1	HL0200PREDICTED: Equus asinus solute carrier organic anion transporter family member 2A1 (SLCO2A1), mRNA	CTTGAGATTCTGGAAGATTGATTGCCCTCCGAAGCCTTTATTCTATTATGATATTGCTAT
618	A_69_P031841	0,03	0,00	down	1,24	-1,24	-0,32	FOXJ1	forkhead box J1 [Source:HGNC Symbol;Acc:3816] [ENSECAT00000014984]	TAGACTTTGACGAGACCTTCCTGGCCACGTCCTTCCTGCAGCACCCCTGGGACGAGAGTG
619	A_69_P081261	0,04	0,00	up	2,26	2,26	1,18	LOC100630858	Metallothionein [Source:UniProtKB/TrEMBL;Acc:F6VKN2] [ENSECAT00000001640]	TCCTGCAAATGCAAAGAGTGCAGATGCACTTCCTGCAAGAAGAGCTGCTGCCCCTGCTGC
620	A_69_P108681	0,04	0,00	down	1,45	-1,45	-0,53		TSA: Equus caballus contig23102.EqcaPBMC mRNA sequence. [JL637774]	TGTCCTTCCAAAAATGTTGTGTACAAACCATGCTTTCAATGTTGGCCTCCAAGTTTTTTA

621	A_69_P000613	0,04	0,00	up	1,17	1,17	0,23	SOD2	Equus caballus superoxide dismutase 2, mitochondrial (SOD2), mRNA [NM_001082517]	GGGAGAATGTATCTGAGAGATACATGGCTTGCAAAAGTAAAGCATTATCGTTACACCGA
622	A_69_P129291	0,04	0,00	up	1,27	1,27	0,34	SLC16A8	PREDICTED: Equus caballus solute carrier family 16 (monocarboxylate transporter), member 8 (SLC16A8), mRNA [XM_001499671]	AGATCATCTTCTACCTGGCCGGCTCTGAGGTGGTCCTCGCAGGGGTCTTCATGGCTGTGG
623	A_69_P000611	0,04	0,00	up	1,29	1,29	0,36	SOD2	Equus caballus superoxide dismutase 2, mitochondrial (SOD2), mRNA [NM_001082517]	AAGCATTATCGTTACACCGAGTGCATTAAGCAATTTCCAACTATTTTTGTAGTAGCTCGA
624	A_69_P065075	0,04	0,00	up	1,37	1,37	0,45	POP5	processing of precursor 5, ribonuclease P/MRP subunit (S. cerevisiae) [Source:HGNC Symbol;Acc:17689] [ENSECAT00000010367]	ACATGTCAGAAGTTCCTGATTCAGTACAACCGGAGACAGCTGTTGATCTTGTTGCAACAC
625	A_69_P102959	0,04	0,00	down	1,12	-1,12	-0,17		LeukoN4_2_B02.b1_A026 Unstimulated peripheral blood leukocytes N4 Equus caballus cDNA clone LeukoN4_2_B02_A026 3', mRNA sequence [CD464177]	TGCCTGGGAGGAGATGAAGATGAGGAGGTTTTTCCTTACTGTATAAATGAATATTTGTAT
626	A_69_P033871	0,05	0,00	up	1,37	1,37	0,45	RSAD1	radical S-adenosyl methionine domain containing 1 [Source:HGNC Symbol;Acc:25634] [ENSECAT00000024928]	TTTGAGCCCCAGCTGAACCTTTGGGACGTGTTTGGAGCAAGCACAGAGGTGAAGGTGCTG
627	A_69_P111264	0,05	0,00	down	1,31	-1,31	-0,39		CT02037B2G01 Equine Articular Cartilage cDNA Library Equus caballus cDNA clone CT02037B2G01, mRNA sequence [CX604288]	TATTCAAGTTCAAATGATGAACTTCCAGACTCTGGTGACTCATACTTCCCAGAGCCGACC
