# Supplementary material for: Characterisation of equine satellite cell transcriptomic profile response to β-hydroxy-β-methylbutyrate (HMB)
Source: Br J Nutr. 2016 Oct 3;116(8):1315–25. doi: 10.1017/S000711451600324X (PMC5082287; doi:10.1017/S000711451600324X)
Supplement: Supplementary file 1 [file S000711451600324Xsup.zip › S000711451600324Xsup001/S000711451600324Xsup002.pdf]

Supplementary material 2

DAVID Biological Processes

Category	Term	Count	%	PValue	Genes	List Total	Pop Hits	Pop Total	Fold Enrich	Bonferroni	Benjamini	FDR
GOTERM_BP_ALL	GO:0007517~muscle organ development	14	4.12	0.00	MEF2C, MEF2A, MYF5, TAGLN3, TGFB2, LAMA2, ZFP91, MURC, LAMA5, MAPK14, SVIL, DMD, ITGB1BP2, F2R	273	211	14116	3.43	0.37	0.37	0.4
GOTERM_BP_ALL	GO:0009987~cellular process	227	66.76	0.00	MEF2C, MEF2A, ALAD, TARS2, FST, GFER, LPAR2, EDIL3, REST, TPD52, PRKG1, S1PR2, CUL3, ZFP91, HMCN1, KIFAP3, SFRS9, SCD5, NSMAF, RPP21, COLEC12, PPARGC1B, GRB10, RPS18, ZNF238, RPS14, UBR5, ZNF786, RPS12, TRAPPC4, MRPL48, HSD17B11, CHKA, GGCT, NHP2L1, STK17B, HOXB13, SFXN2, SYPL2, KCNMB2, ITGBL1, RPS26, TRAM2, MACF1, DUSP13, ARG2, DMD, NKX2-2, SPP1, GPD1, ESRRA, TP53BP1, PHB, MCAT, SMAD5, KLF11, TPD52L1, SMAD1, MCAM, CDC27, LAMA2, LAMA5, MLX, SYTL2, GK, POP5, SLC46A2, STEAP3, KCNH1, KIAA0368, TTLL1, GLI1, KCNIP3, BAG1, MAP1LC3B, SAA1, SLMAP, GUCY1A3, RAB6B, NOS2, IP6K2, KDELR2, PCDHB7, TBX15, BRF2, MMP15, MTMR11, GNB1, PROK1, GRM6, FARSB, ZSCAN16, SUSD5, TOMM22, SEMA4D, BCAT1, ABHD5, PCDHB14, ZFP36L2, PRKAA1, RPIA, USP31, FADS1, MYF5, RPL29, SOD2, PHF17, CSNK1E, NHEDC1, MAPK14, SVIL, SLC16A8, FBLN7, DPYD, SPG11, ABCC8, USP45, RBM17, CALM1, SLC8A3, GNPTG, TSPO, ILKAP, TLR1, GNA12, OSGIN2, PMAIP1, SLC7A7, TGFB2, MLL5, SLC2A5, AAK1, ST3GAL6, SLC25A28, STK39, ROBO3, GABPB2, SPON1, NUDT1, NCALD, PRKAB2, MGP, TMSB10, PPP1CC, CDO1, RSL1D1, CPE, VAMP8, VAMP1, SMARCA2, ASB7, PRPS2, SSH2, BEX1, UBE2V1, ABCA1, CCNG1, KCNS1, B3GNT5, METTL1, PTPLA, HNRNPD, EIF3I, DNAJA4, B4GALT7, RHOBTB3, B4GALT1, TAF5, MRPS24, SMG1, TRIM63, MAFK, ABCB6, UACA, MTR, HOXB9, CPNE3, SPCS1, SYT17, CDK19, FOSL2, NDST2, MTSS1L, PEX3, RPS3, ANG, PAK3, NPC1L1, PSMD6, PLCB1, KCNQ1, VCPIP1, KIF2A, FOXJ1, COX4I1, ARHGAP27, PCDH9, IPO9, PIGN, EML4, MURC, CHRM3, MFSD10, CA4, PTAFR, KPNA1, PEG3, DPF2, TNFRSF25, ZNF805, PUSL1, RSAD1, TPM3, CHD7, AP3M1, PRDM10, NFATC3, COL8A2, GCHFR, SLC2A9, UBE4A, RNASE6, MAL, AMDHD2, SLC17A5, ALKBH3, F2R	273	10541	14116	1.11	0.66	0.42	0.91
GOTERM_BP_ALL	GO:0048518~positive regulation of biological process	58	17.06	0.00	MEF2C, FOSL2, FST, TLR1, LPAR2, PMAIP1, EDIL3, GLI1, TGFB2, RPS3, CUL3, S1PR2, ZFP91, MLL5, ANG, SAA1, KIFAP3, GUCY1A3, NOS2, PSMD6, SAMD4A, IP6K2, FOXJ1, PPARGC1B, MURC, CHRM3, PROK1, SEMA4D, SMARCA2, DPF2, TBC1D8, TNFRSF25, ABHD5, STK17B, UBE2V1, ABCA1, CHD7, PRKAA1, RNF10, NFATC3, NKX2-2, SPP1, B4GALT1, ESRRA, TP53BP1, MYF5, SMAD5, MAL, SMAD1, CDC27, TAB2, SOD2, LAMA2, UACA, MAPK14, HOXB9, F2R, CALM1	273	2033	14116	1.48	0.98	0.73	3.3
GOTERM_BP_ALL	GO:0050793~regulation of developmental process	25	7.35	0.00	GNA12, FST, ABCA1, REST, GLI1, TGFB2, ZFP91, CDC42EP3, NKX2-2, SPP1, B4GALT1, ESRRA, FOXJ1, FADS1, SMAD5, MGP, SKI, SMAD1, SOD2, LAMA2, LAMA5, PROK1, MAPK14, GDF10, SEMA4D	273	674	14116	1.92	1.00	0.76	4.75

GOTERM_BP_ALL	GO:0044267~cellular protein metabolic process	64	18.82	0.00	GNPTG, CDK19, ILKAP, TARS2, KIAA0368, LPAR2, PRKG1, TTLL1, TGFB2, RPS3, S1PR2, CUL3, MLL5, HMCN1, PAK3, MAP1LC3B, AAK1, SLMAP, ST3GAL6, STK39, PSMD6, VCPIP1, MMP15, PPP1CC, PIGN, RSL1D1, RPS18, CHRM3, CPE, PROK1, UBR5, RPS14, RPS12, FARSB, MRPL48, ASB7, SSH2, STK17B, UBE2V1, ABCA1, RPS26, B3GNT5, DUSP13, PTPLA, PRKAA1, EIF3I, DNAJA4, B4GALT7, USP31, B4GALT1, UBE4A, PHB, MRPS24, SMG1, TRIM63, CDC27, RPL29, PHF17, CSNK1E, MAPK14, MTR, SPCS1, USP45, F2R	273	2355	14116	1.41	1.00	0.75	5.65
GOTERM_BP_ALL	GO:0030278~regulation of ossification	7	2.06	0.00	ESRRA, SMAD5, MGP, GDF10, SKI, SMAD1, TGFB2	273	78	14116	4.64	1.00	0.73	6.51
GOTERM_BP_ALL	GO:0051239~regulation of multicellular organismal process	31	9.12	0.00	TLR1, FST, REST, TPM3, KCNMB2, TGFB2, GLI1, ZFP91, CHD7, SAA1, ARG2, GUCY1A3, NOS2, KCNQ1, NKX2-2, SPP1, B4GALT1, ESRRA, FOXJ1, SMAD5, MGP, SKI, SMAD1, LAMA2, CHRM3, LAMA5, PROK1, MAPK14, GDF10, SEMA4D, F2R	273	937	14116	1.71	1.00	0.71	7.11
GOTERM_BP_ALL	GO:0030155~regulation of cell adhesion	9	2.65	0.01	LAMA2, CYTIP, SAA1, LAMA5, KIFAP3, MYF5, EDIL3, SPP1, TGFB2	273	137	14116	3.4	1.00	0.72	8.35
GOTERM_BP_ALL	GO:0048522~positive regulation of cellular process	51	15.00	0.01	MEF2C, FOSL2, TLR1, LPAR2, PMAIP1, EDIL3, TGFB2, GLI1, RPS3, CUL3, S1PR2, ZFP91, MLL5, SAA1, ANG, KIFAP3, GUCY1A3, PSMD6, SAMD4A, IP6K2, PPARGC1B, MURC, PROK1, SEMA4D, SMARCA2, DPF2, TBC1D8, TNFRSF25, ABHD5, STK17B, UBE2V1, ABCA1, RNF10, NFATC3, NKX2-2, SPP1, B4GALT1, ESRRA, TP53BP1, MYF5, SMAD5, MAL, SMAD1, TAB2, CDC27, SOD2, LAMA2, UACA, MAPK14, HOXB9, F2R	273	1847	14116	1.43	1.00	0.82	12.3
GOTERM_BP_ALL	GO:0051345~positive regulation of hydrolase activity	10	2.94	0.01	UACA, ANG, GNB1, FOXJ1, ABHD5, ARHGAP27, LPAR2, PMAIP1, RPS3, F2R	273	179	14116	2.89	1.00	0.8	12.83
GOTERM_BP_ALL	GO:0009891~positive regulation of biosynthetic process	24	7.06	0.01	MEF2C, ESRRA, TP53BP1, MYF5, SMAD5, TLR1, ABCA1, SMAD1, PPARGC1B, SOD2, GLI1, TGFB2, MURC, MLL5, MAPK14, GUCY1A3, PRKAA1, HOXB9, RNF10, SMARCA2, NFATC3, NKX2-2, SAMD4A, F2R	273	695	14116	1.79	1.00	0.78	13.21
GOTERM_BP_ALL	GO:0060341~regulation of cellular localization	12	3.53	0.01	B4GALT1, ZFP91, UACA, CHD7, SAA1, ANG, PKIG, FST, NOS2, KCNQ1, CALM1, TGFB2	273	248	14116	2.5	1.00	0.78	14.1
GOTERM_BP_ALL	GO:0016053~organic acid biosynthetic process	9	2.65	0.01	BCAT1, FADS1, ABHD5, PRKAB2, MTR, MCAT, PRKAA1, SCD5, CDO1	273	155	14116	3,00	1.00	0.8	16.43
GOTERM_BP_ALL	GO:0046394~carboxylic acid biosynthetic process	9	2.65	0.01	BCAT1, FADS1, ABHD5, PRKAB2, MTR, MCAT, PRKAA1, SCD5, CDO1	273	155	14116	3,00	1.00	0.8	16.43
GOTERM_BP_ALL	GO:0009893~positive regulation of metabolic process	29	8.53	0.01	MEF2C, ABHD5, TLR1, ABCA1, GLI1, TGFB2, ZFP91, MLL5, ANG, GUCY1A3, RNF10, PRKAA1, PSMD6, NFATC3, NKX2-2, SAMD4A, ESRRA, TP53BP1, MYF5, SMAD5, SMAD1, CDC27, PPARGC1B, SOD2, MURC, MAPK14, HOXB9, SMARCA2, F2R	273	921	14116	1.63	1.00	0.8	17.53
GOTERM_BP_ALL	GO:0042060~wound healing	10	2.94	0.01	B4GALT1, F2RL2, HMCN1, SAA1, GNA12, SERPIND1, SMAD1, SYT17, F2R, TGFB2	273	191	14116	2.71	1.00	0.8	18.72
GOTERM_BP_ALL	GO:0006813~potassium ion transport	9	2.65	0.01	KCNH1, KCNS1, KCNK12, ABCC8, KCNQ1, KCTD7, KCNMB2, KCNIP3, TMEM38B	273	160	14116	2.91	1.00	0.79	19.32
GOTERM_BP_ALL	GO:0048585~negative regulation of response to stimulus	7	2.06	0.01	GRB10, UACA, SAA1, FOXJ1, RPS3, SPP1, TGFB2	273	100	14116	3.62	1.00	0.78	19.88

GOTERM_BP_ALL	GO:0043412~biopolymer modification	42	12.35	0.02	GNPTG, CDK19, ILKAP, PUSL1, SSH2, STK17B, UBE2V1, LPAR2, ABCA1, PRKG1, TTLL1, TGFB2, S1PR2, MLL5, HMCN1, B3GNT5, DUSP13, PAK3, AAK1, METTL1, ST3GAL6, PTPLA, PRKAA1, STK39, B4GALT7, VCPIP1, B4GALT1, UBE4A, PHB, SMG1, MMP15, PPP1CC, PIGN, PHF17, CHRM3, CPE, CSNK1E, PROK1, MAPK14, UBR5, MTR, F2R	273	1526	14116	1.42	1.00	0.86	26.21
GOTERM_BP_ALL	GO:0042127~regulation of cell proliferation	25	7.35	0.02	DLEC1, FOSL2, TBC1D8, TGFB2, GLI1, S1PR2, CUL3, ZFP91, ANG, KIFAP3, NOS2, B4GALT7, GNL3, B4GALT1, ESRRA, FOXJ1, PHB, KLF11, SKI, SMAD1, SOD2, LAMA5, PROK1, SMARCA2, F2R	273	787	14116	1.64	1.00	0.85	26.21
GOTERM_BP_ALL	GO:0031325~positive regulation of cellular metabolic process	27	7.94	0.02	MEF2C, ABHD5, TLR1, ABCA1, GLI1, ZFP91, MLL5, ANG, GUCY1A3, RNF10, PSMD6, NFATC3, NKX2-2, SAMD4A, ESRRA, TP53BP1, SMAD5, MYF5, SMAD1, CDC27, PPARGC1B, SOD2, MURC, MAPK14, HOXB9, SMARCA2, F2R	273	880	14116	1.59	1.00	0.87	28.99
GOTERM_BP_ALL	GO:0006464~protein modification process	40	11.76	0.02	GNPTG, CDK19, ILKAP, SSH2, STK17B, UBE2V1, LPAR2, ABCA1, PRKG1, TTLL1, TGFB2, S1PR2, MLL5, HMCN1, B3GNT5, DUSP13, PAK3, AAK1, ST3GAL6, PTPLA, PRKAA1, STK39, B4GALT7, VCPIP1, B4GALT1, UBE4A, PHB, SMG1, MMP15, PPP1CC, PIGN, PHF17, CHRM3, CPE, CSNK1E, PROK1, MAPK14, UBR5, MTR, F2R	273	1453	14116	1.42	1.00	0.87	30.18
GOTERM_BP_ALL	GO:0006809~nitric oxide biosynthetic process	3	0.88	0.02	ARG2, NOS2, GCHFR	273	12	14116	12.93	1.00	0.87	31.22
GOTERM_BP_ALL	GO:0048856~anatomical structure development	63	18.53	0.02	MEF2C, KCNH1, MEF2A, FST, GNA12, REST, PRKG1, TPD52, TGFB2, GLI1, CUL3, ZFP91, MLL5, BAG1, ANG, CCBE1, NOS2, ROBO3, KIF2A, TBX15, CA10, FOXJ1, MGP, CDO1, MURC, CHRM3, PROK1, RPS14, ITGB1BP2, TRAPPC4, CA4, SEMA4D, BEX1, PCDHB14, HOXB13, TAGLN3, CHD7, KRT27, MACF1, B3GNT5, DMD, NFATC3, COL8A2, NKX2-2, SPP1, B4GALT1, ESRRA, MYF5, SMAD5, MAL, SKI, SMAD1, MAFK, MCAM, SOD2, LAMA2, LAMA5, MAPK14, SVIL, MTR, GDF10, HOXB9, F2R	273	2527	14116	1.29	1.00	0.87	32.76
GOTERM_BP_ALL	GO:0008285~negative regulation of cell proliferation	14	4.12	0.02	B4GALT1, DLEC1, FOXJ1, PHB, KLF11, SKI, SMAD1, SOD2, TGFB2, ANG, KIFAP3, SMARCA2, B4GALT7, F2R	273	361	14116	2.01	1.00	0.86	33.01
GOTERM_BP_ALL	GO:0006605~protein targeting	10	2.94	0.02	GNPTG, TSPO, MACF1, AP3M1, IPO9, TOMM22, PEX3, KPNA1, F2R, TGFB2	273	215	14116	2.4	1.00	0.86	33.86
GOTERM_BP_ALL	GO:0009058~biosynthetic process	84	24.71	0.02	GNPTG, MEF2C, TSPO, ALAD, MEF2A, TARS2, NDST2, REST, RPS3, KCNIP3, GLI1, TGFB2, ZFP91, MLL5, ANG, ST3GAL6, NPC1L1, GUCY1A3, NOS2, SCD5, GABPB2, TBX15, BRF2, FOXJ1, PRKAB2, CDO1, PPARGC1B, PIGN, RSL1D1, MURC, RPS18, ZNF238, ZNF786, RPS14, FARSB, RPS12, ZSCAN16, MRPL48, SMARCA2, PRPS2, PTAFR, PEG3, HSD17B11, BCAT1, DPF2, CHKA, ZNF805, ABHD5, HOXB13, RSAD1, ABCA1, RPS26, TRAM2, CHD7, B3GNT5, ARG2, DMD, PRDM10, HNRNPD, PRKAA1, EIF3I, NFATC3, B4GALT7, GCHFR, B4GALT1, GPD1, ESRRA, TAF5, FADS1, TP53BP1, PHB, MCAT, KLF11, MRPS24, SMAD5, SMAD1, MAFK, RPL29, SOD2, PHF17, MLX, MTR, HOXB9, DPYD	273	3542	14116	1.23	1.00	0.85	33.9
GOTERM_BP_ALL	GO:0044271~nitrogen compound biosynthetic process	13	3.82	0.02	BCAT1, ALAD, TSPO, RSAD1, CDO1, TGFB2, ARG2, MTR, GUCY1A3, DPYD, NOS2, PRPS2, GCHFR	273	325	14116	2.07	1.00	0.84	34.07
GOTERM_BP_ALL	GO:0006357~regulation of transcription from RNA polymerase II promoter	23	6.76	0.02	MEF2C, ESRRA, FOSL2, FOXJ1, MYF5, SMAD5, KLF11, FST, PKIG, SKI, SMAD1, PPARGC1B, SOD2, GLI1, KCNIP3, MURC, ZNF238, MAPK14, RPS14, HOXB9, SMARCA2, NFATC3, NKX2-2	273	727	14116	1.64	1.00	0.83	34.38

GOTERM_BP_ALL	GO:0031328~positive regulation of cellular biosynthetic process	22	6.47	0.02	MEF2C, ESRRA, TP53BP1, MYF5, SMAD5, TLR1, ABCA1, SMAD1, PPARGC1B, SOD2, GLI1, MURC, MLL5, MAPK14, GUCY1A3, HOXB9, RNF10, SMARCA2, NFATC3, NKX2-2, SAMD4A, F2R	273	685	14116	1.66	1.00	0.82	34.77
GOTERM_BP_ALL	GO:0046209~nitric oxide metabolic process	3	0.88	0.03	ARG2, NOS2, GCHFR	273	13	14116	11.93	1.00	0.82	35.44
GOTERM_BP_ALL	GO:0044237~cellular metabolic process	145	42.65	0.03	MEF2C, GNPTG, MEF2A, ILKAP, TSPO, ALAD, TARS2, LPAR2, REST, PRKG1, SLC7A7, TGFB2, S1PR2, CUL3, ZFP91, MLL5, HMCN1, AAK1, ST3GAL6, SFRS9, STK39, SCD5, NSMAF, GABPB2, NUDT1, RPP21, PRKAB2, CDO1, PPP1CC, PPARGC1B, RSL1D1, RPS18, ZNF238, CPE, ZNF786, RPS14, UBR5, RPS12, MRPL48, SMARCA2, ASB7, PRPS2, HSD17B11, CHKA, NHP2L1, SSH2, STK17B, HOXB13, UBE2V1, ABCA1, RPS26, DUSP13, B3GNT5, METTL1, ARG2, DMD, PTPLA, HNRNPD, EIF3I, DNAJA4, B4GALT7, B4GALT1, GPD1, ESRRA, TAF5, TP53BP1, PHB, MCAT, SMAD5, KLF11, MRPS24, SMG1, SMAD1, MAFK, TRIM63, CDC27, MLX, MTR, HOXB9, SPCS1, GK, POP5, CDK19, KIAA0368, NDST2, TTLL1, KCNIP3, RPS3, GLI1, MAP1LC3B, ANG, PAK3, SLMAP, NPC1L1, GUCY1A3, NOS2, PSMD6, PLCB1, KCNQ1, VCPIP1, IP6K2, TBX15, BRF2, FOXJ1, COX4I1, MMP15, PIGN, MURC, MTMR11, CHRM3, PROK1, FARSB, CA4, ZSCAN16, PTAFR, PEG3, BCAT1, DPF2, ZNF805, PUSL1, ABHD5, RSAD1, ZFP36L2, CHD7, PRDM10, PRKAA1, RPIA, NFATC3, GCHFR, USP31, UBE4A, FADS1, RNASE6, SOD2, RPL29, PHF17, AMDHD2, CSNK1E, MAPK14, SLC16A8, DPYD, ALKBH3, USP45, RBM17, F2R	273	6636	14116	1.13	1.00	0.84	38.34
GOTERM_BP_ALL	GO:0032879~regulation of localization	20	5.88	0.03	B4GALT1, TSPO, ABHD5, FST, PKIG, ABCA1, TGFB2, LAMA2, RSL1D1, ZFP91, GRB10, CHD7, UACA, SAA1, ANG, LAMA5, NOS2, KCNQ1, CALM1, F2R	273	610	14116	1.7	1.00	0.83	38.5
GOTERM_BP_ALL	GO:0045785~positive regulation of cell adhesion	5	1.47	0.03	SAA1, KIFAP3, EDIL3, SPP1, TGFB2	273	60	14116	4.31	1.00	0.83	39.04
GOTERM_BP_ALL	GO:0045595~regulation of cell differentiation	17	5.0	0.03	B4GALT1, ESRRA, FOXJ1, FADS1, SMAD5, FST, SKI, ABCA1, SMAD1, REST, SOD2, TGFB2, ZFP91, MAPK14, SEMA4D, NKX2-2, SPP1	273	492	14116	1.79	1.00	0.83	39.91
GOTERM_BP_ALL	GO:0051336~regulation of hydrolase activity	13	3.82	0.03	TBC1D8, FOXJ1, ABHD5, ARHGAP27, LPAR2, PMAIP1, RPS3, TGFB2, UACA, GNB1, ANG, GCHFR, F2R	273	337	14116	1.99	1.00	0.83	41.36
GOTERM_BP_ALL	GO:0048731~system development	58	17.06	0.03	MEF2C, MEF2A, FST, REST, PRKG1, TPD52, TGFB2, GLI1, ZFP91, MLL5, BAG1, ANG, CCBE1, ROBO3, NOS2, KIF2A, TBX15, CA10, FOXJ1, MGP, CDO1, MURC, CHRM3, PROK1, RPS14, ITGB1BP2, TRAPPC4, CA4, SEMA4D, BEX1, PCDHB14, HOXB13, TAGLN3, CHD7, KRT27, MACF1, B3GNT5, DMD, COL8A2, NFATC3, NKX2-2, SPP1, B4GALT1, ESRRA, MYF5, SMAD5, MAL, SMAD1, MAFK, SOD2, LAMA2, LAMA5, MAPK14, SVIL, MTR, GDF10, HOXB9, F2R	273	2330	14116	1.29	1.00	0.83	41.43
GOTERM_BP_ALL	GO:0065009~regulation of molecular function	28	8.24	0.03	TBC1D8, GNAI1, ABHD5, UBE2V1, LPAR2, PMAIP1, CCNG1, NPRL2, RPS3, TGFB2, S1PR2, ANG, ARG2, PRKAA1, PSMD6, GCHFR, FOXJ1, PKIG, ARHGAP27, SKI, CDC27, SOD2, UACA, GNB1, PROK1, GRM6, CALM1, F2R	273	969	14116	1.49	1.00	0.85	45.08
GOTERM_BP_ALL	GO:0044093~positive regulation of molecular function	19	5.59	0.03	FOXJ1, ABHD5, UBE2V1, ARHGAP27, SKI, LPAR2, PMAIP1, CDC27, RPS3, TGFB2, S1PR2, UACA, GNB1, ANG, PROK1, PRKAA1, PSMD6, CALM1, F2R	273	586	14116	1.68	1.00	0.85	45.51
GOTERM_BP_ALL	GO:0050790~regulation of catalytic activity	25	7.35	0.04	TBC1D8, GNAI1, ABHD5, LPAR2, PMAIP1, CCNG1, NPRL2, RPS3, TGFB2, S1PR2, ANG, ARG2, PRKAA1, PSMD6, GCHFR, FOXJ1, PKIG, ARHGAP27, CDC27, SOD2, UACA, GNB1, PROK1, GRM6, F2R	273	846	14116	1.53	1.00	0.86	47.82

GOTERM_BP_ALL	GO:0046486~glycerolipid metabolic process	8	2.35	0.04	CHKA, GPD1, ANG, ABHD5, SMG1, PLCB1, IP6K2, PIGN	273	162	14116	2.55	1.00	0.86	48.24
GOTERM_BP_ALL	GO:0008219~cell death	22	6.47	0.04	MEF2C, DPF2, STEAP3, MEF2A, TSPO, FOSL2, GGCT, TNFRSF25, KLF11, STK17B, PMAIP1, RPS3, SOD2, TGFB2, KCNIP3, PHF17, BAG1, ANG, MFSD10, SPG11, F2R, PEG3	273	719	14116	1.58	1.00	0.85	48.65
GOTERM_BP_ALL	GO:0060538~skeletal muscle organ development	5	1.47	0.04	SVIL, MAPK14, DMD, MYF5, F2R	273	66	14116	3.92	1.00	0.85	48.99
GOTERM_BP_ALL	GO:0007519~skeletal muscle tissue development	5	1.47	0.04	SVIL, MAPK14, DMD, MYF5, F2R	273	66	14116	3.92	1.00	0.85	48.99
GOTERM_BP_ALL	GO:0046883~regulation of hormone secretion	5	1.47	0.04	ZFP91, CHD7, FST, NOS2, KCNQ1	273	66	14116	3.92	1.00	0.85	48.99
GOTERM_BP_ALL	GO:0019637~organophosphate metabolic process	9	2.65	0.04	CHKA, GPD1, FADS1, ABHD5, SMG1, GK, PLCB1, IP6K2, PIGN	273	200	14116	2.33	1.00	0.85	50.44
GOTERM_BP_ALL	GO:0016265~death	22	6.47	0.04	MEF2C, DPF2, STEAP3, MEF2A, TSPO, FOSL2, GGCT, TNFRSF25, KLF11, STK17B, PMAIP1, RPS3, SOD2, TGFB2, KCNIP3, PHF17, BAG1, ANG, MFSD10, SPG11, F2R, PEG3	273	724	14116	1.57	1.00	0.85	50.97
GOTERM_BP_ALL	GO:0017038~protein import	7	2.06	0.04	TSPO, IPO9, TOMM22, PEX3, KPNA1, F2R, TGFB2	273	131	14116	2.76	1.00	0.85	51.59
GOTERM_BP_ALL	GO:0022604~regulation of cell morphogenesis	7	2.06	0.04	ZFP91, GNA12, SEMA4D, SMAD1, CDC42EP3, SPP1, TGFB2	273	131	14116	2.76	1.00	0.85	51.59
GOTERM_BP_ALL	GO:0051046~regulation of secretion	9	2.65	0.04	B4GALT1, ZFP91, CHD7, SAA1, ANG, FST, NOS2, KCNQ1, TGFB2	273	202	14116	2.3	1.00	0.85	52.4
GOTERM_BP_ALL	GO:0051049~regulation of transport	15	4.41	0.04	B4GALT1, TSPO, FST, PKIG, TGFB2, ZFP91, GRB10, CHD7, UACA, ANG, SAA1, NOS2, KCNQ1, CALM1, F2R	273	435	14116	1.78	1.00	0.85	53.09
GOTERM_BP_ALL	GO:0045893~positive regulation of transcription, DNA-dependent	16	4.71	0.04	MEF2C, ESRRA, TP53BP1, SMAD5, MYF5, SMAD1, PPARGC1B, GLI1, MURC, MLL5, MAPK14, HOXB9, SMARCA2, NFATC3, NKX2-2, F2R	273	477	14116	1.73	1.00	0.85	53.21
GOTERM_BP_ALL	GO:0044249~cellular biosynthetic process	80	23.53	0.04	GNPTG, MEF2C, TSPO, ALAD, MEF2A, TARS2, NDST2, REST, RPS3, KCNIP3, GLI1, TGFB2, ZFP91, MLL5, ANG, ST3GAL6, GUCY1A3, NOS2, SCD5, GABPB2, TBX15, BRF2, FOXJ1, PRKAB2, CDO1, PPARGC1B, PIGN, RSL1D1, MURC, RPS18, ZNF238, ZNF786, RPS14, FARSB, RPS12, ZSCAN16, MRPL48, SMARCA2, PRPS2, PTAFR, PEG3, BCAT1, DPF2, CHKA, ZNF805, ABHD5, HOXB13, RSAD1, ABCA1, RPS26, CHD7, B3GNT5, ARG2, DMD, PRDM10, HNRNPD, PRKAA1, EIF3I, NFATC3, GCHFR, B4GALT1, GPD1, ESRRA, TAF5, FADS1, PHB, TP53BP1, KLF11, MRPS24, SMAD5, MCAT, SMAD1, MAFK, RPL29, SOD2, PHF17, MLX, MTR, HOXB9, DPYD	273	3442	14116	1.2	1.00	0.84	53.24
GOTERM_BP_ALL	GO:0048519~negative regulation of biological process	46	13.53	0.04	MEF2C, DLEC1, ILKAP, ABHD5, FST, ABCA1, REST, RPS3, KCNIP3, TGFB2, ZFP91, RPS26, BAG1, ANG, SAA1, KIFAP3, ARG2, GUCY1A3, PRKAA1, PSMD6, KCNQ1, B4GALT7, GCHFR, NKX2-2, SPP1, IP6K2, B4GALT1, FOXJ1, PHB, KLF11, PKIG, SKI, TMSB10, SMAD1, CDC27, SOD2, PHF17, GRB10, UACA, ZNF238, MLX, RPS14, SEMA4D, SMARCA2, CALM1, F2R	273	1812	14116	1.31	1.00	0.83	53.24
GOTERM_BP_ALL	GO:0051173~positive regulation of nitrogen compound metabolic process	20	5.88	0.04	MEF2C, ESRRA, TP53BP1, MYF5, SMAD5, ABCA1, SMAD1, PPARGC1B, SOD2, GLI1, MURC, MLL5, MAPK14, GUCY1A3, HOXB9, RNF10, SMARCA2, NFATC3, NKX2-2, F2R	273	644	14116	1.61	1.00	0.83	53.47

GOTERM_BP_ALL	GO:0019538~protein metabolic process	67	19.71	0.04	GNPTG, CDK19, ILKAP, TARS2, KIAA0368, LPAR2, PRKG1, TTLL1, RPS3, TGFB2, S1PR2, CUL3, MLL5, HMCN1, PAK3, MAP1LC3B, AAK1, SLMAP, ST3GAL6, STK39, PSMD6, VCPIP1, MMP15, PPP1CC, PIGN, RSL1D1, RPS18, CHRM3, CPE, PROK1, UBR5, RPS14, RPS12, FARSB, MRPL48, ASB7, SSH2, STK17B, UBE2V1, ABCA1, RPS26, B3GNT5, DUSP13, DISP1, PTPLA, PRKAA1, EIF3I, DNAJA4, B4GALT7, USP31, CAPN6, B4GALT1, UBE4A, PHB, MRPS24, TMPRSS9, SMG1, TRIM63, CDC27, RPL29, PHF17, CSNK1E, MAPK14, MTR, SPCS1, USP45, F2R	273	2812	14116	1.23	1.00	0.83	53.77
GOTERM_BP_ALL	GO:0001501~skeletal system development	12	3.53	0.05	ESRRA, TBX15, CHD7, MAPK14, MYF5, SMAD5, MGP, GDF10, HOXB9, SMAD1, GLI1, SPP1	273	319	14116	1.95	1.00	0.83	54.97
GOTERM_BP_ALL	GO:0006414~translational elongation	6	1.76	0.05	RPS26, RPS18, RPS14, RPS12, RPS3, RPL29	273	101	14116	3.07	1.00	0.83	55.24
GOTERM_BP_ALL	GO:0051254~positive regulation of RNA metabolic process	16	4.71	0.05	MEF2C, ESRRA, TP53BP1, SMAD5, MYF5, SMAD1, PPARGC1B, GLI1, MURC, MLL5, MAPK14, HOXB9, SMARCA2, NFATC3, NKX2-2, F2R	273	481	14116	1.72	1.00	0.83	55.7
GOTERM_BP_ALL	GO:0048732~gland development	7	2.06	0.05	B4GALT1, LAMA5, HOXB13, HOXB9, CDO1, GLI1, TGFB2	273	135	14116	2.68	1.00	0.82	56.03
GOTERM_BP_ALL	GO:0001942~hair follicle development	4	1.18	0.05	KRT27, LAMA5, FST, TGFB2	273	42	14116	4.92	1.00	0.82	56.21
GOTERM_BP_ALL	GO:0001649~osteoblast differentiation	4	1.18	0.05	SMAD5, SMAD1, GLI1, SPP1	273	42	14116	4.92	1.00	0.82	56.21
GOTERM_BP_ALL	GO:0022404~molting cycle process	4	1.18	0.05	KRT27, LAMA5, FST, TGFB2	273	42	14116	4.92	1.00	0.82	56.21
GOTERM_BP_ALL	GO:0022405~hair cycle process	4	1.18	0.05	KRT27, LAMA5, FST, TGFB2	273	42	14116	4.92	1.00	0.82	56.21
GOTERM_BP_ALL	GO:0050817~coagulation	6	1.76	0.05	F2RL2, HMCN1, SAA1, GNA12, SERPIND1, F2R	273	102	14116	3.04	1.00	0.82	56.54
GOTERM_BP_ALL	GO:0007596~blood coagulation	6	1.76	0.05	F2RL2, HMCN1, SAA1, GNA12, SERPIND1, F2R	273	102	14116	3.04	1.00	0.82	56.54
GOTERM_BP_ALL	GO:0051253~negative regulation of RNA metabolic process	13	3.82	0.05	MEF2C, FOXJ1, PKIG, KLF11, FST, SKI, REST, KCNIP3, RPS26, ZNF238, RPS14, MLX, SMARCA2	273	362	14116	1.86	1.00	0.82	57.33
GOTERM_BP_ALL	GO:0044255~cellular lipid metabolic process	17	5.0	0.05	CHKA, GPD1, FADS1, MCAT, ABHD5, PRKAB2, SMG1, PIGN, B3GNT5, ANG, MAPK14, ST3GAL6, PRKAA1, SCD5, NSMAF, PLCB1, IP6K2	273	526	14116	1.67	1.00	0.81	57.51
GOTERM_BP_ALL	GO:0010557~positive regulation of macromolecule biosynthetic process	20	5.88	0.05	MEF2C, ESRRA, TP53BP1, MYF5, SMAD5, TLR1, SMAD1, PPARGC1B, GLI1, TGFB2, MURC, MLL5, MAPK14, HOXB9, RNF10, SMARCA2, NFATC3, NKX2-2, SAMD4A, F2R	273	654	14116	1.58	1.00	0.81	57.53
GOTERM_BP_ALL	GO:0008610~lipid biosynthetic process	12	3.53	0.05	HSD17B11, CHKA, B3GNT5, ANG, FADS1, ABHD5, PRKAB2, MCAT, NPC1L1, PRKAA1, SCD5, PIGN	273	323	14116	1.92	1.00	0.8	57.62
GOTERM_BP_ALL	GO:0007178~transmembrane receptor protein serine/threonine kinase signaling pathway	6	1.76	0.05	FST, SMAD5, GDF10, SKI, SMAD1, TGFB2	273	103	14116	3.01	1.00	0.8	57.83
GOTERM_BP_ALL	GO:0009605~response to external stimulus	26	7.65	0.05	F2RL2, TLR1, GNA12, HOXB13, ENSA, TGFB2, MLL5, HMCN1, SAA1, ROBO3, NFATC3, SPP1, B4GALT1, LY6G5B, FADS1, COX4I1, MGP, SMAD1, CDO1, SOD2, MAPK14, GRM6, SERPIND1, SYT17, PTAFR, F2R	273	914	14116	1.47	1.00	0.8	58.38
GOTERM_BP_ALL	GO:0042633~hair cycle	4	1.18	0.05	KRT27, LAMA5, FST, TGFB2	273	43	14116	4.81	1.00	0.8	58.39
GOTERM_BP_ALL	GO:0042303~molting cycle	4	1.18	0.05	KRT27, LAMA5, FST, TGFB2	273	43	14116	4.81	1.00	0.8	58.39

GOTERM_BP_ALL	GO:0045667~regulation of osteoblast differentiation	4	1.18	0.05	ESRRA, SMAD5, SKI, SMAD1	273	43	14116	4.81	1.00	0.8	58.39

DAVID Cellular Component

Category	Term	Count	%	PValue	Genes	List Total	Pop Hits	Pop Total	Fold Enrich	Bonferroni	Benjamini	FDR
GOTERM_CC_FAT	GO:0005829~cytosol	46	13.53	0.00	BCAT1, ALAD, GGCT, TNFRSF25, ABHD5, KCNIP3, RPS3, CEP70, ZFP91, RPS26, BAG1, SLMAP, HNRNPD, GUCY1A3, EIF3I, NOS2, RPIA, PSMD6, PLCB1, GCHFR, GPD1, PPAP2C, NCALD, LY6G5B, SMAD5, PRKAB2, SMAD1, CDO1, CDC27, TAB2, RPL29, RPS18, UACA, CSNK1E, SERPINB8, MAPK14, RPS14, MTR, FARSB, RPS12, CPNE3, DPYD, GK, SPG11, KPNA1, CALM1	260	1330	12782	1.7	0.11	0.11	0.52
GOTERM_CC_FAT	GO:0022627~cytosolic small ribosomal subunit	5	1.47	0.01	RPS26, RPS18, RPS14, RPS12, RPS3	260	40	12782	6.15	0.93	0.74	10.83
GOTERM_CC_FAT	GO:0015935~small ribosomal subunit	6	1.76	0.01	RPS26, RPS18, RPS14, MRPS24, RPS12, RPS3	260	63	12782	4.68	0.94	0.6	11.3
GOTERM_CC_FAT	GO:0044445~cytosolic part	9	2.65	0.01	RPS26, RPS18, UACA, RPS14, SLMAP, RPS12, GUCY1A3, RPS3, RPL29	260	152	12782	2.91	0.98	0.62	15.27
GOTERM_CC_FAT	GO:0022626~cytosolic ribosome	6	1.76	0.02	RPS26, RPS18, RPS14, RPS12, RPS3, RPL29	260	81	12782	3.64	1.0	0.78	28.06
GOTERM_CC_FAT	GO:0005741~mitochondrial outer membrane	6	1.76	0.04	TSPO, TOMM22, GK, PPP1CC, VAMP1, ABCB6	260	90	12782	3.28	1.1	0.85	38.85
GOTERM_CC_FAT	GO:0043292~contractile fiber	7	2.06	0.04	MURC, SVIL, DMD, ITGB1BP2, MYOZ2, TRIM63, TPM3	260	121	12782	2.84	1.2	0.81	39.32
GOTERM_CC_FAT	GO:0005605~basal lamina	3	0.88	0.05	LAMA2, ANG, LAMA5	260	17	12782	8.68	1.3	0.84	46.56
GOTERM_CC_FAT	GO:0033279~ribosomal subunit	7	2.06	0.05	RPS26, RPS18, RPS14, MRPS24, RPS12, RPS3, RPL29	260	128	12782	2.69	1.4	0.8	46.83
GOTERM_CC_FAT	GO:0031967~organelle envelope	20	5.88	0.05	SLC2A9, TSPO, PHB, COX4I1, IPO9, SFXN2, SMAD1, PPP1CC, ABCB6, SOD2, TMEM38B, UACA, FAM156A, SLC25A28, TOMM22, GK, VAMP1, BDH1, KPNA1, GCHFR	260	620	12782	1.59	1.5	0.78	48.19
GOTERM_CC_FAT	GO:0031975~envelope	20	5.88	0.05	SLC2A9, TSPO, PHB, COX4I1, IPO9, SFXN2, SMAD1, PPP1CC, ABCB6, SOD2, TMEM38B, UACA, FAM156A, SLC25A28, TOMM22, GK, VAMP1, BDH1, KPNA1, GCHFR	260	622	12782	1.58	1.0	0.76	49.5

DAVID Molecular Functions

Category	Term	Count	%	PValue	Genes	List Total	Pop Hits	Pop Total	Fold Enrich	Bonferroni	Benjamini	FDR
GOTERM_MF_FAT	GO:0030145~manganese ion binding	9	2.65	0.01	B4GALT1, ILKAP, B4GALT3, ARG2, SMG1, PPP1CC, B4GALT7, GALNT12, SOD2	250	154	12983	3.03	0.99	0.99	13.2
GOTERM_MF_FAT	GO:0015295~solute:hydrogen symporter activity	4	1.18	0.01	SLC2A9, SLC17A5, SLC2A5, SLC15A4	250	25	12983	8.31	1,00	0.96	15.77
GOTERM_MF_FAT	GO:0005267~potassium channel activity	8	2.35	0.01	KCNH1, KCNS1, KCNK12, KCNQ1, KCTD7, KCNMB2, KCNIP3, TMEM38B	250	133	12983	3.12	1,00	0.92	18.73
GOTERM_MF_FAT	GO:0003735~structural constituent of ribosome	9	2.65	0.02	RSL1D1, RPS26, RPS18, RPS14, MRPS24, RPS12, MRPL48, RPS3, RPL29	250	168	12983	2.78	1,00	0.88	20.61
GOTERM_MF_FAT	GO:0005251~delayed rectifier potassium channel activity	3	0.88	0.02	KCNH1, KCNS1, KCNQ1	250	12	12983	12.98	1,00	0.9	26.87
GOTERM_MF_FAT	GO:0019899~enzyme binding	18	5.29	0.02	DENND5A, PRKAB2, IPO9, SKI, ABCA1, MYOZ2, SMAD1, PPP1CC, RPS3, CUL3, MLL5, PAK3, DMD, ARG2, SYTL2, PLCB1, GCHFR, RHOBTB3	250	523	12983	1.79	1,00	0.88	29.59
GOTERM_MF_FAT	GO:0008378~galactosyltransferase activity	4	1.18	0.03	B4GALT1, B4GALT3, B3GNT5, B4GALT7	250	34	12983	6.11	1,00	0.88	32.75
GOTERM_MF_FAT	GO:0005351~sugar:hydrogen symporter activity	3	0.88	0.04	SLC2A9, SLC17A5, SLC2A5	250	16	12983	9.74	1,00	0.92	42.04
GOTERM_MF_FAT	GO:0005402~cation:sugar symporter activity	3	0.88	0.04	SLC2A9, SLC17A5, SLC2A5	250	16	12983	9.74	1,00	0.92	42.04
GOTERM_MF_FAT	GO:0031267~small GTPase binding	6	1.76	0.04	DENND5A, PAK3, SYTL2, IPO9, ABCA1, RHOBTB3	250	101	12983	3.09	1,00	0.93	48.63
