# Supplementary material for: Characterisation of equine satellite cell transcriptomic profile response to β-hydroxy-β-methylbutyrate (HMB)
Source: Br J Nutr. 2016 Oct 3;116(8):1315–25. doi: 10.1017/S000711451600324X (PMC5082287; doi:10.1017/S000711451600324X)
Supplement: Supplementary file 1 [file S000711451600324Xsup.zip › S000711451600324Xsup001/S000711451600324Xsup003.pdf]

Supplementary material 3

Trageted cell processes

RelationSymbolicName	ObjectType	Effect	RelationNumberOfReferences
**Cell proliferation**			
Regulation: MAPK14 ---> cell proliferation	Regulation		540
positive Regulation: MAPK14 --+> cell proliferation	Regulation	positive	324
positive Regulation: SPP1 --+> cell proliferation	Regulation	positive	269
Regulation: TGFB2 ---> cell proliferation	Regulation		252
positive Regulation: GLI1 --+> cell proliferation	Regulation	positive	176
Regulation: NOS2 ---> cell proliferation	Regulation		158
positive Regulation: F2R --+> cell proliferation	Regulation	positive	142
negative Regulation: SOD2 ---| cell proliferation	Regulation	negative	98
positive Regulation: ANG --+> cell proliferation	Regulation	positive	79
Regulation: FST ---> cell proliferation	Regulation		75
negative Regulation: PHB ---| cell proliferation	Regulation	negative	70
Regulation: GNL3 ---> cell proliferation	Regulation		55
Regulation: SMAD1 ---> cell proliferation	Regulation		48
positive Regulation: KCNH1 --+> cell proliferation	Regulation	positive	47
Regulation: TSPO ---> cell proliferation	Regulation		46
positive Regulation: ESRRA --+> cell proliferation	Regulation	positive	41
positive Regulation: SMAD5 --+> cell proliferation	Regulation	positive	38
positive Regulation: PROK1 --+> cell proliferation	Regulation	positive	37
Regulation: CHKA ---> cell proliferation	Regulation		36
Regulation: BAG1 ---> cell proliferation	Regulation		35
Regulation: MYF5 ---> cell proliferation	Regulation		33
Regulation: S1PR2 ---> cell proliferation	Regulation		32
positive Regulation: SEMA4D --+> cell proliferation	Regulation	positive	28
negative Regulation: CCNG1 ---| cell proliferation	Regulation	negative	27
positive Regulation: LPAR2 --+> cell proliferation	Regulation	positive	25
positive Regulation: GNA12 --+> cell proliferation	Regulation	positive	24
positive Regulation: MEF2C --+> cell proliferation	Regulation	positive	24
negative Regulation: ABCA1 ---| cell proliferation	Regulation	negative	23

<table>
<tr><td>positive Regulation: GNAI1 --+> cell proliferation</td><td>Regulation</td><td>positive</td><td>22</td></tr>
<tr><td>Regulation: MEF2A ---> cell proliferation</td><td>Regulation</td><td></td><td>21</td></tr>
<tr><td>Regulation: SMARCA2 ---> cell proliferation</td><td>Regulation</td><td></td><td>21</td></tr>
<tr><td>positive Regulation: PTAFR --+> cell proliferation</td><td>Regulation</td><td>positive</td><td>21</td></tr>
<tr><td>positive Regulation: SAA1 --+> cell proliferation</td><td>Regulation</td><td>positive</td><td>20</td></tr>
<tr><td>Regulation: REST ---> cell proliferation</td><td>Regulation</td><td></td><td>19</td></tr>
<tr><td>Regulation: PRKG1 ---> cell proliferation</td><td>Regulation</td><td></td><td>19</td></tr>
<tr><td>positive Regulation: GFER --+> cell proliferation</td><td>Regulation</td><td>positive</td><td>18</td></tr>
<tr><td>positive Regulation: CHRM3 --+> cell proliferation</td><td>Regulation</td><td>positive</td><td>18</td></tr>
<tr><td>positive Regulation: MCAM --+> cell proliferation</td><td>Regulation</td><td>positive</td><td>15</td></tr>
<tr><td>positive Regulation: ARG2 --+> cell proliferation</td><td>Regulation</td><td>positive</td><td>13</td></tr>
<tr><td>positive Regulation: TPD52 --+> cell proliferation</td><td>Regulation</td><td>positive</td><td>12</td></tr>
<tr><td>negative Regulation: KLF11 ---| cell proliferation</td><td>Regulation</td><td>negative</td><td>11</td></tr>
<tr><td>negative Regulation: PLCB1 ---| cell proliferation</td><td>Regulation</td><td>negative</td><td>11</td></tr>
<tr><td>Regulation: GRB10 ---> cell proliferation</td><td>Regulation</td><td></td><td>11</td></tr>
<tr><td>Regulation: PRKAA1 ---> cell proliferation</td><td>Regulation</td><td></td><td>11</td></tr>
<tr><td>negative Regulation: NOS2 ---| cell proliferation</td><td>Regulation</td><td>negative</td><td>10</td></tr>
<tr><td>positive Regulation: HOXB9 --+> cell proliferation</td><td>Regulation</td><td>positive</td><td>10</td></tr>
<tr><td>negative Regulation: HOXB13 ---| cell proliferation</td><td>Regulation</td><td>negative</td><td>10</td></tr>
<tr><td>Regulation: SKI ---> cell proliferation</td><td>Regulation</td><td></td><td>10</td></tr>
<tr><td>Regulation: PAK3 ---> cell proliferation</td><td>Regulation</td><td></td><td>9</td></tr>
<tr><td>Regulation: ALKBH3 ---> cell proliferation</td><td>Regulation</td><td></td><td>9</td></tr>
<tr><td>negative Regulation: TP53BP1 ---| cell proliferation</td><td>Regulation</td><td>negative</td><td>9</td></tr>
<tr><td>negative Regulation: MAP1LC3B ---| cell proliferation</td><td>Regulation</td><td>negative</td><td>9</td></tr>
<tr><td>Regulation: NFATC3 ---> cell proliferation</td><td>Regulation</td><td></td><td>9</td></tr>
<tr><td>negative Regulation: RPS14 ---| cell proliferation</td><td>Regulation</td><td>negative</td><td>9</td></tr>
<tr><td>negative Regulation: FOSL2 ---| cell proliferation</td><td>Regulation</td><td>negative</td><td>9</td></tr>
<tr><td>Regulation: HNRNPD ---> cell proliferation</td><td>Regulation</td><td></td><td>9</td></tr>
<tr><td>Regulation: KCNQ1 ---> cell proliferation</td><td>Regulation</td><td></td><td>8</td></tr>
<tr><td>positive Regulation: BCAT1 --+> cell proliferation</td><td>Regulation</td><td>positive</td><td>7</td></tr>
<tr><td>Regulation: TNFRSF25 ---> cell proliferation</td><td>Regulation</td><td></td><td>7</td></tr>
<tr><td>positive Regulation: CHD7 --+> cell proliferation</td><td>Regulation</td><td>positive</td><td>7</td></tr>
<tr><td>positive Regulation: EIF3I --+> cell proliferation</td><td>Regulation</td><td>positive</td><td>7</td></tr>
<tr><td>positive Regulation: TLR1 --+> cell proliferation</td><td>Regulation</td><td>positive</td><td>7</td></tr>
<tr><td>negative Regulation: TMSB10 ---| cell proliferation</td><td>Regulation</td><td>negative</td><td>6</td></tr>
</table>

positive Regulation: EDIL3 --+> cell proliferation	Regulation	positive	6
Regulation: CUL3 ---> cell proliferation	Regulation		6
positive Regulation: CPE --+> cell proliferation	Regulation	positive	6
negative Regulation: MAL ---| cell proliferation	Regulation	negative	5
Regulation: SMG1 ---> cell proliferation	Regulation		5
Regulation: STK39 ---> cell proliferation	Regulation		5
negative Regulation: NPRL2 ---| cell proliferation	Regulation	negative	5
positive Regulation: ABCB6 --+> cell proliferation	Regulation	positive	5
Regulation: PEG3 ---> cell proliferation	Regulation		5
positive Regulation: B4GALT1 --+> cell proliferation	Regulation	positive	5
Regulation: PPARGC1B ---> cell proliferation	Regulation		5
positive Regulation: RPL29 --+> cell proliferation	Regulation	positive	5
positive Regulation: CSNK1E --+> cell proliferation	Regulation	positive	4
Regulation: FOXJ1 ---> cell proliferation	Regulation		4
positive Regulation: NUDT1 --+> cell proliferation	Regulation	positive	4
positive Regulation: VAMP8 --+> cell proliferation	Regulation	positive	4
negative Regulation: ABI3BP ---| cell proliferation	Regulation	negative	4
Regulation: LAMA5 ---> cell proliferation	Regulation		4
positive Regulation: JADE1 --+> cell proliferation	Regulation	positive	4
positive Regulation: RSL1D1 --+> cell proliferation	Regulation	positive	4
Regulation: MGP ---> cell proliferation	Regulation		4
Regulation: PMAIP1 ---> cell proliferation	Regulation		4
positive Regulation: GGCT --+> cell proliferation	Regulation	positive	3
Regulation: TPD52L1 ---> cell proliferation	Regulation		3
positive Regulation: PLXDC2 --+> cell proliferation	Regulation	positive	3
Regulation: KIF2A ---> cell proliferation	Regulation		3
Regulation: SLC2A5 ---> cell proliferation	Regulation		3
Regulation: KCNIP3 ---> cell proliferation	Regulation		3
positive Regulation: TRAPPC4 --+> cell proliferation	Regulation	positive	3
positive Regulation: MAFK --+> cell proliferation	Regulation	positive	3
Regulation: UBE2V1 ---> cell proliferation	Regulation		3
Regulation: CALM1 ---> cell proliferation	Regulation		3
negative Regulation: FAM107A ---| cell proliferation	Regulation	negative	3
negative Regulation: BEX1 ---| cell proliferation	Regulation	negative	3
Regulation: GUCY1A3 ---> cell proliferation	Regulation		3

<table>
<tr><td>positive Regulation: IL22RA1 --+> cell proliferation</td><td>Regulation</td><td>positive</td><td>3</td></tr>
<tr><td>Regulation: SERPIND1 ---> cell proliferation</td><td>Regulation</td><td></td><td>3</td></tr>
<tr><td>Regulation: RBM17 ---> cell proliferation</td><td>Regulation</td><td></td><td>3</td></tr>
<tr><td>Regulation: DMD ---> cell proliferation</td><td>Regulation</td><td></td><td>3</td></tr>
<tr><td>positive Regulation: CAPN6 --+> cell proliferation</td><td>Regulation</td><td>positive</td><td>3</td></tr>
<tr><td>negative Regulation: ILKAP ---| cell proliferation</td><td>Regulation</td><td>negative</td><td>3</td></tr>
<tr><td>Regulation: EML4 ---> cell proliferation</td><td>Regulation</td><td></td><td>3</td></tr>
<tr><td>Regulation: KMT2E ---> cell proliferation</td><td>Regulation</td><td></td><td>2</td></tr>
<tr><td>negative Regulation: ZFP36L2 ---| cell proliferation</td><td>Regulation</td><td>negative</td><td>2</td></tr>
<tr><td>negative Regulation: LAMA2 ---| cell proliferation</td><td>Regulation</td><td>negative</td><td>2</td></tr>
<tr><td>Regulation: F2RL2 ---> cell proliferation</td><td>Regulation</td><td></td><td>2</td></tr>
<tr><td>Regulation: MACF1 ---> cell proliferation</td><td>Regulation</td><td></td><td>2</td></tr>
<tr><td>Regulation: SLCO2A1 ---> cell proliferation</td><td>Regulation</td><td></td><td>2</td></tr>
<tr><td>Regulation: TRIM37 ---> cell proliferation</td><td>Regulation</td><td></td><td>2</td></tr>
<tr><td>positive Regulation: BRF2 --+> cell proliferation</td><td>Regulation</td><td>positive</td><td>2</td></tr>
<tr><td>Regulation: MLX ---> cell proliferation</td><td>Regulation</td><td></td><td>2</td></tr>
<tr><td>Regulation: GDF10 ---> cell proliferation</td><td>Regulation</td><td></td><td>2</td></tr>
<tr><td>negative Regulation: HN1 ---| cell proliferation</td><td>Regulation</td><td>negative</td><td>2</td></tr>
<tr><td>positive Regulation: NKX2-2 --+> cell proliferation</td><td>Regulation</td><td>positive</td><td>2</td></tr>
<tr><td>Regulation: HMCN1 ---> cell proliferation</td><td>Regulation</td><td></td><td>2</td></tr>
<tr><td>Regulation: RPS3 ---> cell proliferation</td><td>Regulation</td><td></td><td>2</td></tr>
<tr><td>positive Regulation: SLC31A1 --+> cell proliferation</td><td>Regulation</td><td>positive</td><td>2</td></tr>
<tr><td>negative Regulation: KPNA1 ---| cell proliferation</td><td>Regulation</td><td>negative</td><td>2</td></tr>
<tr><td>Regulation: BICC1 ---> cell proliferation</td><td>Regulation</td><td></td><td>2</td></tr>
<tr><td>negative Regulation: RBMS3 ---| cell proliferation</td><td>Regulation</td><td>negative</td><td>2</td></tr>
<tr><td>Regulation: STK17B ---> cell proliferation</td><td>Regulation</td><td></td><td>2</td></tr>
<tr><td>Regulation: ZBTB18 ---> cell proliferation</td><td>Regulation</td><td></td><td>2</td></tr>
</table>

Apoptosis

<table>
<tr><td>positive Regulation: MAPK14 --+> apoptosis</td><td>Regulation</td><td>positive</td><td>743</td></tr>
<tr><td>Regulation: NOS2 ---> apoptosis</td><td>Regulation</td><td></td><td>652</td></tr>
<tr><td>negative Regulation: SOD2 ---| apoptosis</td><td>Regulation</td><td>negative</td><td>353</td></tr>
<tr><td>positive Regulation: PMAIP1 --+> apoptosis</td><td>Regulation</td><td>positive</td><td>257</td></tr>
<tr><td>Regulation: SPP1 ---> apoptosis</td><td>Regulation</td><td></td><td>203</td></tr>
<tr><td>negative Regulation: BAG1 ---| apoptosis</td><td>Regulation</td><td>negative</td><td>172</td></tr>
</table>

Regulation: TSPO ---> apoptosis	Regulation		94
Regulation: TGFB2 ---> apoptosis	Regulation		91
Regulation: PHB ---> apoptosis	Regulation		83
Regulation: F2R ---> apoptosis	Regulation		77
negative Regulation: GLI1 ---| apoptosis	Regulation	negative	59
positive Regulation: TNFRSF25 --+> apoptosis	Regulation	positive	51
Regulation: MEF2A ---> apoptosis	Regulation		42
Regulation: PTAFR ---> apoptosis	Regulation		40
positive Regulation: STK17B --+> apoptosis	Regulation	positive	32
positive Regulation: CCNG1 --+> apoptosis	Regulation	positive	31
Regulation: ABCA1 ---> apoptosis	Regulation		28
Regulation: MAP1LC3B ---> apoptosis	Regulation		28
Regulation: FST ---> apoptosis	Regulation		27
positive Regulation: KCNIP3 --+> apoptosis	Regulation	positive	26
Regulation: ANG ---> apoptosis	Regulation		24
negative Regulation: MEF2C ---| apoptosis	Regulation	negative	21
Regulation: SAA1 ---> apoptosis	Regulation		21
Regulation: RPS3 ---> apoptosis	Regulation		21
positive Regulation: TP53BP1 --+> apoptosis	Regulation	positive	21
positive Regulation: IP6K2 --+> apoptosis	Regulation	positive	20
negative Regulation: GFER ---| apoptosis	Regulation	negative	19
negative Regulation: REST ---| apoptosis	Regulation	negative	18
positive Regulation: PEG3 --+> apoptosis	Regulation	positive	18
Regulation: RPS14 ---> apoptosis	Regulation		18
positive Regulation: KLF11 --+> apoptosis	Regulation	positive	17
positive Regulation: GNA12 --+> apoptosis	Regulation	positive	16
Regulation: EDIL3 ---> apoptosis	Regulation		15
negative Regulation: SMAD5 ---| apoptosis	Regulation	negative	15
Regulation: TMSB10 ---> apoptosis	Regulation		15
positive Regulation: S1PR2 --+> apoptosis	Regulation	positive	15
Regulation: GRB10 ---> apoptosis	Regulation		15
negative Regulation: PRKAA1 ---| apoptosis	Regulation	negative	14
negative Regulation: LPAR2 ---| apoptosis	Regulation	negative	14
negative Regulation: SMAD1 ---| apoptosis	Regulation	negative	14
negative Regulation: GNL3 ---| apoptosis	Regulation	negative	14

<table>
<tr><td>positive Regulation: SEMA4D --+> apoptosis</td><td>Regulation</td><td>positive</td><td>13</td></tr>
<tr><td>Regulation: ARG2 ---> apoptosis</td><td>Regulation</td><td></td><td>12</td></tr>
<tr><td>negative Regulation: ESRRA ---| apoptosis</td><td>Regulation</td><td>negative</td><td>12</td></tr>
<tr><td>Regulation: TRIM63 ---> apoptosis</td><td>Regulation</td><td></td><td>11</td></tr>
<tr><td>negative Regulation: CHRM3 ---| apoptosis</td><td>Regulation</td><td>negative</td><td>11</td></tr>
<tr><td>negative Regulation: PAK3 ---| apoptosis</td><td>Regulation</td><td>negative</td><td>11</td></tr>
<tr><td>Regulation: STEAP3 ---> apoptosis</td><td>Regulation</td><td></td><td>11</td></tr>
<tr><td>negative Regulation: CHKA ---| apoptosis</td><td>Regulation</td><td>negative</td><td>10</td></tr>
<tr><td>positive Regulation: B4GALT1 --+> apoptosis</td><td>Regulation</td><td>positive</td><td>9</td></tr>
<tr><td>Regulation: PRKG1 ---> apoptosis</td><td>Regulation</td><td></td><td>9</td></tr>
<tr><td>negative Regulation: MCAM ---| apoptosis</td><td>Regulation</td><td>negative</td><td>8</td></tr>
<tr><td>positive Regulation: NOS2 --+> apoptosis</td><td>Regulation</td><td>positive</td><td>8</td></tr>
<tr><td>positive Regulation: ABCC8 --+> apoptosis</td><td>Regulation</td><td>positive</td><td>8</td></tr>
<tr><td>negative Regulation: LAMP2 ---| apoptosis</td><td>Regulation</td><td>negative</td><td>8</td></tr>
<tr><td>negative Regulation: SMG1 ---| apoptosis</td><td>Regulation</td><td>negative</td><td>8</td></tr>
<tr><td>negative Regulation: CA4 ---| apoptosis</td><td>Regulation</td><td>negative</td><td>8</td></tr>
<tr><td>positive Regulation: NFATC3 --+> apoptosis</td><td>Regulation</td><td>positive</td><td>8</td></tr>
<tr><td>negative Regulation: CAPN6 ---| apoptosis</td><td>Regulation</td><td>negative</td><td>8</td></tr>
<tr><td>positive Regulation: NPRL2 --+> apoptosis</td><td>Regulation</td><td>positive</td><td>8</td></tr>
<tr><td>Regulation: CPE ---> apoptosis</td><td>Regulation</td><td></td><td>7</td></tr>
<tr><td>Regulation: FOSL2 ---> apoptosis</td><td>Regulation</td><td></td><td>7</td></tr>
<tr><td>positive Regulation: TLR1 --+> apoptosis</td><td>Regulation</td><td>positive</td><td>7</td></tr>
<tr><td>Regulation: TPD52 ---> apoptosis</td><td>Regulation</td><td></td><td>7</td></tr>
<tr><td>positive Regulation: JADE1 --+> apoptosis</td><td>Regulation</td><td>positive</td><td>7</td></tr>
<tr><td>positive Regulation: UACA --+> apoptosis</td><td>Regulation</td><td>positive</td><td>7</td></tr>
<tr><td>positive Regulation: BEX1 --+> apoptosis</td><td>Regulation</td><td>positive</td><td>6</td></tr>
<tr><td>negative Regulation: ITGB1BP2 ---| apoptosis</td><td>Regulation</td><td>negative</td><td>6</td></tr>
<tr><td>Regulation: DMD ---> apoptosis</td><td>Regulation</td><td></td><td>6</td></tr>
<tr><td>Regulation: SMARCA2 ---> apoptosis</td><td>Regulation</td><td></td><td>6</td></tr>
<tr><td>negative Regulation: PROK1 ---| apoptosis</td><td>Regulation</td><td>negative</td><td>6</td></tr>
<tr><td>positive Regulation: PPP1CC --+> apoptosis</td><td>Regulation</td><td>positive</td><td>5</td></tr>
<tr><td>negative Regulation: GHITM ---| apoptosis</td><td>Regulation</td><td>negative</td><td>5</td></tr>
<tr><td>negative Regulation: ALKBH3 ---| apoptosis</td><td>Regulation</td><td>negative</td><td>5</td></tr>
<tr><td>positive Regulation: STK39 --+> apoptosis</td><td>Regulation</td><td>positive</td><td>5</td></tr>
<tr><td>positive Regulation: MGP --+> apoptosis</td><td>Regulation</td><td>positive</td><td>5</td></tr>
</table>

<table>
<tr><td>Regulation: TOMM22 ---> apoptosis</td><td>Regulation</td><td></td><td>5</td></tr>
<tr><td>Regulation: DPF2 ---> apoptosis</td><td>Regulation</td><td></td><td>4</td></tr>
<tr><td>positive Regulation: HOXB13 --+> apoptosis</td><td>Regulation</td><td>positive</td><td>4</td></tr>
<tr><td>Regulation: PLCB1 ---> apoptosis</td><td>Regulation</td><td></td><td>4</td></tr>
<tr><td>Regulation: MMP15 ---> apoptosis</td><td>Regulation</td><td></td><td>4</td></tr>
<tr><td>positive Regulation: MAL --+> apoptosis</td><td>Regulation</td><td>positive</td><td>4</td></tr>
<tr><td>negative Regulation: GNAI1 ---| apoptosis</td><td>Regulation</td><td>negative</td><td>4</td></tr>
<tr><td>positive Regulation: HNRNPD --+> apoptosis</td><td>Regulation</td><td>positive</td><td>4</td></tr>
<tr><td>negative Regulation: PPARGC1B ---| apoptosis</td><td>Regulation</td><td>negative</td><td>3</td></tr>
<tr><td>Regulation: NSMAF ---> apoptosis</td><td>Regulation</td><td></td><td>3</td></tr>
<tr><td>positive Regulation: FAM107A --+> apoptosis</td><td>Regulation</td><td>positive</td><td>3</td></tr>
<tr><td>Regulation: CSNK1E ---> apoptosis</td><td>Regulation</td><td></td><td>3</td></tr>
<tr><td>Regulation: UBE2V1 ---> apoptosis</td><td>Regulation</td><td></td><td>3</td></tr>
<tr><td>negative Regulation: KCNH1 ---| apoptosis</td><td>Regulation</td><td>negative</td><td>3</td></tr>
<tr><td>Regulation: GK ---> apoptosis</td><td>Regulation</td><td></td><td>3</td></tr>
<tr><td>Regulation: LAMA2 ---> apoptosis</td><td>Regulation</td><td></td><td>3</td></tr>
<tr><td>Regulation: CDC27 ---> apoptosis</td><td>Regulation</td><td></td><td>3</td></tr>
<tr><td>Regulation: RPL29 ---> apoptosis</td><td>Regulation</td><td></td><td>3</td></tr>
<tr><td>Regulation: BCAT1 ---> apoptosis</td><td>Regulation</td><td></td><td>3</td></tr>
<tr><td>Regulation: DPYD ---> apoptosis</td><td>Regulation</td><td></td><td>3</td></tr>
<tr><td>positive Regulation: ILKAP --+> apoptosis</td><td>Regulation</td><td>positive</td><td>3</td></tr>
<tr><td>Regulation: CALM1 ---> apoptosis</td><td>Regulation</td><td></td><td>2</td></tr>
<tr><td>Regulation: CUL3 ---> apoptosis</td><td>Regulation</td><td></td><td>2</td></tr>
<tr><td>Regulation: GGCT ---> apoptosis</td><td>Regulation</td><td></td><td>2</td></tr>
<tr><td>Regulation: TAB2 ---> apoptosis</td><td>Regulation</td><td></td><td>2</td></tr>
<tr><td>negative Regulation: PRPS2 ---| apoptosis</td><td>Regulation</td><td>negative</td><td>2</td></tr>
<tr><td>Regulation: RBM17 ---> apoptosis</td><td>Regulation</td><td></td><td>2</td></tr>
<tr><td>Regulation: SYPL2 ---> apoptosis</td><td>Regulation</td><td></td><td>2</td></tr>
<tr><td>positive Regulation: RSL1D1 --+> apoptosis</td><td>Regulation</td><td>positive</td><td>2</td></tr>
<tr><td>Regulation: ABHD5 ---> apoptosis</td><td>Regulation</td><td></td><td>2</td></tr>
<tr><td>Regulation: ABCB6 ---> apoptosis</td><td>Regulation</td><td></td><td>2</td></tr>
<tr><td>Regulation: LAMA5 ---> apoptosis</td><td>Regulation</td><td></td><td>2</td></tr>
<tr><td>Regulation: ZFP36L2 ---> apoptosis</td><td>Regulation</td><td></td><td>2</td></tr>
<tr><td>positive Regulation: SLC31A1 --+> apoptosis</td><td>Regulation</td><td>positive</td><td>2</td></tr>
<tr><td>Regulation: KCNQ1 ---> apoptosis</td><td>Regulation</td><td></td><td>2</td></tr>
</table>

Cell differentiation

<table>
<tr><td>Regulation: MAPK14 ---> cell differentiation</td><td>Regulation</td><td></td><td>452</td></tr>
<tr><td>Regulation: SPP1 ---> cell differentiation</td><td>Regulation</td><td></td><td>138</td></tr>
<tr><td>Regulation: TGFB2 ---> cell differentiation</td><td>Regulation</td><td></td><td>125</td></tr>
<tr><td>positive Regulation: MEF2A --+> cell differentiation</td><td>Regulation</td><td>positive</td><td>119</td></tr>
<tr><td>Regulation: MYF5 ---> cell differentiation</td><td>Regulation</td><td></td><td>109</td></tr>
<tr><td>positive Regulation: TGFB2 --+> cell differentiation</td><td>Regulation</td><td>positive</td><td>105</td></tr>
<tr><td>Regulation: NKX2-2 ---> cell differentiation</td><td>Regulation</td><td></td><td>98</td></tr>
<tr><td>Regulation: SMAD1 ---> cell differentiation</td><td>Regulation</td><td></td><td>86</td></tr>
<tr><td>Regulation: MEF2C ---> cell differentiation</td><td>Regulation</td><td></td><td>84</td></tr>
<tr><td>Regulation: FST ---> cell differentiation</td><td>Regulation</td><td></td><td>70</td></tr>
<tr><td>negative Regulation: REST ---| cell differentiation</td><td>Regulation</td><td>negative</td><td>61</td></tr>
<tr><td>Regulation: GLI1 ---> cell differentiation</td><td>Regulation</td><td></td><td>50</td></tr>
<tr><td>positive Regulation: SMAD5 --+> cell differentiation</td><td>Regulation</td><td>positive</td><td>46</td></tr>
<tr><td>Regulation: NOS2 ---> cell differentiation</td><td>Regulation</td><td></td><td>44</td></tr>
<tr><td>positive Regulation: SOD2 --+> cell differentiation</td><td>Regulation</td><td>positive</td><td>37</td></tr>
<tr><td>Regulation: PLCB1 ---> cell differentiation</td><td>Regulation</td><td></td><td>35</td></tr>
<tr><td>positive Regulation: ESRRA --+> cell differentiation</td><td>Regulation</td><td>positive</td><td>31</td></tr>
<tr><td>negative Regulation: MGP ---| cell differentiation</td><td>Regulation</td><td>negative</td><td>28</td></tr>
<tr><td>positive Regulation: FOXJ1 --+> cell differentiation</td><td>Regulation</td><td>positive</td><td>24</td></tr>
<tr><td>positive Regulation: F2R --+> cell differentiation</td><td>Regulation</td><td>positive</td><td>23</td></tr>
<tr><td>Regulation: SEMA4D ---> cell differentiation</td><td>Regulation</td><td></td><td>23</td></tr>
<tr><td>positive Regulation: SAA1 --+> cell differentiation</td><td>Regulation</td><td>positive</td><td>22</td></tr>
<tr><td>Regulation: PHB ---> cell differentiation</td><td>Regulation</td><td></td><td>16</td></tr>
<tr><td>positive Regulation: BAG1 --+> cell differentiation</td><td>Regulation</td><td>positive</td><td>16</td></tr>
<tr><td>Regulation: S1PR2 ---> cell differentiation</td><td>Regulation</td><td></td><td>15</td></tr>
<tr><td>positive Regulation: NFATC3 --+> cell differentiation</td><td>Regulation</td><td>positive</td><td>14</td></tr>
<tr><td>positive Regulation: SMARCA2 --+> cell differentiation</td><td>Regulation</td><td>positive</td><td>14</td></tr>
<tr><td>negative Regulation: CHKA ---| cell differentiation</td><td>Regulation</td><td>negative</td><td>12</td></tr>
<tr><td>positive Regulation: PROK1 --+> cell differentiation</td><td>Regulation</td><td>positive</td><td>12</td></tr>
<tr><td>positive Regulation: HOXB13 --+> cell differentiation</td><td>Regulation</td><td>positive</td><td>12</td></tr>
<tr><td>positive Regulation: TSPO --+> cell differentiation</td><td>Regulation</td><td>positive</td><td>12</td></tr>
<tr><td>Regulation: SPON1 ---> cell differentiation</td><td>Regulation</td><td></td><td>11</td></tr>
<tr><td>positive Regulation: MAFK --+> cell differentiation</td><td>Regulation</td><td>positive</td><td>11</td></tr>
</table>

Regulation: GNL3 ---> cell differentiation	Regulation		11
Regulation: RPS14 ---> cell differentiation	Regulation		10
positive Regulation: GLI1 --+> cell differentiation	Regulation	positive	10
Regulation: PRKG1 ---> cell differentiation	Regulation		10
Regulation: GPD1 ---> cell differentiation	Regulation		10
Regulation: LAMA5 ---> cell differentiation	Regulation		9
Regulation: DMD ---> cell differentiation	Regulation		9
positive Regulation: SKI --+> cell differentiation	Regulation	positive	9
positive Regulation: KMT2E --+> cell differentiation	Regulation	positive	8
Regulation: GNA12 ---> cell differentiation	Regulation		8
positive Regulation: GDF10 --+> cell differentiation	Regulation	positive	8
positive Regulation: LAMA2 --+> cell differentiation	Regulation	positive	7
Regulation: FOSL2 ---> cell differentiation	Regulation		7
positive Regulation: HOXB9 --+> cell differentiation	Regulation	positive	7
Regulation: PPARGC1B ---> cell differentiation	Regulation		6
positive Regulation: PAK3 --+> cell differentiation	Regulation	positive	6
Regulation: GRB10 ---> cell differentiation	Regulation		6
positive Regulation: MCAM --+> cell differentiation	Regulation	positive	6
positive Regulation: RBFOX1 --+> cell differentiation	Regulation	positive	5
positive Regulation: MAL --+> cell differentiation	Regulation	positive	5
Regulation: GNAI1 ---> cell differentiation	Regulation		5
Regulation: KPNA1 ---> cell differentiation	Regulation		5
Regulation: PEG3 ---> cell differentiation	Regulation		5
negative Regulation: PTAFR ---| cell differentiation	Regulation	negative	5
Regulation: TBX15 ---> cell differentiation	Regulation		5
Regulation: BEX1 ---> cell differentiation	Regulation		4
Regulation: ZBTB18 ---> cell differentiation	Regulation		4
Regulation: B4GALT1 ---> cell differentiation	Regulation		4
Regulation: STK39 ---> cell differentiation	Regulation		4
Regulation: GPR126 ---> cell differentiation	Regulation		4
Regulation: CHD7 ---> cell differentiation	Regulation		4
Regulation: KLF11 ---> cell differentiation	Regulation		4
positive Regulation: LPAR2 --+> cell differentiation	Regulation	positive	4
Regulation: PRKAA1 ---> cell differentiation	Regulation		4
positive Regulation: ABCB6 --+> cell differentiation	Regulation	positive	4

<table>
<tr><td>Regulation: RPL29 ---> cell differentiation</td><td>Regulation</td><td></td><td>4</td></tr>
<tr><td>Regulation: KCNH1 ---> cell differentiation</td><td>Regulation</td><td></td><td>4</td></tr>
<tr><td>Regulation: TNFRSF25 ---> cell differentiation</td><td>Regulation</td><td></td><td>3</td></tr>
<tr><td>Regulation: EDIL3 ---> cell differentiation</td><td>Regulation</td><td></td><td>3</td></tr>
<tr><td>Regulation: HACD1 ---> cell differentiation</td><td>Regulation</td><td></td><td>3</td></tr>
<tr><td>Regulation: BCAT1 ---> cell differentiation</td><td>Regulation</td><td></td><td>3</td></tr>
<tr><td>Regulation: EIF3I ---> cell differentiation</td><td>Regulation</td><td></td><td>3</td></tr>
<tr><td>Regulation: SLC2A5 ---> cell differentiation</td><td>Regulation</td><td></td><td>3</td></tr>
<tr><td>Regulation: MLX ---> cell differentiation</td><td>Regulation</td><td></td><td>3</td></tr>
<tr><td>Regulation: CUL3 ---> cell differentiation</td><td>Regulation</td><td></td><td>3</td></tr>
<tr><td>Regulation: UBE2V1 ---> cell differentiation</td><td>Regulation</td><td></td><td>3</td></tr>
<tr><td>positive Regulation: ABCA1 --+> cell differentiation</td><td>Regulation</td><td>positive</td><td>3</td></tr>
<tr><td>Regulation: PPP1CC ---> cell differentiation</td><td>Regulation</td><td></td><td>2</td></tr>
<tr><td>Regulation: MMP15 ---> cell differentiation</td><td>Regulation</td><td></td><td>2</td></tr>
<tr><td>positive Regulation: ABI3BP --+> cell differentiation</td><td>Regulation</td><td>positive</td><td>2</td></tr>
<tr><td>Regulation: DUSP13 ---> cell differentiation</td><td>Regulation</td><td></td><td>2</td></tr>
<tr><td>Regulation: CHRM3 ---> cell differentiation</td><td>Regulation</td><td></td><td>2</td></tr>
<tr><td>positive Regulation: TRIM63 --+> cell differentiation</td><td>Regulation</td><td>positive</td><td>2</td></tr>
<tr><td>Regulation: KCNIP3 ---> cell differentiation</td><td>Regulation</td><td></td><td>2</td></tr>
<tr><td>Regulation: CSNK1E ---> cell differentiation</td><td>Regulation</td><td></td><td>2</td></tr>
<tr><td>Regulation: ANG ---> cell differentiation</td><td>Regulation</td><td></td><td>2</td></tr>
<tr><td>Regulation: TLR1 ---> cell differentiation</td><td>Regulation</td><td></td><td>2</td></tr>
<tr><td>negative Regulation: MURC ---| cell differentiation</td><td>Regulation</td><td>negative</td><td>2</td></tr>
<tr><td>positive Regulation: MAP1LC3B --+> cell differentiation</td><td>Regulation</td><td>positive</td><td>2</td></tr>
<tr><td>negative Regulation: ZFP36L2 ---| cell differentiation</td><td>Regulation</td><td>negative</td><td>2</td></tr>
<tr><td>Regulation: SMG1 ---> cell differentiation</td><td>Regulation</td><td></td><td>2</td></tr>
<tr><td>Regulation: LANCL1 ---> cell differentiation</td><td>Regulation</td><td></td><td>2</td></tr>
<tr><td>positive Regulation: SLC31A1 --+> cell differentiation</td><td>Regulation</td><td>positive</td><td>2</td></tr>
<tr><td>Regulation: MACF1 ---> cell differentiation</td><td>Regulation</td><td></td><td>2</td></tr>
<tr><td>Regulation: JADE1 ---> cell differentiation</td><td>Regulation</td><td></td><td>2</td></tr>
<tr><td>Regulation: PLXDC2 ---> cell differentiation</td><td>Regulation</td><td></td><td>2</td></tr>
</table>

Cell adhesion

positive Regulation: SPP1 --+> cell adhesion	Regulation	positive	184
positive Regulation: MAPK14 --+> cell adhesion	Regulation	positive	23

positive Regulation: MCAM --+> cell adhesion	Regulation	positive	17
positive Regulation: LAMA2 --+> cell adhesion	Regulation	positive	7
positive Regulation: TGFB2 --+> cell adhesion	Regulation	positive	6
Regulation: LAMP2 ---> cell adhesion	Regulation		5
Regulation: GNA12 ---> cell adhesion	Regulation		5
Regulation: ANG ---> cell adhesion	Regulation		4
Regulation: SPON1 ---> cell adhesion	Regulation		4
Regulation: CYTIP ---> cell adhesion	Regulation		3
negative Regulation: SAA1 ---| cell adhesion	Regulation	negative	3
Regulation: DMD ---> cell adhesion	Regulation		3
Regulation: SEMA4D ---> cell adhesion	Regulation		3
Regulation: BAG1 ---> cell adhesion	Regulation		3
Regulation: CHRM3 ---> cell adhesion	Regulation		3
Regulation: LAMA5 ---> cell adhesion	Regulation		3
Regulation: EDIL3 ---> cell adhesion	Regulation		3
positive Regulation: CSNK1E --+> cell adhesion	Regulation	positive	2
Regulation: PTAFR ---> cell adhesion	Regulation		2
Regulation: PLXDC2 ---> cell adhesion	Regulation		2
Regulation: PHB ---> cell adhesion	Regulation		2

Cytoskeleton orgnization and biogenesis

positive Regulation: MAPK14 --+> cytoskeleton organizatio	Regulation	positive	48
positive Regulation: SPP1 --+> cytoskeleton organization a	Regulation	positive	21
Regulation: F2R ---> cytoskeleton organization and biogene	Regulation		18
positive Regulation: MCAM --+> cytoskeleton organization a	Regulation	positive	6
Regulation: CAPN6 ---> cytoskeleton organization and biog	Regulation		5
Regulation: GNA12 ---> cytoskeleton organization and biog	Regulation		4
Regulation: DMD ---> cytoskeleton organization and biogen	Regulation		3
Regulation: ANG ---> cytoskeleton organization and biogen	Regulation		2
Regulation: SMAD1 ---> cytoskeleton organization and biog	Regulation		2
Regulation: PAK3 ---> cytoskeleton organization and bioge	Regulation		2
positive Regulation: RBFOX1 --+> cytoskeleton organizatio	Regulation	positive	2
Regulation: S1PR2 ---> cytoskeleton organization and biog	Regulation		2
Regulation: PRKG1 ---> cytoskeleton organization and biog	Regulation		2
Regulation: GNAI1 ---> cytoskeleton organization and bioge	Regulation		2

Regulation: LPAR2 ---> cytoskeleton organization and biog	Regulation		2
positive Regulation: SEMA4D --+> cytoskeleton organizatio	Regulation	positive	2
Regulation: TGFB2 ---> cytoskeleton organization and biog	Regulation		2
Regulation: MACF1 ---> cytoskeleton organization and biog	Regulation		2
**Protein degradation**			
Regulation: TRIM63 ---> protein degradation	Regulation		56
positive Regulation: BAG1 --+> protein degradation	Regulation	positive	11
Regulation: CUL3 ---> protein degradation	Regulation		8
positive Regulation: MAPK14 --+> protein degradation	Regulation	positive	6
negative Regulation: PPARGC1B ---| protein degradation	Regulation	negative	6
Regulation: LAMP2 ---> protein degradation	Regulation		5
Regulation: SPP1 ---> protein degradation	Regulation		4
negative Regulation: KPNA1 ---| protein degradation	Regulation	negative	3
Regulation: ABCA1 ---> protein degradation	Regulation		3
Regulation: NOS2 ---> protein degradation	Regulation		2
positive Regulation: TGFB2 --+> protein degradation	Regulation	positive	2
Regulation: PMAIP1 ---> protein degradation	Regulation		2
Regulation: HNRNPD ---> protein degradation	Regulation		2
**G1/S transition**			
positive Regulation: MAPK14 --+> G1/S transition	Regulation	positive	25
negative Regulation: PHB ---| G1/S transition	Regulation	negative	8
negative Regulation: CCNG1 ---| G1/S transition	Regulation	negative	6
negative Regulation: TGFB2 ---| G1/S transition	Regulation	negative	5
Regulation: KMT2E ---> G1/S transition	Regulation		4
positive Regulation: GLI1 --+> G1/S transition	Regulation	positive	4
Regulation: ESRRA ---> G1/S transition	Regulation		3
negative Regulation: SMARCA2 ---| G1/S transition	Regulation	negative	3
Regulation: GNL3 ---> G1/S transition	Regulation		3
Regulation: SPP1 ---> G1/S transition	Regulation		2
**Lipid metabolism**			
Regulation: ABCA1 ---> lipid metabolism	Regulation		41
Regulation: ABHD5 ---> lipid metabolism	Regulation		14

positive Regulation: PPARGC1B --+> lipid metabolism	Regulation	positive	14
Regulation: ESRRA ---> lipid metabolism	Regulation		7
negative Regulation: PHB ---| lipid metabolism	Regulation	negative	6
Regulation: KLF11 ---> lipid metabolism	Regulation		5
Regulation: GPD1 ---> lipid metabolism	Regulation		5
Regulation: SAA1 ---> lipid metabolism	Regulation		5
Regulation: MAPK14 ---> lipid metabolism	Regulation		5
Regulation: GK ---> lipid metabolism	Regulation		3
negative Regulation: NOS2 ---| lipid metabolism	Regulation	negative	3
Regulation: NPC1L1 ---> lipid metabolism	Regulation		2
Regulation: FADS1 ---> lipid metabolism	Regulation		2
Regulation: S1PR2 ---> lipid metabolism	Regulation		2
Regulation: SOD2 ---> lipid metabolism	Regulation		2
Regulation: PRKAA1 ---> lipid metabolism	Regulation		2
Regulation: KCNQ1 ---> lipid metabolism	Regulation		2
Regulation: TSPO ---> lipid metabolism	Regulation		2

Fatty acids oxidation

positive Regulation: ESRRA --+> fatty acid oxidation	Regulation	positive	43
positive Regulation: PPARGC1B --+> fatty acid oxidation	Regulation	positive	25
positive Regulation: MAPK14 --+> fatty acid oxidation	Regulation	positive	10
positive Regulation: ABHD5 --+> fatty acid oxidation	Regulation	positive	6
positive Regulation: KLF11 --+> fatty acid oxidation	Regulation	positive	4
positive Regulation: PRKAA1 --+> fatty acid oxidation	Regulation	positive	3
Regulation: ABCA1 ---> fatty acid oxidation	Regulation		3
Regulation: TRIM63 ---> fatty acid oxidation	Regulation		2
positive Regulation: SAA1 --+> fatty acid oxidation	Regulation	positive	2
negative Regulation: PHB ---| fatty acid oxidation	Regulation	negative	2
positive Regulation: NOS2 --+> fatty acid oxidation	Regulation	positive	2

Glucose import

positive Regulation: MAPK14 --+> glucose import	Regulation	positive	71
Regulation: PRKAA1 ---> glucose import	Regulation		14
Regulation: SLC2A5 ---> glucose import	Regulation		11
Regulation: NOS2 ---> glucose import	Regulation		10

<table>
<tr><td>positive Regulation: SOD2 --+> glucose import</td><td>Regulation</td><td>positive</td><td>5</td></tr>
<tr><td>positive Regulation: SPP1 --+> glucose import</td><td>Regulation</td><td>positive</td><td>4</td></tr>
<tr><td>negative Regulation: MLX ---| glucose import</td><td>Regulation</td><td>negative</td><td>4</td></tr>
<tr><td>Regulation: PPARGC1B ---> glucose import</td><td>Regulation</td><td></td><td>4</td></tr>
<tr><td>negative Regulation: GRB10 ---| glucose import</td><td>Regulation</td><td>negative</td><td>4</td></tr>
<tr><td>Regulation: MEF2A ---> glucose import</td><td>Regulation</td><td></td><td>3</td></tr>
<tr><td>Regulation: SLMAP ---> glucose import</td><td>Regulation</td><td></td><td>3</td></tr>
<tr><td>Regulation: ABCA1 ---> glucose import</td><td>Regulation</td><td></td><td>3</td></tr>
<tr><td>negative Regulation: ABHD5 ---| glucose import</td><td>Regulation</td><td>negative</td><td>2</td></tr>
<tr><td>Regulation: GNAI1 ---> glucose import</td><td>Regulation</td><td></td><td>2</td></tr>
<tr><td>Regulation: SAA1 ---> glucose import</td><td>Regulation</td><td></td><td>2</td></tr>
<tr><td>positive Regulation: SLC2A9 --+> glucose import</td><td>Regulation</td><td>positive</td><td>2</td></tr>
<tr><td>positive Regulation: ESRRA --+> glucose import</td><td>Regulation</td><td>positive</td><td>2</td></tr>
</table>

Energy homeostasis

<table>
<tr><td>Regulation: ESRRA ---> energy homeostasis</td><td>Regulation</td><td></td><td>18</td></tr>
<tr><td>Regulation: PRKAA1 ---> energy homeostasis</td><td>Regulation</td><td></td><td>7</td></tr>
<tr><td>negative Regulation: NOS2 ---| energy homeostasis</td><td>Regulation</td><td>negative</td><td>3</td></tr>
<tr><td>Regulation: ABHD5 ---> energy homeostasis</td><td>Regulation</td><td></td><td>3</td></tr>
<tr><td>Regulation: PRKAB2 ---> energy homeostasis</td><td>Regulation</td><td></td><td>2</td></tr>
<tr><td>Regulation: CPE ---> energy homeostasis</td><td>Regulation</td><td></td><td>2</td></tr>
<tr><td>Regulation: GRB10 ---> energy homeostasis</td><td>Regulation</td><td></td><td>2</td></tr>
<tr><td>Regulation: SOD2 ---> energy homeostasis</td><td>Regulation</td><td></td><td>2</td></tr>
<tr><td>Regulation: GK ---> energy homeostasis</td><td>Regulation</td><td></td><td>2</td></tr>
<tr><td>Regulation: PPARGC1B ---> energy homeostasis</td><td>Regulation</td><td></td><td>2</td></tr>
</table>

Gluconeogenesis

<table>
<tr><td>positive Regulation: MAPK14 --+> gluconeogenesis</td><td>Regulation</td><td>positive</td><td>17</td></tr>
<tr><td>negative Regulation: NOS2 ---| gluconeogenesis</td><td>Regulation</td><td>negative</td><td>5</td></tr>
<tr><td>Regulation: GK ---> gluconeogenesis</td><td>Regulation</td><td></td><td>3</td></tr>
<tr><td>positive Regulation: PPARGC1B --+> gluconeogenesis</td><td>Regulation</td><td>positive</td><td>3</td></tr>
<tr><td>Regulation: SPP1 ---> gluconeogenesis</td><td>Regulation</td><td></td><td>3</td></tr>
<tr><td>Regulation: PRKAA1 ---> gluconeogenesis</td><td>Regulation</td><td></td><td>3</td></tr>
<tr><td>Regulation: ESRRA ---> gluconeogenesis</td><td>Regulation</td><td></td><td>3</td></tr>
<tr><td>Regulation: GPD1 ---> gluconeogenesis</td><td>Regulation</td><td></td><td>3</td></tr>
</table>

Regulation: ABCC8 ---> gluconeogenesis	Regulation	2
